# Pharmacological interventions for the prevention of bleeding in people undergoing definitive fixation or joint replacement for hip, pelvic and long bone fractures

**DOI:** 10.1002/14651858.CD013499.pub2

**Published:** 2023-06-05

**Authors:** Victoria N Gibbs, Louise J Geneen, Rita Champaneria, Parag Raval, Carolyn Dorée, Susan J Brunskill, Alex Novak, Antony JR Palmer, Lise J Estcourt

**Affiliations:** Systematic Review InitiativeNHS Blood and TransplantOxfordUK; Nuffield Department of Clinical Laboratory SciencesUniversity of OxfordOxfordUK; Trauma and Orthopaedic Specialist RegistrarUniversity Hospitals of Leicester NHS TrustLeicesterUK; Emergency Medicine Research Oxford (EMROx)Oxford University Hospitals NHS Foundation TrustOxfordUK; Nuffield Department of Orthopaedics, Rheumatology and Musculoskeletal SciencesUniversity of OxfordOxfordUK; Haematology/Transfusion MedicineNHS Blood and TransplantOxfordUK

**Keywords:** Humans, Arthroplasty, Replacement, Fibrinogen, Fractures, Bone, Fractures, Bone/surgery, Hemorrhage, Hemorrhage/chemically induced, Hemorrhage/prevention & control, Hemostatics, Hemostatics/therapeutic use, Myocardial Infarction, Myocardial Infarction/drug therapy, Pulmonary Embolism, Stroke, Stroke/drug therapy, Tranexamic Acid, Tranexamic Acid/therapeutic use, Transfusion Reaction, Venous Thrombosis, Venous Thrombosis/drug therapy

## Abstract

**Background:**

Pelvic, hip, and long bone fractures can result in significant bleeding at the time of injury, with further blood loss if they are treated with surgical fixation. People undergoing surgery are therefore at risk of requiring a blood transfusion and may be at risk of peri‐operative anaemia. Pharmacological interventions for blood conservation may reduce the risk of requiring an allogeneic blood transfusion and associated complications.

**Objectives:**

To assess the effectiveness of different pharmacological interventions for reducing blood loss in definitive surgical fixation of the hip, pelvic, and long bones.

**Search methods:**

We used a predefined search strategy to search CENTRAL, MEDLINE, PubMed, Embase, CINAHL, Transfusion Evidence Library, ClinicalTrials.gov, and the WHO International Clinical Trials Registry Platform (ICTRP) from inception to 7 April 2022, without restrictions on language, year, or publication status.

We handsearched reference lists of included trials to identify further relevant trials. We contacted authors of ongoing trials to acquire any unpublished data.

**Selection criteria:**

We included randomised controlled trials (RCTs) of people who underwent trauma (non‐elective) surgery for definitive fixation of hip, pelvic, and long bone (pelvis, tibia, femur, humerus, radius, ulna and clavicle) fractures only. There were no restrictions on gender, ethnicity, or age.

We excluded planned (elective) procedures (e.g. scheduled total hip arthroplasty), and studies published since 2010 that had not been prospectively registered.

Eligible interventions included: antifibrinolytics (tranexamic acid, aprotinin, epsilon‐aminocaproic acid), desmopressin, factor VIIa and XIII, fibrinogen, fibrin sealants, and non‐fibrin sealants.

**Data collection and analysis:**

Two review authors independently assessed trial eligibility and risk of bias, and extracted data. We assessed the certainty of the evidence using GRADE. We did not perform a network meta‐analysis due to lack of data.

**Main results:**

We included 13 RCTs (929 participants), published between 2005 and 2021. Three trials did not report any of our predefined outcomes and so were not included in quantitative analyses (all were tranexamic acid versus placebo).

We identified three comparisons of interest: intravenous tranexamic acid versus placebo; topical tranexamic acid versus placebo; and recombinant factor VIIa versus placebo. We rated the certainty of evidence as very low to low across all outcomes.

**Comparison 1. Intravenous tranexamic acid versus placebo**

Intravenous tranexamic acid compared to placebo may reduce the risk of requiring an allogeneic blood transfusion up to 30 days (RR 0.48, 95% CI 0.34 to 0.69; 6 RCTs, 457 participants; low‐certainty evidence) and may result in little to no difference in all‐cause mortality (Peto odds ratio (Peto OR) 0.38, 95% CI 0.05 to 2.77; 2 RCTs, 147 participants; low‐certainty evidence).

It may result in little to no difference in risk of participants experiencing myocardial infarction (risk difference (RD) 0.00, 95% CI −0.03 to 0.03; 2 RCTs, 199 participants; low‐certainty evidence), and cerebrovascular accident/stroke (RD 0.00, 95% CI −0.02 to 0.02; 3 RCTs, 324 participants; low‐certainty evidence).

We are uncertain if there is a difference between groups for risk of deep vein thrombosis (Peto OR 2.15, 95% CI 0.22 to 21.35; 4 RCTs, 329 participants, very low‐certainty evidence), pulmonary embolism (Peto OR 1.08, 95% CI 0.07 to 17.66; 4 RCTs, 329 participants; very low‐certainty evidence), and suspected serious drug reactions (RD 0.00, 95% CI −0.03 to 0.03; 2 RCTs, 185 participants; very low‐certainty evidence).

No data were available for number of red blood cell units transfused, reoperation, or acute transfusion reaction.

We downgraded the certainty of the evidence for imprecision (wide confidence intervals around the estimate and small sample size, particularly for rare events), and risk of bias (unclear or high risk methods of blinding and allocation concealment in the assessment of subjective measures), and upgraded the evidence for transfusion requirement for a large effect.

**Comparison 2. Topical tranexamic acid versus placebo**

We are uncertain if there is a difference between topical tranexamic acid and placebo for risk of requiring an allogeneic blood transfusion (RR 0.31, 95% CI 0.08 to 1.22; 2 RCTs, 101 participants), all‐cause mortality (RD 0.00, 95% CI −0.10 to 0.10; 1 RCT, 36 participants), risk of participants experiencing myocardial infarction (Peto OR 0.15, 95% CI 0.00 to 7.62; 1 RCT, 36 participants), cerebrovascular accident/stroke (RD 0.00, 95% CI −0.06 to 0.06; 1 RCT, 65 participants); and deep vein thrombosis (Peto OR 1.11, 95% CI 0.07 to 17.77; 2 RCTs, 101 participants).

All outcomes reported were very low‐certainty evidence.

No data were available for number of red blood cell units transfused, reoperation, incidence of pulmonary embolism, acute transfusion reaction, or suspected serious drug reactions.

We downgraded the certainty of the evidence for imprecision (wide confidence intervals around the estimate and small sample size, particularly for rare events), inconsistency (moderate heterogeneity), and risk of bias (unclear or high risk methods of blinding and allocation concealment in the assessment of subjective measures, and high risk of attrition and reporting biases in one trial).

**Comparison 3. Recombinant factor VIIa versus placebo **

Only one RCT of 48 participants reported data for recombinant factor VIIa versus placebo, so we have not presented the results here.

**Authors' conclusions:**

We cannot draw conclusions from the current evidence due to lack of data. Most published studies included in our analyses assessed the use of tranexamic acid (compared to placebo, or using different routes of administration).

We identified 27 prospectively registered ongoing RCTs (total target recruitment of 4177 participants by end of 2023). The ongoing trials create six new comparisons: tranexamic acid (tablet + injection) versus placebo; intravenous tranexamic acid versus oral tranexamic acid; topical tranexamic acid versus oral tranexamic acid; different intravenous tranexamic acid dosing regimes; topical tranexamic acid versus topical fibrin glue; and fibrinogen (injection) versus placebo.

## Plain language summary

**Are medicines that aim to reduce blood loss during surgery effective in surgeries for trauma of the pelvis, hip, or long bones and do they cause unwanted effects?**

**Key messages**

• We do not yet know the best medicines to reduce bleeding and blood transfusions during surgery for trauma of the pelvis, hip, or long bones (thigh‐bones).

• Some studies are still underway; when they have completed we will hopefully be able to make better conclusions.

**Background**

Fractures of the pelvis, hips and long bones can result in significant bleeding, with further blood loss if surgery is required to fix the fracture. A long bone is a bone that has a shaft and two ends, and is longer than it is wide. This includes bones of the upper and lower leg, arms, and collarbone. Fractures and subsequent surgery bring a risk of blood transfusion and anaemia. Anaemia is when the number of red blood cells or the haemoglobin concentration within them is lower than normal. Haemoglobin carries oxygen round the body ‐ low haemoglobin levels cause symptoms such as fatigue, weakness, dizziness and shortness of breath.

**Why is it important to reduce blood transfusions during vascular surgery?**

If people bleed a lot during or after this type of surgery they may need blood transfusions to replace the blood they have lost. It is better to avoid receiving a blood transfusion, if possible, because blood transfusions can cause harm. This is especially important when health services have limited blood supplies. Medicines may reduce the need for a blood transfusion and its associated complications, improve patient outcomes, and decrease healthcare costs. Examples of such medicines are tranexamic acid and recombinant factor VIIa. However, they may cause unwanted effects, such as blood clots.

**What did we want to find out?**

We wanted to discover if there are any medicines that help to reduce blood loss during surgery to fix fractures in the pelvis, hip, or long bones in adults. We also wanted to find out which of the effective medicines was the most effective. Reducing blood loss reduces the risk of anaemia and requiring a blood transfusion. It can also reduce the risk of requiring another operation to stop the bleeding or to remove a large collection of blood (haematoma) due to previous bleeding.

**What did we do?**

We searched for studies that investigated using medicines to prevent blood loss in this kind of surgery.

**What did we find? **

We found 13 studies with 929 people, published between 2005 and 2022. Most studies assessed the effectiveness and safety of tranexamic acid, whether used intravenously (injected into a vein), locally (topically ‐ directly onto the site of the injury), or a combination of the two. Only one study looked at recombinant factor VIIa. Both medicines help the blood to clot.

**Main results**

***Intravenous tranexamic acid***

Intravenous tranexamic acid may reduce the need for blood transfusion slightly, and it may result in little to no difference in the risk of death from any cause and the number of people who experience a heart attack, or stroke.

We are uncertain if intravenous tranexamic acid has any impact on the risk of blood clots that form in the veins of the leg (deep vein thrombosis (DVT)), or lungs (pulmonary embolism), or suspected serious reactions to the medicine. There was no evidence to show whether it affected the need for reoperation due to bleeding, or the number of people who had an immediate reaction to blood transfusion.

***Topical tranexamic acid***

We are uncertain if topical tranexamic acid affects the need for blood transfusion, deaths from any cause, or the number of people who experience a heart attack, stroke, or DVT. There was no evidence to show whether it affected the need for reoperation for bleeding, or the number of people with pulmonary embolism, severe reactions to blood transfusion, or suspected serious reactions to the medicine.

***Recombinant factor VIIa***

We are uncertain if recombinant factor VIIa has any impact on the need for blood transfusion, the need for reoperation for bleeding, the risk of DVT, pulmonary embolism, or suspected serious reaction to the medicine. There was no evidence to assess whether it impacted deaths from any cause, the risk of heart attack, stroke, or immediate reaction to the medicine.

**What are the limitations of the evidence?**

We have little confidence in the evidence for some outcomes, and are not confident about the evidence for others. This is because it is possible that people in the studies were aware of which treatment they were getting, also, the studies were small, and did not all provide data about everything in which we were interested.

**Ongoing studies and future updates**

Twenty‐seven studies with a planned total of 4177 participants are currently ongoing. These should be completed and published within the next few years. Once they publish their data, we can update our analyses and probably provide stronger answers than we can now.

**How up to date is this evidence**

The evidence is current to 7 April 2022.

## Summary of findings

**Summary of findings 1:** Intravenous tranexamic acid versus placebo

**Intravenous tranexamic acid compared to placebo for the prevention of bleeding in people undergoing definitive fixation of hip, pelvic and long bone fractures**						
**Population:** people undergoing definitive fixation of hip, pelvic and long bone fractures **Setting:** inpatients **Intervention:** intravenous tranexamic acid **Comparison:** placebo						
**Outcomes**	Anticipated absolute effects^*^ (95% CI)		**Relative effect (95% CI)**	**№ of participants (studies)**	**Certainty of the evidence (GRADE)**	**Comments**
	**Risk with placebo**	**Risk with TXA (IV)**				
**Risk of requiring allogeneic blood transfusion (30 days post‐surgery)**	511 per 1000	**245 per 1000** (174 to 352)	**RR 0.48** (0.34 to 0.69)	457 (6 RCTs)	⨁⨁◯◯ Low^a^	TXA (IV) may reduce the risk of requiring allogeneic blood transfusion up to 30 days post‐surgery (Analysis 1.1)
**All‐cause mortality (30 days post‐surgery)**	39 per 1000	**15 per 1000** (2 to 102)	**Peto OR 0.38** (0.05 to 2.77)^b^	147 (2 RCTs)	⨁⨁◯◯ Low^,c,d^	TXA (IV) may result in little to no difference in all‐cause mortality up to 30 days post‐surgery (Analysis 1.2)
**Re‐operation due to bleeding (7 days post‐surgery) ‐ not reported**	‐	‐	‐	‐	‐	No included studies reported this outcome
**Risk of myocardial infarction (30 days post‐surgery)**	0 per 1000	**0 per 1000** (0 to 0)	**RD 0.00** (‐0.03 to 0.03)^e^	199 (2 RCTs)	⨁⨁◯◯ Low^c,f^	TXA (IV) may result in little to no difference in risk of MI up to 30 days post‐surgery (Analysis 1.3)
**Risk of cerebrovascular accident/stroke (30 days post‐surgery)**	0 per 1000	**0 per 1000** (0 to 0)	**RD 0.00** (‐0.02 to 0.02)^e^	324 (3 RCTs)	⨁⨁◯◯ Low^c,f^	TXA (IV) may result in little to no difference in risk of CVA/stroke up to 30 days post‐surgery (Analysis 1.4)
**Risk of deep vein thrombosis (30 days post‐surgery**)	6 per 1000	**13 per 1000** (1 to 114)	**Peto OR 2.15** (0.22 to 21.35)^b^	329 (4 RCTs)	⨁◯◯◯ Very low^g,h^	Very low‐certainty evidence means we are uncertain whether TXA (IV) makes any difference in the risk of DVT (Analysis 1.5)
**Risk of suspected serious drug reactions (30 days post‐surgery)**	0 per 1000	**0 per 1000** (0 to 0)	**RD 0.00** (‐0.03 to 0.03)^e^	185 (2 RCTs)	⨁◯◯◯ Very low^f,g^	Very low‐certainty evidence means we are uncertain whether TXA (IV) makes any difference in the risk of suspected drug reactions (Analysis 1.7)
*The risk in the intervention group (and its 95% confidence interval) is based on the assumed risk in the comparison group and the **relative effect** of the intervention (and its 95% CI). **CI:** confidence interval; **CVA:** cerebrovascular accident; **DVT:** deep vein thrombosis; **IV:** intravenous; **MI:** myocardial infarction; **Peto OR:** Peto odds ratio; **RD:** risk difference; **RR:** risk ratio; **TXA:** tranexamic acid						
**GRADE Working Group grades of evidence** **High certainty:** we are very confident that the true effect lies close to that of the estimate of the effect. **Moderate certainty:** we are moderately confident in the effect estimate: the true effect is likely to be close to the estimate of the effect, but there is a possibility that it is substantially different. **Low certainty:** our confidence in the effect estimate is limited: the true effect may be substantially different from the estimate of the effect. **Very low certainty:** we have very little confidence in the effect estimate: the true effect is likely to be substantially different from the estimate of effect.						

**Explanations** ^a^Downgraded twice for risk of bias as this is a subjective outcome and nearly all assessments of blinding are high or unclear, and unclear assessments for most studies for allocation concealment. ^b^Peto odd ratio used due to low event rate (< 5%) in each arm. ^c^Did not downgrade for risk of bias as this is an objective outcome and less likely to be impacted by lack of blinding and allocation concealment. ^d^Downgraded twice for imprecision due to very wide confidence intervals. ^e^Risk difference used due to zero cases in both arms. ^f^Downgraded twice for imprecision due to the very small sample size, far below the optimal information size for this outcome. ^g^Downgraded once for risk of bias as this is a subjective outcome with unclear assessment for some blinding. ^h^Downgraded three times for imprecision due to extremely wide confidence intervals and small sample size, far below optimal information size for this outcome.

**Summary of findings 2:** Topical tranexamic acid versus placebo

**Topical tranexamic acid compared to placebo for the prevention of bleeding in people undergoing definitive fixation of hip, pelvic and long bone fractures**						
**Population:** people undergoing definitive fixation of hip, pelvic and long bone fractures **Setting:** inpatients **Intervention:** topical tranexamic acid **Comparison:** placebo						
**Outcomes**	Anticipated absolute effects^*^ (95% CI)		**Relative effect (95% CI)**	**№ of participants (studies)**	**Certainty of the evidence (GRADE)**	**Comments**
	**Risk with placebo**	**Risk with TXA (topical)**				
**Risk of requiring allogeneic blood transfusion (30 days post‐surgery)**	189 per 1000	**58 per 1000** (15 to 230)	**RR 0.31** (0.08 to 1.22)	101 (2 RCTs)	⨁◯◯◯ Very low^a,b^	Very low‐certainty evidence means we are uncertain whether TXA (topical) makes any difference in the risk of requiring allogeneic blood transfusion up to 30 days post‐surgery (Analysis 2.1)
**All‐cause mortality (30 days post‐surgery)**	0 per 1000	**0 per 1000** (0 to 0)	**RD 0.0** (‐0.10 to 0.10)^c^	36 (1 RCT)	⨁◯◯◯ Very low^a,d^	Very low‐certainty evidence means we are uncertain whether TXA (topical) makes any difference in all‐cause mortality up to 30 days post‐surgery. Analysis 2.2
**Re‐operation due to bleeding (7 days post‐surgery) ‐ not reported**	‐	‐	‐	‐	‐	No included studies reported this outcome
**Risk of myocardial infarction (30 days post‐surgery)**	53 per 1000	**8 per 1000** (0 to 297)	**Peto OR 0.15** (0.00 to 7.62)^e^	36 (1 RCT)	⨁◯◯◯ Very low^a,b^	Very low‐certainty evidence means we are uncertain whether TXA (topical) makes any difference in the risk of MI up to 30 days post‐surgery (Analysis 2.3)
**Risk of cerebrovascular accident/stroke (30 days post‐surgery)**	0 per 1000	**0 per 1000** (0 to 0)	**RD 0.00** (‐0.06 to 0.06)^c^	65 (1 RCT)	⨁◯◯◯ Very low^d^	Very low‐certainty evidence means we are uncertain whether TXA (topical) makes any difference in the risk of CVA/stroke up to 30 days post‐surgery (Analysis 2.4)
**Risk of deep vein thrombosis (30 days post‐surgery)**	19 per 1000	**21 per 1000** (1 to 255)	**Peto OR 1.11** (0.07 to 17.77)^e^	101 (2 RCTs)	⨁◯◯◯ Very low^a,b,f^	Very low‐certainty evidence means we are uncertain whether TXA (topical) makes any difference in the risk of DVT up to 30 days post‐surgery (Analysis 2.5)
**Risk of suspected serious drug reactions (30 days) ‐ not reported**	‐	‐	‐	‐	‐	No included studies reported this outcome
*The risk in the intervention group (and its 95% confidence interval) is based on the assumed risk in the comparison group and the **relative effect** of the intervention (and its 95% CI). **CI:** confidence interval; **CVA:** cerebrovascular accident; **DVT:** deep vein thrombosis; **MI:** myocardial infarction; **Peto OR:** Peto odds ratio; **RD:** risk difference; **RR:** risk ratio; **TXA:** tranexamic acid						
**GRADE Working Group grades of evidence** **High certainty:** we are very confident that the true effect lies close to that of the estimate of the effect. **Moderate certainty:** we are moderately confident in the effect estimate: the true effect is likely to be close to the estimate of the effect, but there is a possibility that it is substantially different. **Low certainty:** our confidence in the effect estimate is limited: the true effect may be substantially different from the estimate of the effect. **Very low certainty:** we have very little confidence in the effect estimate: the true effect is likely to be substantially different from the estimate of effect.						

**Explanations** ^a^Downgraded once for risk of bias due to high and unclear assessments for other biases, and high risk for attrition and reporting bias in one trial. ^b^Downgraded twice for imprecision due to very wide confidence intervals and small sample size. ^c^Risk difference used due to zero cases in both arms. ^d^Downgraded three times for imprecision due to very small sample size in an outcome with rare events. ^e^Peto OR used due to low event rate (< 5%) in each arm. ^f^Downgraded once for inconsistency due to moderate heterogeneity (I² = 51%, Chi² = 2.02, P = 0.15).

## Background

### Description of the condition

Traumatic injury and fracture is one of the world's leading causes of death and disability (Haagsma 2016). Acute orthopaedic injuries, including soft tissue, muscle and bone injuries, are the most common injuries sustained in accidents and the most likely form of traumatic injury to require hospitalisation (Clay 2010; Lang 2014; Lee 2005). In addition, orthopaedic injury may result in important individual and social disability and is associated with substantial economic and social costs (Clay 2010; Lang 2014; Williamson 2009).

Age and gender are the strongest risk factors for fracture. Older people are more likely to have lower bone mineral density and osteoporosis and therefore lower energy accidents such as a fall from standing height may result in a significant injury such as a hip fracture. Younger people tend to have a higher bone mineral density and therefore higher impact accidents may result in fracture (Armas 2010). In 2010, the number of people aged 50 years or older at high risk of osteoporotic fracture worldwide was estimated at 158 million and this figure is expected to double by 2040 (Odén 2015). As a consequence of an ageing population, globally the number of people with a hip fracture is expected to reach 6.26 million by 2050 (Dhanwal 2011). Studies in the UK report incidences of pelvic fracture in the region of 7.4 per 10,000; tibial fractures 8.8 per 10,000; and radius/ulna fractures 9.6 per 10,000 for men, and 41.2 per 10,000 for women (van der Velde 2016). Hip fractures are more common, with the incidence reported as between 46.7 to 35.7 per 10,000 (Nordström 2022). 

Pelvic, hip and long bone fractures can result in significant bleeding. Blood loss from a closed femoral fracture is estimated to be between 1000 mL and 1500 mL, and for closed tibial fractures between 500 mL and 1000 mL. For open fractures, when the skin is breached, these figures may double (Lee 2005). Surgical fixation techniques include plate and screws, intramedullary nailing (a rod placed down the middle of the bone) or joint replacement. Determining which technique to use depends on the location of the injury, type of fracture and functional requirements of the person. Surgical fixation of pelvic, hip and long bones may result in a large volume of blood loss and this is in addition to the initial loss at the time of injury. Hip hemiarthroplasty for fracture (half a hip replacement whereby the ball of the femur is replaced, and the socket is left alone) results in around 800 mL of blood from surgery (Guo 2018). For people undergoing revision total hip replacement for periprosthetic fracture (whereby the person has sustained a fracture around an existing hip replacement), intraoperative blood loss from surgery is around 1000 mL (Palmer 2020). Long bone fixation with plate and screws or fixation with an intramedullary nail is thought to incur a blood loss between 550 mL and 1500 mL (Foss 2006; Xu 2021), while the estimated blood loss for pelvic fixation with plate and screws is thought to be around 1200 mL (Odak 2013). Hip fractures treated with dynamic hip screw fixation typically result in a lower blood loss of between 300 mL to 400 mL (Baruah 2016), and fixation of humerus fractures results in blood loss of around 150 mL (Wang 2020). Fixation of extremity fractures, such as fibula and radius fractures, results in even lower blood losses of around 90 mL to 120 mL (Taylor 2015), and 100 mL (Wei 2016), respectively.

In a Cochrane Review of hip fracture surgery, taking a liberal haemoglobin transfusion threshold of approximately 100 g/L, 74% to 100% of people who had surgery for a neck‐of‐femur fracture required a blood transfusion, and for a restrictive haemoglobin transfusion threshold of approximately 80 g/L, 11% to 45% of people required a blood transfusion (Brunskill 2015). Allogeneic blood transfusions (donated blood from matched donors) are not without risk and have been shown to increase the risk of mortality and morbidity (Arshi 2020). In addition, allogeneic transfusion is associated with increased duration of hospital stay, which increases healthcare costs (Smeets 2018).

Presently, there are several effective pharmacological interventions available that help prevent blood loss during surgery (Schulman 2012). Pharmacological interventions offer the opportunity to reduce the risk of allogeneic blood transfusion and associated complications, improve outcomes and decrease healthcare costs.

### Description of the intervention

This review focuses on pharmacological interventions used to reduce bleeding during surgery to fix fractured bones to allow them to heal (definitive fixation). Pharmacological interventions to prevent bleeding provide the opportunity to reduce blood transfusion and the infection and compatibility complications associated with its use. The interventions of interest for this review include antifibrinolytic drugs, desmopressin, factor VIIa and factor XIII, fibrinogen, and sealants (glues).

Antifibrinolytic interventions include tranexamic acid, aprotinin and epsilon‐aminocaproic acid. Tranexamic acid and epsilon‐aminocaproic acid are synthetic derivatives of lysine, while aprotinin is derived from bovine lung. Antifibrinolytics help to reduce blood loss through stabilising blood clots and reduce bleeding in major trauma, particularly when given early (Ker 2015).

Sealants (which are applied directly to the wound during surgery) can be grouped into those that contain fibrin and those that do not contain fibrin. Fibrin plays an important role in forming a blood clot, and sealants containing fibrin prevent bleeding during surgery. They are thought to be particularly effective when used in orthopaedic surgery where blood loss is high (Carless 2003). Non‐fibrin sealants rely on fibrin found in normal blood, and tend to exert their effects through mechanical expansion, which provides pressure to bleeding surfaces (Baird 2015).

The route by which the interventions can be administered is displayed in Table 3 and includes intravenous, oral, topical and nasal modes.

**1:** Table of intervention variables

**Variable^a^**	**TXA**	**Aprotinin**	**Epsilon‐aminocaproic acid**	**Desmopressin**	**Factor VIIa**	**Factor XIII**	**Fibrinogen**	**Fibrin sealants/glue**	**Non‐fibrin sealants**
**Timing**									
Preoperative	✓^b^	✓	✓	✓	✓	✓	✓	X^c^	X
Intraoperative	✓	✓	✓	✓	✓	✓	✓	✓	✓
Postoperative	✓	X	X	✓	✓	✓	✓	X	X
**Route**									
IV (injection, infusion)	✓	✓	✓	✓	✓	✓	✓	X	X
Topical	✓	X	X	X	X	X	X	✓	✓
Intranasal	X	X	X	✓	X	X	X	X	X
Subcutaneous injection	X	X	X	✓	X	X	X	X	X
IV + topical	✓	X	X	X	X	X	X	X	X
Oral	✓	X	✓	X	X	X	X	X	X
IV + oral	✓	X	X	X	X	X	X	X	X
Topical + oral	✓	X	X	X	X	X	X	X	X
**Dose**									
Single	✓	X	✓	✓	✓	✓	✓	✓	✓
Multiple	✓	✓	X	✓	✓	✓	✓	X	X
Variable units/kg	✓	X	✓	X	✓	✓	✓	X	X
Variable trial‐set dose	✓	✓	X	✓	✓	✓	✓	✓	✓
** IV:** intravenous; **TXA:** tranexamic acid									

^a^The table is for illustrative purposes only and replicated from Gibbs 2019b. ^b^Ticks indicate which intervention and timing/route/dose combinations are clinically possible. ^c^Crosses indicate which intervention and timing/route/dose combinations are not clinically possible.

### How the intervention might work

Blood loss from surgical fixation of fractures causes haemoglobin levels to fall and blood transfusion may be required to optimise oxygen delivery to tissues, even though it is associated with risk. The aim of the interventions to conserve blood (listed below) is to reduce bleeding, and ultimately reduce blood loss and need for blood transfusion.

An explanation of how each intervention works with any potential risks is provided below.

#### Antifibrinolytics (tranexamic acid, aprotinin and epsilon‐aminocaproic acid)

Antifibrinolytics act by inhibiting the process that breaks down blood clots, resulting in the clot becoming more stable (Tengborn 2015). The most commonly used antifibrinolytics are tranexamic acid, aprotinin and epsilon‐aminocaproic acid (Henry 2011). They may be administered orally, intravenously or topically (BNF 2022). Although most of these drugs cause few adverse effects, there is a theoretically greater risk of unwanted venous blood clots with their use (Levy 2018; Myers 2019), and at higher doses there is concern about the risk of seizures (Zhang 2016).

#### Desmopressin

Desmopressin stimulates the release of factor VIII (Pearson 2016), which in turn encourages blood clotting. Factor VIII, an important factor contained in blood, enables platelets to adhere to wound sites and form blood clots. It can be given intravenously, subcutaneously (under the skin) or intranasally (via the nose) (BNF 2022). Reported adverse effects include facial flushing, and the possibility of low blood sodium levels, particularly with repeated doses (Desborough 2017).

#### Recombinant factor VIIa and factor XIII

Recombinant factor VIIa is used to treat people with haemophilia, congenital factor VII deficiency and inhibitory alloantibodies. It has also been administered outside licensed use (off‐licence) to prevent significant blood loss during surgery (Simpson 2012). However, despite its use, the efficacy of this drug in people who do not have haemophilia remains unclear.

Recombinant factor XIII protects a developing clot during formation and, therefore, improves clot strength. This effect is likely to depend on dose, and it has been suggested that maintaining high levels of recombinant factor XIII may prevent bleeding (Aleman 2014).

Both recombinant factor V11a and XIII are administered intravenously (BNF 2022). The concern with recombinant factor V11a is the potential increased risk of arterial blood clots, particularly in older people; however, there is limited evidence to confirm this risk (Goodnough 2016).

#### Fibrinogen

Fibrinogen is a soluble protein present in the bloodstream. During tissue and vessel injury it is converted by enzymes to fibrin (by thrombin) and then to a fibrin‐based blood clot. The formation of the blood clot helps to prevent excessive bleeding. Fibrinogen is administered intravenously (BNF 2022). Since fibrinogen is obtained from blood, there is a potential risk, albeit small, of viral infection due to the manufacturing process (Franchini 2012).

#### Fibrin sealants

Fibrin sealants are surgical wound adhesives and are administered topically. They are mostly used during surgery and to aid haemostasis (halt bleeding), tissue sealing and wound healing. Sealants tend to originate from plasma and commonly contain fibrinogen, thrombin, factor XIII and calcium chloride. Fibrin sealants may include an antifibrinolytic agent (Fischer 2011), and their final composition may vary. They can be applied to actively bleeding bony surfaces and into the wound. Allergy is a rarely noted adverse effect (Aguilera 2013).

#### Non‐fibrin sealants

Non‐fibrin sealants are administered topically and tend to be liquids that combine to form a film that promotes platelet activation and formation of a cluster. Non‐fibrin sealants help with blood clot formation, however, the functioning of the sealant is dependent on the individual's own fibrin contained within their blood. The term 'non‐fibrin sealants' also encompasses internal dressings and powders, which may be an alternative to tourniquet use when this is not possible. The mechanism of action of many sealants in this group is through mechanical expansion and compression of tissues. Consequently, many reported adverse events are associated with this, including nerve compression (Baird 2015).

### Why it is important to do this review

This review assesses the effectiveness of various pharmacological interventions to prevent blood loss following definitive fixation of hip, pelvic and long bone fractures (definitive meaning a permanent fix of the broken bone as opposed to a temporary surgery). Although emergency blood transfusions provide a life‐saving treatment for people who have lost blood from trauma, there are risks associated with allogeneic blood transfusions, such as transfusion‐transmitted infection and serious adverse transfusion reactions (WHO 2016). In 2017 in the UK, 21 people died from transfusion‐related complications and there were 112 incidences of major morbidity associated with blood transfusion (SHOT 2018).

A global priority for the World Health Organization (WHO) is to be able to provide safe access to blood products, and also to minimise unnecessary transfusions in order to preserve a scarce resource, reduce risk, and reduce costs (WHO 2016). One unit of red blood cells in the UK cost GBP 129 in April 2019, rising to GBP 133 by 2020 (NCG 2018). By comparison, in 2018, an ampoule of tranexamic acid cost GBP 1.50, and an ampoule of desmopressin cost GBP 13.16 (BNF 2022). Embracing pharmacological treatments to prevent bleeding may reduce the need for blood transfusion, reduce costs, and potentially offer people undergoing surgery a lower risk profile.

Concerns around the adverse effect profile of pharmacological interventions may contribute to their limited uptake in clinical practice for people who require definitive fixation. Theoretically, interventions to prevent bleeding may also result in the formation of unwanted blood clots. This may be of particular concern in people with myocardial infarction or a pre‐existing increased risk of stroke or pulmonary embolism (Danninger 2015). Knowing the optimal dose could help to limit adverse effects, as well as reduce treatment costs. In addition, the timing of the intervention is important. The CRASH‐2 trial (Clinical Randomisation of an Antifibrinolytic in Significant Haemorrhage 2; a large randomised controlled trial (RCT) of tranexamic acid versus placebo in people with major trauma) found that timing of the intervention was associated with outcome (Roberts 2013). Delivery of tranexamic acid within three hours of trauma improved the chance of survival, however, when tranexamic acid was delivered three hours after injury, there was an increased risk of death from bleeding.

Currently, the optimal dose, route, and timing of these interventions is unknown, which results in uncertainty for decision makers. 

#### Description of network meta‐analysis (NMA)

A network meta‐analysis (NMA) is a type of analysis that allows more than two treatments to be compared (Lu 2004). Network diagrams are used to represent the available evidence for each treatment comparison. Each treatment is represented by a node (vertex), and a line is used to connect the two treatments being compared (Jansen 2011). It is important to undertake an NMA like any other meta‐analysis, using a rigorous systematic approach. Network diagrams contain a mix of solid and blank lines. Solid lines indicate 'direct' comparisons for which there is evidence from clinical trials. Blank (or absent lines) indicate 'indirect' comparisons, that is, those where no clinical trials have compared the interventions (Bucher 1997; Jansen 2011).

An NMA uses data from direct comparisons to estimate the effects of indirect comparisons that have not been assessed yet in a clinical trial (Caldwell 2005; Jansen 2011; Jansen 2013; Song 2003). This allows an NMA to 'fill gaps' in the evidence by pooling data from direct clinical trial comparisons, and to deduce information about missing comparisons in the network (Krahn 2013; Salanti 2014). To draw robust conclusions, the NMA assumes that all the people and trials included in the network are similar enough in terms of effect modifiers across all direct comparisons (Jansen 2013).

A further benefit of NMA is that it can aid clinical decision making by providing results in an accessible format. Outputs can be tabulated in a hierarchy to show results by treatment and outcome. This is particularly useful as all relevant evidence can be included in one table, indicating both benefits and risks of a given treatment (Hoaglin 2011; Jansen 2011; Sutton 2008; van der Valk 2009).

Whilst we intended to perform NMA, we were unable to for this review due to the lack of data. A description of NMA methods to be used in future updates is available in the original published protocol (Gibbs 2019a) and in Appendix 1.

## Objectives

To assess the effectiveness of different pharmacological interventions for reducing blood loss in definitive surgical fixation of the hip, pelvic, and long bones.

## Methods

### Criteria for considering studies for this review

#### Types of studies

We included randomised controlled trials (RCTs). If the process of randomisation was unclear, we contacted the trial authors to obtain further information. If we were unable to contact the trial authors, we included the trial in the review and considered it to be at unclear risk of bias. To be eligible, trials had to compare at least one of our interventions of interest (placebo versus active treatment, or active treatment versus another active treatment). We used both abstracts and full‐text publications if they reported adequate information about study design, participant characteristics and interventions. 

We planned to include cluster‐randomised trials if they had at least two intervention sites and two control sites. We excluded cluster‐randomised trials that had only one intervention or control site because the intervention (or comparison) may be confounded by study site making it difficult to attribute any observed differences to the intervention rather than to other site‐specific variables.

We did not include quasi‐RCTs (assigned to a treatment, procedure, or intervention by methods that are not random) due to the potential for significant confounding and lack of proper randomisation.

We only included trials that had been prospectively registered, unless the final trial report was published before 2010. The decision to exclude unregistered (or retrospectively registered) trials was taken due to the evidence highlighting issues surrounding false data (Carlisle 2021; Roberts 2015), and has now become policy of Cochrane Injuries (Broughton 2021; Cochrane policy). Prospective registration reduces the chance of publication bias, and has been compulsory for RCTs since 2005, suggesting that those that have not been registered (or were registered retrospectively) since then are less likely to be of high quality (Roberts 2015). We have used a cut‐off of 2010 as this allowed studies that commenced before the introduction of compulsory registration in 2005 to complete and publish.

#### Types of participants

We included people who have undergone trauma (non‐elective) surgery for definitive fixation of hip, pelvic, and long bone (pelvis, tibia, femur, humerus, radius, ulna and clavicle) fractures. 

We excluded people undergoing surgeries as planned (elective) procedures (e.g. scheduled total hip arthroplasty). There were no restrictions on gender, ethnicity, or age.

Definitive fixation included the following types of surgery:

- fixation with plate and screws, intramedullary nailing and joint replacement;
- joint replacement surgery:
  - hip hemiarthroplasty;
  - total hip replacement;
  - total shoulder replacement;
  - reverse shoulder replacement;
  - total knee replacement; and
  - total elbow replacement for the management of fractures;
- fixation of a fracture around an existing replacement (periprosthetic fractures).

If an eligible trial contained a mixed population of people (e.g. non‐definitive surgery such as temporary external fixation), then we only used data contributed from our population of interest. If no subgroup data were given, and we were unable to contact the corresponding author to provide this information, at least 80% of the sample size had to be from our population of interest for the trial to be eligible for inclusion. 

We included participants if they were taking anticoagulant medication or antiplatelet therapy at the time of injury. We excluded participants with known bleeding disorders, such as haemophilia.

#### Types of interventions

Eligible trials have compared one or more of the following interventions:

- antifibrinolytics:
  - tranexamic acid;
  - aprotinin;
  - epsilon‐aminocaproic acid;
- desmopressin;
- recombinant factor VIIa and factor XIII;
- fibrinogen;
- fibrin sealants; and
- non‐fibrin sealants.

We did not combine different interventions and treatments other than those listed above. Trials had to compare an intervention of interest versus placebo, or an intervention of interest versus another intervention of interest. We included trials that used interventions of interest combined with another agent or blood product in each arm (e.g. tranexamic acid plus platelets versus placebo plus platelets), as we consider the effect of the additional agent in both arms will cancel out.

To explore the optimal treatment pathway, we considered interventions administered over a range of doses, as both single or multiple doses via intravenous, subcutaneous, intranasal, oral or topical routes, and at different timings.

The variations in dose, route, and times for interventions may differ greatly.

#### Types of outcome measures

We did not use the reporting of certain outcomes as criteria for including studies. If the study did not report any of our listed outcomes, it remained included if it fulfilled all other inclusion criteria.

We planned to use the outcome measures below to assess the relative hierarchy of our interventions as part of the NMA, however we have only performed direct pairwise analyses and are therefore unable to create a hierarchy. See the original protocol (Gibbs 2019a), and Appendix 1 for further information regarding the NMA methods to be used in future updates.

##### Primary outcomes

- Risk of participants receiving allogeneic blood transfusions during or after surgery (up to 30 days)
- All‐cause mortality (deaths occurring up to 30 days after the operation)

##### Secondary outcomes

- Mean number of red blood cell units transfused per person (within 30 days)
- Reoperation due to bleeding (within 7 days)
- Adverse events:
  - thromboembolism (deep vein thrombosis, pulmonary embolism, myocardial infarction, stroke) (within 30 days)
  - transfusion reactions (acute) (within 24 hours)
  - suspected serious adverse drug reactions (within 30 days)

For suspected serious adverse drug reactions we used the International Conference on Harmonisation Good Clinical Practice definition of a serious adverse drug reaction (ICH GCP 2018). 

We also planned to collect and present any data on cost or resource information reported in the included trials. However, we found no trials that presented this information in a usable way.

### Search methods for identification of studies

The Information Specialist (CD) from the Systematic Review Initiative performed the search in conjunction with Cochrane Injuries.

We searched for all relevant published and unpublished trials without restrictions on language, year, or publication status.

#### Electronic searches

##### Bibliographic databases

We produced thorough and sensitive search strategies to identify RCTs and systematic reviews in the following databases, from database inception to the date of search:

- Cochrane Central Register of Controlled Trials (CENTRAL; 2022, issue 3) via the Cochrane Library;
- MEDLINE (OvidSP, 1946 to 7 April 2022);
- PubMed (NLM, for e‐publications ahead of print only)
- Embase (OvidSP, 1974 to 7 April 2022);
- CINAHL (EBSCOhost, 1937 to 7 April 2022);
- Transfusion Evidence Library (Evidentia Publishing, 1950 to 7 April 2022); 
- ClinicalTrials.gov from inception to 7 April 2022;
- World Health Organization International Clinical Trials Registry Platform (ICTRP) from inception to 7 April 2022.

The searches were combined in the MEDLINE, Embase and CINAHL databases with adaptations of the recommended Cochrane RCT filter (Lefebvre 2022), and of the Scottish Intercollegiate Guidelines Network (SIGN) systematic review filters (www.sign.ac.uk).  

Search strategies for all databases are presented in Appendix 2.

#### Searching other resources

We handsearched reference lists of included trials in order to identify further relevant trials. We also contacted authors of ongoing trials to acquire any unpublished data. We contacted trial authors a maximum of three times.

### Data collection and analysis

We performed the systematic review using methods stated in the *Cochrane Handbook for Systematic Reviews of Interventions* (Higgins 2022a). We used Review Manager 5 (Review Manager 2020). As we did not undertake an NMA, we did not use Stata (Stata 2017).

#### Selection of studies

At least two of the review authors (LJG, SJB, PR, VNG, RC) independently screened titles and abstracts of citations identified by the electronic searches for eligibility. If the title and abstract of the citation was found to be irrelevant, we excluded it at this stage. The same review authors then independently screened the full‐text articles of the citations thought to be eligible against the criteria set out in the review's protocol (Gibbs 2019a). We resolved disagreements through discussion, or through consultation with another review author (LJE). 

Where there was insufficient information with which to make a decision regarding eligibility, we requested further information from the corresponding author of the trial. We contacted the author up to three times within six weeks (see Appendix 3). If there was no response after six weeks of initial attempted contact, we added the study to Characteristics of studies awaiting classification. We kept records of the study selection process and used the information to generate a PRISMA flowchart to show the flow of studies (Moher 2009). We recorded the reasons why potentially‐relevant studies failed to meet the eligibility criteria.

Translations were provided by colleagues, or we used Cochrane resources such as TaskExchange.

#### Data extraction and management

At least two review authors (LJG, SJB, VNG, RC) extracted the data according to Cochrane guidelines (Li 2020). We resolved disagreements by consensus, or through arbitration by another review author (LJE). We extracted data independently for all the trials using a piloted extraction form in Covidence, modified to reflect the outcomes in this review.  The review authors were not blinded to authors, institutions, or outcomes of the trials they were extracting. 

We contacted corresponding authors up to three times to request further trial data, and classified the data as unobtainable if there was no response from the authors within six weeks of the initial email request. 

See Table 3 for the potential dose, route, and timing combinations for each intervention. 

We extracted data for the following items and list these and the outcomes from each trial in the Characteristics of included studies.

- **General information:** name of review author carrying out data extraction, date of data extraction, study identifier, surname and contact address of first author, language of trial
- **Trial information:** RCT trial design – location of where the trial was run, setting, sample size, duration of trial, power calculation, treatment arms, randomisation, inclusion and exclusion criteria, comparability of groups, length of study
- **Characteristics of participants:** age, sex, breakdown of total numbers for those randomised and analysed, type of surgery, dropouts (percentage in each arm) with reasons and protocol violations, participants on anticoagulants or antiplatelet therapy at the time of injury, participants given tranexamic acid in the pre‐hospital setting or on admission to the emergency department, duration of surgery, use of tourniquet and type of anaesthetic (spinal or general)
- **Characteristics of interventions:** number of treatment arms, description of experimental arm(s), description of control arm(s), timing, dose and route of administration of intervention, and other differences between intervention arms
- **Outcomes (all within 30 days of surgery unless otherwise specified):** allogeneic blood transfusion during or after surgery, mortality due to any cause, mean number of units of red blood cells transfused, reoperation due to bleeding (within 7 days) and adverse effects (thromboembolism, transfusion reactions (within 24 hours) and adverse drug reactions). We used the International Conference on Harmonisation Good Clinical Practice definition of serious adverse events (ICH GCP 2018). Where that definition was not used in the included studies we extracted information about how each study defined 'adverse effect' and 'serious adverse effect'
- **Quality assessment:** allocation concealment, blinding (participants, personnel, outcome assessors), incomplete outcome data, selective outcome reporting, other sources of bias.

We used both full‐text versions and abstracts as data sources and used one data extraction form for each unique study. Where sources did not provide sufficient information, we contacted trial authors for additional details.

No studies presented data on cost, resource usage, or quality of life.

Two review authors (RC, LJG) entered data into Review Manager 5 (Review Manager 2020), and resolved any disagreements by consensus. 

##### Potential risk modifiers

We extracted data on characteristics that may behave as treatment risk modifiers in a future review update where NMA is performed (details of potential risk modifier can be found in the original protocol (Gibbs 2019a), and Appendix 1). We took the decision to present only direct, pairwise analyses in the current review. This was due to limited data and few intervention nodes to allow additional, indirect, comparisons to be formed (see Measures of treatment effect and Effects of interventions for more information). Instead, we considered the extracted information regarding these risk modifiers as subgroups within each comparison (see Subgroup analysis and investigation of heterogeneity).

#### Assessment of risk of bias in included studies

Two of the review authors (VNG, RC, LJG, SJB) independently assessed the risk of bias within each trial and assigned it a classification of low, high or unclear risk (Higgins 2011a; Higgins 2011b). We resolved disagreements through discussion.

We assessed risk of bias in the following domains:

- selection bias (random sequence generation and allocation concealment);
- performance bias (blinding of participants and personnel);
- detection bias (blinding of outcome assessment);
- attrition bias (incomplete outcome data);
- reporting bias (selective reporting); and
- other forms of bias.

#### Measures of treatment effect

We planned to combine data in an NMA using Stata (frequentist approach (Stata 2017)), however, when designing the potential networks for the NMA, we noted that very few data contributed enough to each outcome to provide indirect comparisons (see Effects of interventions for further information).  We thus took the decision to perform only direct pairwise analyses using Review Manager 5 (Review Manager 2020). The full (original) protocol for this review, including the NMA, is available from Gibbs 2019a, and the NMA processes that may be used in future review updates are detailed in Appendix 1. 

When extracting data for dichotomous outcomes (proportion of participants who received an allogeneic blood transfusion, mortality, reoperation due to bleeding, adverse events), we recorded the number of participants and events in both the intervention and control arms.  

As we have only performed direct pairwise analyses, we have presented analyses using risk ratio (RR), risk difference (RD) where there were zero cases in both arms, or Peto odds ratio (Peto OR) for rare events (< 5% in each arm), always with 95% confidence intervals (CIs).

We extracted arm‐level data for continuous outcomes (e.g. mean number of allogeneic blood transfusions per participant), we recorded means, standard deviations (SD) (or medians with interquartile ranges (IQR)) and the total number of participants in both the intervention and control arms. Where only study‐level data were available, we noted the reported effect size and standard errors. 

None of the included studies reported our continuous outcomes in an analysable format (reported as median IQR/range). For future updates, we will analyse continuous outcome data measured using the same scale using mean difference (MD) with a 95% CI. However, if this outcome is measured using different scales, we will use standardised mean difference (SMD) with 95% CI.

In future updates, if there are sufficient data to undertake an NMA, we will use Stata to do the quantitative analyses (frequentist approach) (see Gibbs 2019a and Appendix 1 for more detail regarding the NMA methods).

#### Unit of analysis issues

For trials with multiple treatment groups or interventions, we included subgroups that we considered relevant to the analysis. If appropriate, we combined groups to create a single pair‐wise comparison. If this was not possible, we selected the most appropriate pair of interventions and excluded the others (Higgins 2022b). We analysed the data using the participant as the unit of analysis. No trials randomised participants more than once. 

Where studies reported multiple time points, we carefully read the data, and used the total of individuals experiencing an event up to our defined time point. Where it was not clear if the number of events were being reported, instead of the number of individuals (e.g. an individual had multiple events), we contacted the trial authors for further clarification, and did not use the data where double‐counting may have occurred.

However, in future updates, this will not be the case in the NMA where we will include all comparisons, if and when there are adequate data to do so. We will analyse these trials by taking into account the respective treatment effects. The NMA method correctly accounts for correlations in relative effects from trials with more than two arms. We will analyse data with the participant as the unit of analysis.

In future updates, in the event that we include one or more cluster‐RCTs, we will follow the guidance in Chapter 23 of the *Cochrane Handbook for Systematic Reviews of Interventions* (Higgins 2022b), using a method of generic inverse variance in RevMan. We will also carefully consider the potential risk of bias associated with the method of randomisation described.

#### Dealing with missing data

We did not identify any missing data from the included studies. If we had identified data as being missing or unclear in the published literature, we would have contacted trial authors directly. In such an instance, if we were still unable to obtain the information, and the missing data were thought to lead to serious bias, we would perform a sensitivity analysis to assess the impact of the missing outcome data. 

We recorded the number of participants lost to follow‐up for each trial. Where possible, we analysed data on an intention‐to‐treat (ITT) basis, but if insufficient data were available, we also presented a per protocol analysis. We handled missing data using the approach discussed in Chapter 10 of the *Cochrane Handbook for Systematic Reviewsof Interventions* (Deeks 2022).

#### Assessment of heterogeneity

##### Assessment of clinical and methodological heterogeneity within treatment comparisons

For pair‐wise meta‐analyses, we assessed statistical heterogeneity of treatment effects between trials using a Chi² test with a significance level at P < 0.1. We used the I² statistic to measure the percentage of total variability due to between‐study heterogeneity and classified it as moderate if the I² statistic was greater than 50%, or considerable if the I² statistic was greater than 75% (Higgins 2003). We used the random‐effects model as we anticipated that we would identify at least moderate clinical and methodological heterogeneity within the trials selected for inclusion. If statistical heterogeneity was considerable, we did not report the overall summary statistic. We assessed potential causes of heterogeneity by sensitivity and subgroup analyses (Deeks 2022).

See Gibbs 2019a and Appendix 1 for more detail regarding the NMA methods to be used in future updates.

#### Assessment of reporting biases

No meta‐analysis in this review included at least 10 trials, therefore we could not perform a formal assessment of publication bias (Page 2022).

In future updates, we will investigate the presence of small‐study effects in the pair‐wise meta‐analyses through funnel plots and linear regression, if there are at least 10 studies. We will use a threshold of 0.10 or below for a P value to be statistically significant. Several factors can contribute to the association between study effect size and funnel plot asymmetry. We will differentiate between funnel plot asymmetry caused by publication bias using contour‐enhanced funnel plots (Peters 2008). The contour lines in the plot demonstrate levels of statistical significance. We will assume that a lack of studies in areas of non‐significance will show signs of publication bias.

#### Data synthesis

For pair‐wise meta‐analyses, we performed direct treatment comparisons using methods described in Chapter 10 of the *Cochrane Handbook for Systematic Reviews of Interventions* (Deeks 2022). Where data were homogeneous enough to do so, we performed meta‐analyses in Review Manager 5 (Review Manager 2020). Forest plots illustrating these results are shown with 95% CIs for all analyses, using the random‐effects model (as described in Assessment of heterogeneity). 

See Gibbs 2019a and Appendix 1 for more detail regarding the NMA methods to be used in future updates.

#### Subgroup analysis and investigation of heterogeneity

##### Subgroup analysis

There were insufficient data to perform all the planned subgroup analyses. In future updates, if the data allow, we will perform subgroup analyses and network meta‐regression for the following variables, to explain any heterogeneity, inconsistency, or both, across all outcomes:

- type of surgery;
- participants with preoperative anaemia;
- participants on anticoagulant or antiplatelet therapy at the time of injury.

See Data extraction and management for more information.

However, we were able to subgroup by the type of injury and the resultant surgery:

- hip arthroplasty;
- hip fixation;
- mixed population;
- other: including femoral shaft fixation and pelvic surgery.

##### Investigation of heterogeneity

While performing pair‐wise meta‐analyses, we evaluated heterogeneity in each pair‐wise comparison using the I² statistic, as described in Assessment of heterogeneity. 

See Gibbs 2019a and Appendix 1 for more detail regarding the NMA methods to be used in future updates.

#### Sensitivity analysis

Using the information generated, we looked for statistical heterogeneity in each trial and planned to perform sensitivity analyses accordingly. We planned to do this for the primary outcomes in the first instance, and then apply this to other outcomes with significant heterogeneity. However, we did not perform any sensitivity analyses due to the low heterogeneity between studies, and lack of data.

In future updates, we will examine the strength of the overall results by performing sensitivity analyses, where appropriate, with and without the trials thought to be at high risk of bias. 

In future updates where sensitivity analyses are necessary due to heterogeneity between studies, and where there are sufficient data, we will perform our main analyses using studies deemed at low risk of bias, and then undertake a sensitivity analysis, which incorporates all the included studies. We will look at the effect of participant dropout, and will categorise the trials into groupings of:

- less than 20% dropout;
- 20% to 50% dropout and
- more than 50% dropout.

We will analyse each group separately. We will explore heterogeneity using a fixed‐effect model to assess sensitivity.

#### Summary of findings and assessment of the certainty of the evidence

We assessed certainty of evidence using GRADEpro GDT and exported our assessment of the evidence into Summary of Findings tables. 

##### Summary of findings table

We used the GRADE approach to generate a summary of findings table as suggested in the *Cochrane Handbook for Systematic Reviews of Interventions* (Schünemann 2022). We produced summary of findings tables where more than one study contributed data to a comparison. We used the GRADE approach to rate the certainty of the evidence as 'high', 'moderate', 'low', or 'very low' using the five GRADE considerations.

- Risk of bias (serious or very serious)
- Inconsistency (serious or very serious)
- Indirectness (serious or very serious)
- Imprecision (serious, very serious, or extremely serious)
- Publication bias (suspected or undetected)

See Gibbs 2019a and Appendix 1 for more detail regarding the NMA methods to be used in future updates.

Cochrane summary of findings tables are restricted to just seven outcomes. We have therefore only presented data in the summary of findings tables for the following outcomes (from the 10 listed in the Primary outcomes and Secondary outcomes):

- risk of requiring allogeneic blood transfusion(30 days);
- all‐cause mortality (30 days);
- risk of re‐operation for bleeding (7 days);
- risk of myocardial infarctions (30 days);
- risk of cerebrovascular accidents/strokes (30 days);
- risk of deep vein thromboses (30 days); and
- risk of serious suspected drug reaction (30 days). 

We have selected the most clinically important outcomes for inclusion within the summary of findings tables. The number of participants who receive red blood cell transfusions is more important than the number of red blood cells per participant, as avoidance of red blood cell transfusion is more important to individuals than reducing the number of red blood cell units transfused. Venous thromboembolism (pulmonary embolism or deep vein thrombosis) is an important outcome for this patient group. Deep vein thromboses occur more commonly than pulmonary embolisms and therefore any potential harm will be detected with a smaller number of participants. Adverse drug reactions are more important than transfusion reactions because it is important to know whether a treatment that reduces the risk of a transfusion has a high risk of serious adverse events.

We have reported all analyses for all 10 outcomes in the Data and analyses and Effects of interventions.

## Results

### Description of studies

See also Characteristics of included studies; Characteristics of excluded studies; Characteristics of studies awaiting classification; Characteristics of ongoing studies

#### Results of the search

See PRISMA flow diagram (Figure 1).

**1:**
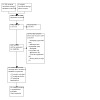
Study flow diagram

We identified 11,228 references, and we removed 3447 as duplicates. We screened 7781 references at title and abstract level, and 268 at full‐text level. We excluded 162 full‐text articles (see Excluded studies; Characteristics of excluded studies for more information). We therefore included 106 records as 77 independent trials: 13 published peer‐reviewed studies (929 participants), 27 marked as ongoing (yet to be published), and 37 awaiting classification (waiting to hear from the authors about the trial registration details, or further detail from the translations).

We included 10 studies (728 participants) in the quantitative analyses, as three of the published (included) studies did not provide usable data for our outcomes (Kashefi 2012; Monsef Kasmaei 2019; NCT01727843).

#### Included studies

An overview of characteristics for all included studies by comparison can be seen in Table 4, Table 5, and Table 6.

**2:** Overview of included studies in comparison 1: intravenous tranexamic acid versus placebo

**Study**	**Participants ** **(inclusion criteria)**	**Intervention**	**Comparator **	**Outcomes**
**Subgroup: hip fixation**				
Haghighi 2017 Single‐centre Iran N = 38	20‐50 years (ASA grade I‐II) Femoral fracture with intramedullary nailing	TXA, IV, 15 mg/kg, pre‐op Mean age: 65 years 14 M, 4 F	Placebo, IV, 15 mg/kg, pre‐op Mean age: 66 years 17 M, 3 F	Transfusions^a^ (hospital stay, to discharge)
Lei 2017 Single‐centre China N = 77`	Intertrochanteric fracture	TXA, IV, 1 g /200 mL, pre‐op Mean age: 78 years 32 F, 5 M	Placebo (saline), IV, 200 mL, pre‐op Mean age: 79 years 33 F, 7 M	Transfusions (3 days) Mortality (30 days) RBC units^b^ transfused reported per group, not per participant MI (30 days) CVA/stroke (30 days) DVT (30 days) PE (30 days)
Luo 2019 Multi‐centre China N = 90	60+ years Intertrochanteric fracture treated with PFNA, or closed fracture with low‐energy damage	TXA, IV, 15 mg/kg (body weight), pre‐op, and 3 h later, repeated dose Mean age: 75 years 23 M, 21 F	Placebo (saline), IV, 100 mL, pre‐op Mean age: 76 years 20 M, 26 F	Transfusions (3 days) Mortality (6 weeks) CVA/stroke (6 weeks) DVT (6 weeks)
Ma 2021 Single‐centre China N = 125	65+ years First fresh unilateral femoral intertrochanteric fracture (within 6 h)	IV TXA: 1 g (200 mL) post‐admission (pre‐op) Mean age: 78 years 42 F, 21 M	IV saline (200 mL) post‐admission (pre‐op) Mean age: 79 years 40 F, 22 M	CVA/stroke DVT PE Serious drug reaction Follow‐up to 90 days, but all outcomes had zero events so we can infer zero at all earlier time points
Zhang 2020a Single‐centre China N = 122	18+ years Hip fracture surgery for isolated intertrochanteric fracture treated with PFNA	TXA, IV, 1 g in 100 mL, 10 min pre‐incision (intra‐op) and post‐op Mean age: 79 years 28 M, 33 F	Placebo (saline), IV, 100 mL, 10 min pre‐incision (intra‐op) and post‐op Mean age: 76 years 34 M, 27 F	Transfusions (to discharge Mortality (90 days) MI (90 days, but zero events so we infer at 30 days) CVA/stroke (90 days, but zero events so we infer at 30 days) DVT (90 days) PE (90 days) Complications were reported at 90 days only
**Subgroup: mixed**				
Parish 2021 Single‐centre Iran N = 60	18+ years T Type, transverse and associated acetabular fracture (femoral fracture surgery with concher insertion)	TXA IV, 10 mg/kg 15 min before infusion, then infusion at 1 mg/kg/h until end of surgery (intra‐op) Mean age: 44 years 8 F, 22 M	NS (10 mg/kg) 15 min before infusion (intra‐op) Mean age: 47 years 7 F, 23 M	Transfusions (48 h) RBC units (up to 48 h) DVT (3 weeks) PE (reported as "zero thromboembolic events"; 3 weeks) Serious drug reaction (reported as "no complications of TXA injection"; 3 weeks)
Sadeghi 2007 Single‐centre Iran N = 67	People with hip fractures with extracapsular fractures treated by plating and nailing, and intracapsular fractures, treated by hemiarthroplasty	TXA, IV, 15 mg/kg, pre‐op (at anaesthesia) Mean age: 52 years 17 M, 15 F	Placebo (saline), IV, 15 mg/kg, pre‐op (at anaesthesia) Mean age: 44 years 24 M, 11 F	Mortality (7 days) Transfusions (during or after the operation, to discharge) RBC units per participant (to discharge) DVT, reported as "no thromboembolic complications" (6 weeks; but zero cases so we can infer at earlier time points) PE, reported as "no thromboembolic complications" (6 weeks; but zero cases so we can infer at earlier time points)
**Subgroup: other **				
Kashefi 2012 Setting: not reported Iran N = 80	18‐64 years Femoral trunk/shaft surgery	TXA, IV, 15 mg/kg (5 mL), pre‐op Mean age: 43 years 31 M, 9 F	Placebo (saline), IV, 5 mL of liquid (15 mg/kg), pre‐op Mean age: 40 years 33 M, 7 F	No usable data (translation unclear for group allocation and baseline data); follow‐up time not reported in translation provided
Monsef Kasmaei 2019 Single‐centre Iran N = 106	18‐60 years Pelvic trauma (within 3 h)	TXA, IV, 1 g, loading dose time point not reported, repeated dose time point not reported Age: not reported 36 M, 17 F	Placebo, IV, 0.9%, time point not reported Age: not reported 29 M, 24 F	No relevant outcomes
**CVA:** cerebrovascular accident; **DVT:** deep vein thrombosis; **F:** female; **IV:** intravenous; **M:** male; **MI:** myocardial infarction; **NS:** normal saline; **PE:** pulmonary embolism; **PFNA:** proximal femoral nail anti‐rotation; **RBC:** red blood cell; **TXA:** tranexamic acid				

^a^'Transfusions' relates to the reporting of the proportion of participants who required allogeneic blood transfusion.  ^b^'RBC units' (red blood cell units) relates to the reporting of the volume of blood transfused.

**3:** Overview of included studies in comparison 2: topical tranexamic acid versus placebo

**Study**	**Participants ** **(inclusion criteria)**	**Intervention**	**Comparator **	**Outcomes**
**Subgroup: Hip arthroplasty**				
NCT02664909** ** **2021** Single‐centre USA N = 36	55+ years Hip hemiarthroplasty surgery for a displaced femoral neck fracture	1 g TXA (topical) into surgical wound, at wound closure (intra‐op) Mean age: 83 years 14 F, 3 M	50 mL saline (topical) into surgical wound, at wound closure (intra‐op) Mean age: 83 years 17 F, 2 M	Transfusions^a^ (to discharge, 2‐4 days post‐op) Mortality (6 weeks, but zero cases so we can infer at earlier time points) RBC^b^ units (to discharge) MI (4‐6 weeks) DVT, as venous thrombosis (4‐6 weeks)
**Subgroup: mixed**				
Costain 2021 Single‐centre Canada N = 65	18+ years Hip fracture: intracapsular, intratrochanteric or subtrochanteric	3 g TXA, topical, intra‐op Mean age: 80 years 20 F, 11 M	50 mL saline; topical, intra‐op Mean age: 79 years 25 F, 9 M	Transfusions (3 days) Mortality (90 days) RBC units (3 days) CVA/stroke (30 days) DVT (30 days)
NCT01727843** ** 2018 Single‐centre Canada N = 15	65+ years Hip fracture	3 g TXA, topical, end of surgery (intra‐op) Age: not reported Gender: not reported	3 g saline; topical, end of surgery (intra‐op) Age: not reported Gender: not reported	No data available (terminated prematurely)
**CVA:** cerebrovascular accident; **DVT:** deep vein thrombosis; **F:** female; **M:** male; **MI:** myocardial infarction; **NS:** normal saline; **PE:** pulmonary embolism; **PFNA:** proximal femoral nail anti‐rotation; **RBC:** red blood cell; **TXA:** tranexamic acid				

^a^'Transfusions' relates to the reporting of the proportion of participants who required allogeneic blood transfusion. ^b^'RBC units' (red blood cell units) relates to the reporting of the volume of blood transfused.

**4:** Overview of included studies in comparison 3: rFVIIa versus placebo

**Study**	**Participants ** **(inclusion criteria)**	**Intervention**	**Comparator **	**Outcomes**
**Subgroup: other **				
Raobaikady 2005 Single‐centre UK N = 48	18‐60 years Major pelvic–acetabular fracture caused by trauma, requiring “large” reconstruction	rfVIIa, IV, 90 µg/kg, intra‐op Age: median 44 years 16 M, 8 F	Placebo, IV, 90 µg/kg, intra‐op Age: median 38 years 18 M, 6 F	Transfusions^a^ (perioperative, up to 48 h post‐op) RBC^b^ units (48 h) Re‐operation (48 h) DVT, reported as "zero thromboembolic events" (30 days) PE, reported as "zero thromboembolic events" (30 days) Serious drug reaction, reported as zero events "related to rFVIIa" (30 days)
**CVA:** cerebrovascular accident; **DVT:** deep vein thrombosis; **F:** female; **IV:** intravenous; **M:** male; **MI:** myocardial infarction; **NS:** normal saline; **PE:** pulmonary embolism; **PFNA:** proximal femoral nail anti‐rotation; **RBC:** red blood cell; **rFVIIa:** recombinant factor VIIa; **TXA:** tranexamic acid				

^a^'Transfusions' relates to the reporting of the proportion of participants who required allogeneic blood transfusion. ^b^'RBC units' (red blood cell units) relates to the reporting of the volume of blood transfused.

##### Study selection

Thirteen RCTs met the predefined inclusion criteria (Costain 2021;Haghighi 2017;Kashefi 2012; Lei 2017; Luo 2019; Ma 2021; Monsef Kasmaei 2019; NCT01727843; NCT02664909; Parish 2021; Raobaikady 2005; Sadeghi 2007; Zhang 2020a).

Three trials did not report any of our predefined outcomes of interest (Kashefi 2012; Monsef Kasmaei 2019; NCT01727843), though one may be due to limitations from translation (Kashefi 2012), and so were not included in the analyses. Two compared intravenous tranexamic acid to placebo, and one compared topical tranexamic acid to placebo but was terminated prematurely (NCT01727843).

##### Trial design

Most of the included trials were single‐centre trials (Costain 2021; Haghighi 2017; Lei 2017; Ma 2021; Monsef Kasmaei 2019; NCT01727843; NCT02664909; Parish 2021; Raobaikady 2005; Sadeghi 2007; Zhang 2020a). Only two were not: one multi‐centre trial (Luo 2019), and one where it was not clear, possibly due to translation issues (Kashefi 2012).

Follow‐up post‐surgery ranged from 24 hours (Haghighi 2017), 48 hours (Parish 2021), and 72 hours (Monsef Kasmaei 2019), to three months (Zhang 2020a). Most studies reported follow‐up for four to six weeks (Costain 2021; Lei 2017; Luo 2019; NCT02664909; Raobaikady 2005; Sadeghi 2007).

##### Trial size

The number of participants enroled in the trials ranged from 36 (NCT02664909) to 125 (Ma 2021); only three trials enroled more than 100 participants (Ma 2021; Monsef Kasmaei 2019; Zhang 2020a). One trial only recruited 15 participants and terminated prematurely, with no data available for our analyses (NCT01727843).

Nine studies reported power calculations or minimum sample size (Costain 2021; Haghighi 2017; Lei 2017; Luo 2019; Ma 2021; NCT02664909; Parish 2021; Raobaikady 2005; Zhang 2020a), however, of those nine, three did not recruit and analyse their required sample size (Haghighi 2017; Luo 2019; NCT02664909), one only met the sample size for some outcomes (Costain 2021), and one was not clear (Parish 2021).

Four studies did not report a power calculation (Kashefi 2012; Monsef Kasmaei 2019; NCT01727843; Sadeghi 2007), though for two studies this may be due to a translation issue (Kashefi 2012; Zheng 2020). 

##### Setting

The included trials were published between 2005 and 2021. Five were conducted in Iran (Haghighi 2017; Kashefi 2012; Monsef Kasmaei 2019; Parish 2021; Sadeghi 2007), four in China (Lei 2017; Luo 2019; Ma 2021; Zhang 2020a), two in Canada (Costain 2021; NCT01727843), one in the USA (NCT02664909), and one in the UK (Raobaikady 2005).

##### Participants

Trial participants varied in age, largely due to variations in inclusion criteria: four were specifically in the elderly (specifically over 55 to 65 years: Luo 2019; Ma 2021; NCT01727843; NCT02664909), three did not specify that participants should be older, but had an average age of over 60 years (Costain 2021; Lei 2017; Zhang 2020a). One trial specified participants aged 20 to 50 years in their inclusion criteria, but had a mean age of approximately 65 years in their final analysed cohort (Haghighi 2017).

Four studies assessed middle‐aged adults (average 38 to 52 years old: Kashefi 2012; Parish 2021; Raobaikady 2005; Sadeghi 2007). 

One trial did not report age in their inclusion criteria, or baseline characteristics (Monsef Kasmaei 2019).

All studies included both men and women, and within‐study gender distribution was well balanced between groups (no baseline imbalances). Four studies had significantly more women than men (Costain 2021; Lei 2017; Ma 2021; NCT02664909), five had significantly more men (Haghighi 2017; Kashefi 2012; Monsef Kasmaei 2019; Parish 2021; Raobaikady 2005), and three were approximately equal (Luo 2019; Sadeghi 2007; Zhang 2020a). One did not provide any baseline data (NCT01727843).

Hip fixation surgery was the most commonly used procedure, and this was the only procedure assessed by five trials (Haghighi 2017; Lei 2017; Luo 2019; Ma 2021; Zhang 2020a). One trial reported exclusively on hip arthroplasty procedures (NCT02664909), four trials utilised a mixed population (various fractures of the hip, femur, and pelvis; Costain 2021; NCT01727843; Parish 2021; Sadeghi 2007), and the remaining three trials were classified as 'other' fractures/surgeries (femoral shaft fixation: Kashefi 2012; pelvic trauma: Monsef Kasmaei 2019; pelvic surgery: Raobaikady 2005).

Most of the trials that assessed 'older' participants focused exclusively on hip fixation and hip arthroplasty procedures. Only one that terminated prematurely and provided no data did not (NCT01727843).

##### Interventions

In this review, we report the Effects of interventions by the various comparisons in the different trials. Most trials assessed tranexamic acid administered in various ways (intravenous or topical). Only one trial assessed a non‐tranexamic acid pharmaceutical, recombinant factor VIIa (Raobaikady 2005).

The comparisons, subgroups, and trials included the following.

- **Intravenous tranexamic acid versus placebo** (Table 4): 
  - hip fixation (5 trials, 452 participants; Haghighi 2017; Lei 2017; Luo 2019; Ma 2021; Zhang 2020a);
  - mixed (2 trials, 127 participants; Parish 2021; Sadeghi 2007); and
  - other (2 trials, 186 participants; femoral trunk: Kashefi 2012; pelvic trauma: Monsef Kasmaei 2019).
- **Topical tranexamic acid versus placebo** (Table 5): 
  - hip arthroplasty (1 trial, 36 participants; NCT02664909); and
  - mixed (2 trials, 80 participants; Costain 2021; NCT01727843).
- **Recombinant factor VIIa versus placebo** (Table 6):** ** 
  - other (1 trial, 48 participants; pelvic surgery: Raobaikady 2005).

##### Outcomes

The following trials reported our primary outcomes.

- Risk of requiring an allogeneic blood transfusion up to 30 days; (9 trials; Costain 2021; Haghighi 2017; Lei 2017; Luo 2019; NCT02664909; Parish 2021; Raobaikady 2005; Sadeghi 2007; Zhang 2020a)
- All‐cause mortality up to 30 days; (3 trials; Lei 2017; NCT02664909; Sadeghi 2007).

The following outcomes were most commonly reported by the included trials.

- Risk of requiring allogeneic blood transfusion (9 trials: as listed above)
- Risk of deep vein thrombosis (7 trials; Costain 2021; Lei 2017; Ma 2021; NCT02664909; Parish 2021; Raobaikady 2005; Sadeghi 2007). 

Trials also reported other adverse events we had listed, including:

- risk of pulmonary embolism (5 trials; Lei 2017; Ma 2021; Parish 2021; Raobaikady 2005; Sadeghi 2007);
- cerebrovascular accident/stroke (3 trials; Costain 2021; Lei 2017; Zhang 2020a);
- myocardial infarction (3 trials; Lei 2017; NCT02664909; Zhang 2020a);
- serious drug reaction (3 trials; Ma 2021; Parish 2021; Raobaikady 2005); and
- reoperation for bleeding (up to 7 days); (1 trial; Raobaikady 2005).

No trials reported usable data for the mean number of red blood cell units transfused per person (or another volume of measurement), though some reported in another form (Costain 2021; Lei 2017; NCT02664909; Parish 2021; Raobaikady 2005; Sadeghi 2007), and we have presented these raw data in Table 7.  No trials reported acute transfusion reactions (within 24 hours).

**5:** Additional data (not included in analyses)

**Study**	**Intervention data**	**Comparator data**	**Timing (reason for not being included in the analysis)**
**Comparison 1: intravenous tranexamic acid versus placebo**			
**Mortality (n/N)**			
Luo 2019	0/44	1/46	6 weeks (beyond 30 days)
Zhang 2020a	1/61	2/61	90 days (beyond 30 days)
**RBC^a^ units transfused (N** = **number of people transfused)**			
Parish 2021	Mean 0; SD 0; N = 0	Mean 2.25; SD 0.774507; N = 16	48 h (1 arm has N = 0; no transfusions^b^)
Sadeghi 2007	Mean 1.25; no SD, no N	Mean 1.95; no SD, no N	During or after the operation, to discharge (no SD reported, unclear if the mean is based on number transfused or number randomised)
**CVA/stroke (n/N)**			
Luo 2019	0/44	3/46	6 weeks (beyond 30 days)
**DVT (n/N)**			
Luo 2019	1/44	1/46	6 weeks (beyond 30 days)
Zhang 2020a	2/61	1/61	90 days (beyond 30 days)
**PE (n/N)**			
Zhang 2020a	1/61	0/61	90 days (beyond 30 days)
**Comparison 2: topical tranexamic acid versus placebo**			
**Mortality (n/N)**			
Costain 2021	2/31	1/34	90 days (beyond 30 days)
**RBC units transfused (N** = **number of people transfused)**			
NCT02664909	Mean 0; SD 0; N = 0	Mean 1.2; SD 0.45; N = 5	To discharge (1 arm has N = 0; no transfusions)
Costain 2021	Mean 1; SD 0; N = 2	Mean 1.6; SD 0.894427; N = 5	3 days (N very small in both arms)
**Comparison 3: intravenous recombinant factor VIIa versus placebo**			
**RBC units transfused (N** = **number of people transfused)**			
Raobaikady 2005	Median 0; range 0‐4; N = 24	Median 2; range 0‐16; N = 24	Perioperative period, up to 48 h post‐op (median and range only)
**CVA:** cerebrovascular accident; **DVT:** deep vein thrombosis; n: number of people experiencing the event; N: number of people in analysis; **RBC:** red blood cells; **SD:** standard deviation			

^a^'RBC units' (red blood cell units) relates to the reporting of the volume of blood transfused. ^b^'Transfusions' relates to the reporting of the proportion of participants who required allogeneic blood transfusion.

The included trials mostly did not use the same primary outcomes as we have for this review. Their primary outcomes were:

- blood loss or bleeding (intra‐operatively, post‐operatively, or peri‐operatively); (8 trials; Kashefi 2012; Lei 2017; Luo 2019; Monsef Kasmaei 2019; Parish 2021; Raobaikady 2005; Sadeghi 2007; Zhang 2020a);
- haemoglobin and/or haematocrit level or change (8 trials Costain 2021; Haghighi 2017; Ma 2021; Monsef Kasmaei 2019; NCT02664909; Parish 2021; Sadeghi 2007; Zhang 2020a);
- number of people who received a blood transfusion (5 trials; Kashefi 2012; Luo 2019; NCT02664909; Parish 2021; Zhang 2020a); and
- Deep vein thrombosis or thrombotic events (2 trials; Luo 2019; Parish 2021).

##### Timing of outcomes and follow‐up

We were unable to analyse some data as the only reporting was outside our defined period of 30 days. This occurred for mortality (reporting up to 6 weeks: Luo 2019, and 90 days: Costain 2021; Zhang 2020a), and some thromboembolic events (cerebrovascular accident/stroke: Luo 2019; deep vein thrombosis: Luo 2019; Zhang 2020a; pulmonary embolism: Zhang 2020a). We have extracted and tabulated this information, and present it in Table 7.

Where trials recorded beyond 30 days, but zero cases were reported, we were able to include the data by inferring that zero cases at their reported time point was also zero cases at any earlier time point (mortality: Ma 2021; NCT02664909; myocardial infarction: Ma 2021; Zhang 2020a; cerebrovascular accident/stroke: Ma 2021; Zhang 2020a; deep vein thrombosis: Ma 2021; Sadeghi 2007; pulmonary embolism: Sadeghi 2007; serious drug reaction: Ma 2021).

##### Sources of support

Nine trials were supported through funding from non‐pharmaceutical sources (state funding, universities, hospitals: Costain 2021; Haghighi 2017; Lei 2017; Ma 2021; Monsef Kasmaei 2019; NCT02664909; NCT01727843; Parish 2021; Zhang 2020a).

One trial was supported by a pharmaceutical company (Novo Nordisk, UK: Raobaikady 2005), one reported receiving no funding (Luo 2019), and one did not report sponsorship (Sadeghi 2007).

One trial could not be assessed regarding sources of support due to translation limitations (Kashefi 2012).

#### Excluded studies

We excluded 142 trials.

- Ineligible population (e.g. elective or scheduled surgery, non‐trauma; 59 trials; ACTRN 12613000323729; ACTRN 12613001043729; Alipour 2013; Antinolfi 2010; Arslan 2018; Cao 2015; Barrachina 2016; Benoni 2001; Bidolegui 2014; Borisov 2011; Bradley 2019; Camarasa 2006; Cankaya 2017; Cao 2018; Cao 2019; Castro‐Menendez 2016; Cerciello 2014; Chen 2018; Chin 2020; Clave 2019; Colwell 2007; Cvetanovich 2018; D'Ambrosio 1998; Ekback 2000; Fischer 2013; Fleischmann 2011; Flordal 1991; Fraval 2017; Fraval 2018; Garcia‐Enguita 1998; Gillespie 2015; Gomez Barbero 2019; Gulabi 2019; Ivie 2016; Jans 2016; Jaszczyk 2015; Koea 2015; Lei 2018; Llau 1998; Na 2016; NCT00658723; NCT01199627; NCT02233101; NCT02569658; NCT02584725; North 2016; Petsatodis 2006; Qiu 2019; Samama 2002; Tulaja Prasad 2021; Vara 2017; Vles 2020; Wang 2016; Wang 2019; Wei 2014; Wendt 1982; Xie 2016; Yamasaki 2004; Zhao 2016)
- Retrospective trial registration, where the trial was first registered after recruitment had started (55 trials; ChiCTR 1800019266; ChiCTR 1900027435; ChiCTR 2000032102; ChiCTR 2000032836; ChiCTR 2000033135; ChiCTR 2000034882; ChiCTR‐IDR‐17010966; ChiCTR‐TRC‐14004379; IRCT 201111198131N; IRCT 2013100414302N; IRCT 2016061328437N; IRCT 2017050126328N; IRCT 20180404039188N2; IRCT 20180422039382N; IRCT 20200114046133N1; IRCT 20211208053326N1; ISRCTN 02543733; ISRCTN 55488814; ISRCTN 58762744; ISRCTN 59245192; Jordan 2016; Jordan 2019; Lack 2017; Najafi 2014; Narkbunnam 2021; NCT01535781; NCT01714336; NCT01866943;  NCT02043132; NCT02051686; NCT02080494; NCT02150720; NCT02252497; NCT02580227; NCT02684851; NCT02747615; NCT02947529; NCT03019198; NCT03251469; NCT03653429; NCT03825939; NCT04488367; NCT04696224; NCT04986813; NCT05047133; Nikolaou 2021; Saravanan 2020; TCTR 20201224005; TCTR 202102090010; TCTR 20220104001; Tengberg 2016; Van Elst 2013; Watts 2017; Yee 2022; Zufferey 2010)
- Ineligible comparator (e.g. standard care); (9 trials; Ahmed 2010; Galué 2015; Huang 2021; Liu 2015; Luo 2012; NCT00824564; Ozay 1995; Rajesparan 2009; Ruiz‐Moyano 1997)
- Withdrawn prior to study starting (9 trials: ChiCTR 1800016634; Gausden 2016; NCT00375440; NCT01326403; NCT02164565; NCT02644473; NCT02908516; NCT03679481; NCT04803591
- Unregistered trial: author confirmed the trial was not registered at all (8 trials; Baruah 2016; Batibay 2018; Hourlier 2012; Mukherjee 2016; Schiavone 2018; Shodipo 2022; Thipparampall 2017; Zhou 2019)
- Ineligible study design (e.g. non‐RCT); (2 trials; Anonymous 2019 (various); Yu 2020)

#### Studies awaiting classification

Thirty‐seven studies are awaiting classification.

- Author unresponsive/could not confirm whether trial had been registered prospectively (25 studies; Akram 2021; Chen 2019; ChiCTR‐IPR‐17011260; Drakos 2016; Emara 2014; Li 2021; Lin 2021; Liu 2022; Luo 2018; Moghaddam 2009; Mohib 2015; NCT02738073; Sahni 2021; Singh 2020; Spitler 2019; Taheriazam 2015; Taheriazam 2016; Tian 2018; Vijay 2013; Wang 2021; Wu 2016; Yang 2020; Zhang 2019; Zhang 2020b; Zheng 2020)
- Unable to clarify if eligible patient population (12 studies; ChiCTR 1800015265; CTRI/2018/02/012030; Kazemi 2010; NCT01683955; NCT02094066; NCT02438566; NCT03157401; NCT03822793; NCT03897621; NCT04089865; NCT04187014; Notarfrancesco 2015)

#### Ongoing studies

Twenty‐seven studies are currently ongoing.

- Tranexamic acid versus placebo: 19 studies (ACTRN 12617000391370; ACTRN 12620001059954; ChiCTR 1800014309; ChiCTR 1800018334; ChiCTR 1900021948; ChiCTR 2000032758; ChiCTR‐ICC‐15006070; CTRI/2019/09/021302; CTRI/2021/09/036855; EUCTR 2018‐000528‐32; IRCT 2017 1030037093N18; Liu 2021; NCT02428868; NCT02972294 (HiFIT);  NCT03063892; NCT03182751; NCT03211286; NCT03923959; TCTR 2021 0311001)
- Tranexamic acid versus other tranexamic acid: six studies (ChiCTR 1800015809; ChiCTR‐IPR‐17013477; CTRI/2019/04/018735; CTRI/2019/10/021667; NCT02938962; TCTR 2021 0316006)
- Tranexamic acid versus non‐tranexamic acid: one study (EUCTR 2011‐006278‐15)
- Non‐tranexamic acid versus placebo: one study (IRCT 2020 0109046064N1)

### Risk of bias in included studies

Refer to risk of bias figures (Figure 2; Figure 3) for visual representations of the assessments of risk of bias across all trials and for each item in the included trials. See the risk of bias section in the Characteristics of included studies section for further information about the bias identified within individual trials.

**2:**
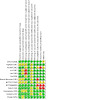
Methodological quality summary: review authors' risk of bias judgements about each methodological quality item for each included study.

**3:**
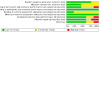
Methodological quality graph: review authors' judgements about each methodological quality item presented as percentages across all included studies

#### Allocation

##### Random sequence generation (selection bias)

We assessed three trials as unclear risk of bias (Haghighi 2017; Monsef Kasmaei 2019; NCT01727843).

We assessed the remaining 10 trials as low risk of bias.

##### Allocation concealment (selection bias)

We assessed seven trials as unclear risk of bias (Haghighi 2017; Lei 2017; Luo 2019; Monsef Kasmaei 2019; NCT01727843; Raobaikady 2005; Zhang 2020a).

The remaining six trials were low risk of bias (Costain 2021; Kashefi 2012; Ma 2021; NCT02664909; Parish 2021; Sadeghi 2007).

#### Blinding

For assessment of bias from blinding, we separately assessed the risk for objective and subjective outcomes. 

We considered objective outcomes to include mortality, and incidence of myocardial infarction, cerebrovascular accident or stroke, and pulmonary embolism due to the clear diagnostic criteria in wide use. 

We deemed the remaining outcomes to be subjective: risk of requiring an allogeneic blood transfusion, decision to re‐operate, incidence of serious drug reactions, and incidence of deep vein thrombosis due to the more subjective nature of a deep vein thrombosis diagnosis.

##### Blinding of participants and personnel (performance bias)

###### Subjective outcomes

We assessed four trials as unclear (Haghighi 2017; Kashefi 2012; Ma 2021; Raobaikady 2005), and two as high risk of bias (Lei 2017; Luo 2019).

We assessed six trials as low risk of bias (Costain 2021; Monsef Kasmaei 2019; NCT02664909; Parish 2021; Sadeghi 2007; Zhang 2020a). 

We assessed one trial as being of unclear risk of bias (NCT01727843). 

###### Objective outcomes

We assessed all 13 trials as low risk of bias. 

##### Blinding of outcome assessment (detection bias)

###### Subjective outcomes

We assessed 10 trials as having unclear risk of bias (Haghighi 2017; Kashefi 2012; Lei 2017; Luo 2019; Ma 2021; Monsef Kasmaei 2019; NCT01727843; Parish 2021; Raobaikady 2005; Sadeghi 2007).

We assessed the remaining three trials as being at low risk of bias (Costain 2021; NCT02664909; Zhang 2020a). 

###### Objective outcomes

We assessed all 13 trials as having low risk of bias.

#### Incomplete outcome data

We assessed three trials as unclear (Kashefi 2012; NCT01727843; Parish 2021), and two at high risk of bias (Monsef Kasmaei 2019; NCT02664909).

We assessed the remaining eight trials as low risk of bias (Costain 2021; Haghighi 2017; Lei 2017; Luo 2019; Ma 2021; Raobaikady 2005; Sadeghi 2007; Zhang 2020a).

#### Selective reporting

We assessed two trials as unclear (NCT01727843; Sadeghi 2007), and three at high risk of bias (Kashefi 2012; NCT02664909; Parish 2021).

We assessed the remaining eight trials as being at low risk of bias (Costain 2021; Haghighi 2017; Lei 2017; Luo 2019; Ma 2021; Monsef Kasmaei 2019; Raobaikady 2005; Zhang 2020a). 

#### Other potential sources of bias

Other biases that we considered (amongst others) included baseline imbalances, block randomisation in an unblinded trial, and funding and conflict reporting. We also noted where data were being drawn from a non‐peer‐reviewed publication, and any other potential risks. 

We assessed three as unclear (baseline imbalance: Costain 2021; lack of information on baseline characteristics: Kashefi 2012; no data presented: NCT01727843), and two with high risk of bias (lack of peer review: NCT02664909; baseline imbalance and changes to trial registration: Parish 2021). 

We assessed the remaining eight trials as being at low risk for other biases (Haghighi 2017; Lei 2017; Luo 2019; Ma 2021; Monsef Kasmaei 2019; Raobaikady 2005; Sadeghi 2007; Zhang 2020a). 

### Effects of interventions

See: Table 1; Table 2

#### Notes on analyses

##### Network meta‐analysis (NMA)

When designing the potential networks, we noted that very few data contributed enough to each outcome to provide indirect comparisons. The four‐node network of three interventions, centred around a placebo intervention, allowed three direct comparisons (as shown in the direct pairwise comparisons described here), and three additional indirect comparisons: recombinant factor VIIa (IV) versus two different methods of administering tranexamic acid (intravenous or topical), and comparison between intravenous or topical tranexamic acid, as depicted in Figure 4. Whilst this may be a useful comparison, two of the indirect comparisons would have been based on a single recombinant factor VIIa trial of only 60 people, with only one outcome (risk of requiring allogeneic blood transfusion) reported across all relevant comparisons.

**4:**
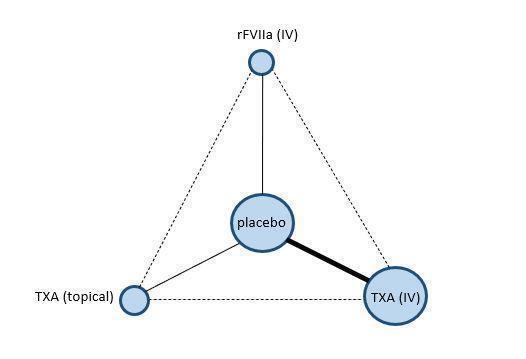
Four‐node network for included studies. Node size represents sample size, solid lines represent direct comparisons (thickness depicting more studies contributing to the comparison), and dashed lines depict potential indirect comparisons. This is an original image, created by one author (LJG)

We therefore concluded that performing an NMA of the available data would add very little value over the pairwise analyses we have presented here, and may lessen the certainty of the evidence due to the limited data available for a meta‐regression of potential risk modifiers. We have instead used these potential effect modifiers as subgroups within the direct pairwise meta‐analyses (type of surgery).  

##### Direct pair‐wise analyses

We identified three comparisons of interest. We have assessed the certainty of the evidence for all comparisons and outcomes using GRADE, though have presented summary of findings tables for only those comparisons where more than one trial contributed data (Table 1; Table 2). We did not formally analyse data from the single trial; we presented them as visual representations (forest plots) with subtotals only.

#### Comparison 1: intravenous tranexamic acid versus placebo

Seven RCTs investigated this comparison (Haghighi 2017; Lei 2017; Luo 2019; Ma 2021; Parish 2021; Sadeghi 2007; Zhang 2020a). See Table 4 for an overview of trial characteristics for this comparison and Table 1.

##### Risk of requiring allogeneic blood transfusion (30 days)

Intravenous tranexamic acid may reduce the risk of allogeneic blood transfusion up to 30 days (RR 0.48, 95% CI 0.34 to 0.69; 6 RCTs, 457 participants; low‐certainty evidence; Analysis 1.1).

##### All‐cause mortality (30 days post‐surgery)

Intravenous tranexamic acid may result in little to no difference in all‐cause mortality (Peto OR 0.38, 95% CI 0.05 to 2.77; 2 RCTs, 147 participants; low‐certainty evidence; Analysis 1.2).

##### Mean number of red blood cell units transfused per person (30 days) 

Two trials reported red blood cell units transfused (Parish 2021; Sadeghi 2007), but we were unable to analyse the data. We have presented these data, with the reason for exclusion from the analysis, in Table 7.

##### Re‐operation due to bleeding (7 days) 

No trials reported this outcome for this comparison.

##### Adverse events

###### Risk of participants experiencing myocardial infarction (30 days)

Intravenous tranexamic acid may result in little to no difference in risk of participants experiencing myocardial infarction (RD 0.00, 95% CI −0.03 to 0.03; 2 RCTs, 199 participants; low‐certainty evidence; Analysis 1.3).

###### Risk of participants experiencing cerebrovascular accident/stroke (30 days)

Intravenous tranexamic acid may result in little to no difference in risk of participants experiencing cerebrovascular accident/stroke (RD 0.00, 95% CI −0.02 to 0.02; 3 RCTs, 324 participants; low‐certainty evidence; Analysis 1.4).

###### Risk of participants experiencing deep vein thrombosis (30 days)

We are uncertain if there is a difference between groups in the risk of deep vein thrombosis (Peto OR 2.15; 95% CI 0.22 to 21.35; 4 RCTs, 329 participants; very low‐certainty evidence; Analysis 1.5).

###### Risk of participants experiencing pulmonary embolism (30 days)

We are uncertain if there is a difference between groups in the risk of pulmonary embolism (Peto OR 1.08, 95% CI 0.07 to 17.66; 4 RCTs, 329 participants; very low‐certainty evidence; Analysis 1.6).

###### Acute transfusion reaction (24 hours) 

No trials reported this outcome for this comparison.

###### Participants having suspected serious drug reactions (30 days)

We are uncertain if there is a difference between groups for the risk of serious drug reactions (RD 0.00; 95% CI −0.03 to 0.03; 2 RCTs, 185 participants; very low‐certainty evidence; Analysis 1.7)

We downgraded the certainty of the evidence for imprecision (wide confidence intervals around the estimate and small sample size, particularly for rare events), and risk of bias (unclear or high‐risk methods of blinding and allocation concealment in the assessment of subjective measures). 

#### Comparison 2: topical tranexamic acid versus placebo

Two RCTs reported this comparison (Costain 2021; NCT02664909). See Table 5 for an overview of trial characteristics and Table 2.

##### Risk of requiring allogeneic blood transfusion (30 days)

We are uncertain if there is a difference between groups for the risk of allogeneic blood transfusion (RR 0.31; 95% CI 0.08 to 1.22; 2 RCTs, 101 participants; very low‐certainty evidence; Analysis 2.1)

##### All‐cause mortality (30 days post‐surgery)

We are uncertain if there is a difference between groups for the risk of all‐cause mortality (RD 0.00, 95% CI −0.10 to 0.10; 1 RCT, 36 participants; very low‐certainty evidence; Analysis 2.2). This is a single‐study analysis only (NCT02664909).

##### Mean number of red blood cell units transfused per person (30 days) 

Two trials reported on red blood cell units transfused (Costain 2021; NCT02664909), but we were unable to analyse the data. We have presented these data, with the reason for exclusion from the analysis, in Table 7.

##### Re‐operation due to bleeding (7 days) 

No trials reported this outcome for this comparison.

##### Adverse events

###### Risk of participants experiencing myocardial infarction (30 days)

We are uncertain if there is a difference between groups for the risk of a myocardial infarction (Peto OR 0.15; 95% CI 0.00 to 7.62; 1 RCT, 36 participants; very low‐certainty evidence; Analysis 2.3). This is a single‐study analysis only (NCT02664909).

###### Risk of participants experiencing cerebrovascular accident/stroke (30 days)

We are uncertain if there is a difference between groups for the risk of a cerebrovascular accident (RD 0.00, 95% CI −0.06 to 0.06; 1 RCT, 65 participants; very low‐certainty evidence; Analysis 2.4). This is a single‐study analysis only (Costain 2021).

###### Risk of participants experiencing deep vein thrombosis (30 days)

We are uncertain if there is a difference between groups (Peto OR 1.11, 95% CI 0.07 to 17.77; 2 RCTs, 101 participants; very low‐certainty evidence; Analysis 2.5).

###### Risk of participants experiencing pulmonary embolism (30 days)

No trials reported this outcome for this comparison.

###### Acute transfusion reaction (24 hours) 

No trials reported this outcome for this comparison.

###### Participants having suspected serious drug reactions (30 days)

No trials reported this outcome for this comparison.

We downgraded the certainty of the evidence for imprecision (wide confidence intervals around the estimate and small sample size, particularly for rare events), inconsistency (moderate heterogeneity), and risk of bias (unclear or high‐risk methods of blinding and allocation concealment in the assessment of subjective measures, and high risk of attrition and reporting biases in one trial). 

#### Comparison 3: recombinant factor VIIa (recombinant factor VIIa) versus placebo

One RCT in pelvic surgery reported this comparison (Raobaikady 2005). See Table 6 for an overview of study characteristics.

We have not presented a summary of findings table as only one trial contributed to this comparison.

##### Risk of requiring allogeneic blood transfusion (30 days)

We are uncertain if there is a difference between groups in the risk of allogeneic blood transfusion (RR 0.69; 95% CI 0.41 to 1.16; 1 RCT, 48 participants; very low‐certainty evidence; Analysis 3.1).

##### All‐cause mortality (30 days post‐surgery)

No trials reported this outcome for this comparison.

##### Mean number of red blood cell units transfused per person (30 days) 

One trial reported red blood cell units transfused (Raobaikady 2005), but we were unable to analyse the data. We have presented these data, with the reason for exclusion from the analysis, in Table 7.

##### Re‐operation due to bleeding (7 days) 

We are uncertain if there is a difference between groups for the risk of reoperation due to bleeding (Peto OR 0.14; 95% CI 0.00 to 6.82; 1 RCT, 48 participants; very low‐certainty evidence; Analysis 3.2).

##### Adverse events

###### Risk of participants experiencing myocardial infarction (30 days)

No trials reported this outcome for this comparison.

###### Risk of participants experiencing cerebrovascular accident/stroke (30 days)

No trials reported this outcome for this comparison.

###### Risk of participants experiencing deep vein thrombosis (30 days)

We are uncertain if there is a difference between groups for the risk of deep vein thrombosis, with zero cases reported (RD 0.00, 95% CI −0.08 to 0.08; 1 RCT, 48 participants; very low‐certainty evidence; Analysis 3.3)

###### Risk of participants experiencing pulmonary embolism (30 days)

We are uncertain if there is a difference between groups in the risk of pulmonary embolism, with zero cases reported (RD 0.00, 95% CI −0.08 to 0.08; 1 RCT, 48 participants; very low‐certainty evidence; Analysis 3.4).

###### Acute transfusion reaction (24 hours) 

No trials reported this outcome for this comparison.

###### Participants having suspected serious drug reactions (30 days)

We are uncertain if there is a difference between groups for the risk of suspected serious drug reaction, with zero cases reported (RD 0.00, 95% CI −0.08 to 0.08; 1 RCT, 48 participants; very low‐certainty evidence; Analysis 3.5).

We downgraded the certainty of the evidence for imprecision (wide confidence intervals around the estimate and small sample size, particularly for rare events), and risk of bias (unclear or high‐risk methods of blinding and allocation concealment in the assessment of subjective measures). 

## Discussion

Pelvic, hip, and long bone fractures can result in significant bleeding at the time of injury, with further blood loss if surgical fixation is performed.

In this review we have examined the evidence for the use of pharmacological interventions to reduce bleeding in definitive surgical fixation of the hip, pelvic, and long bones.

Thirteen RCTs assessing a total of 929 participants met our inclusion criteria. Nine of the studies compared intravenous tranexamic acid to placebo (though two did not report any relevant outcomes in a usable form; Table 4); three compared topical tranexamic acid to placebo (one did not report any data; Table 5); and one study assessed recombinant factor V11a compared to placebo (Table 6). Trials were published between 2005 and 2021.

We also identified 27 prospectively registered ongoing RCTs (totalling 4177 participants if they recruit as planned), which should all complete by the end of 2023. The ongoing trials will contribute to the comparisons already established, and create six new comparisons:

- tranexamic acid (tablet + injection) versus placebo;
- intravenous tranexamic acid versus tranexamic acid (oral);
- topical tranexamic acid versus tranexamic acid (oral);
- intravenous tranexamic acid comparing different dosing regimes;
- topical tranexamic acid versus fibrin glue (topical); and
- fibrinogen (injection) versus placebo (Table 8; Table 9; Table 10; Table 11).

**6:** All studies (included and ongoing): tranexamic acid (any route) versus placebo

**Study**	**Participants (inclusion criteria)**	**Intervention**		**Comparator **	**Outcomes**
**TXA (IV)** vs **placebo**					
**Subgroup: hip arthroplasty**					
Liu 2021 (ongoing study) China N = 80 Expected start: 1 June 2021 Expected end: 1 Sept 2022	65+ years Hemi‐ or total hip arthroplasty (primary, unilateral, recent hip fracture (femoral neck fracture or intertrochanteric fracture)	TXA (IV) 1.5 g, pre‐op		Saline, 100 mL (IV), pre‐op	Blood loss Transfusions^a^ LOS Mortality VTE (DVT/PE)
ACTRN 12617000391370 (ongoing study) Australia N = 250 Expected start: 27 March 2017 Expected end: completed	18+ years Intra‐capsular neck of femur fractures undergoing hemiarthroplasty or total hip arthroplasty (within 48 h)	TXA (IV) (15 mg/kg), 3 doses, at induction, and post‐op 8 h and 16 h		Not reported	Transfusion (7days) DVT (7 days)
**Subgroup: hip fixation**					
Haghighi 2017 Single‐centre Iran N = 38	20‐50 years (ASA grade I‐II) Femoral fracture with intramedullary nailing	TXA, IV, 15 mg/kg, pre‐op Mean age: 65 years 14 M, 4 F		Placebo, IV, 15 mg/kg, pre‐op Mean age: 66 years 17 M, 3 F	Transfusions
Lei 2017 Single‐centre China N = 77	Intertrochanteric fracture	TXA, IV, 1 g /200 mL, pre‐op Mean age: 78 years 32 F, 5 M		Placebo (saline), IV, 200 mL, pre‐op Mean age: 79 years 33 F, 7 M	Mortality Transfusions MI CVA/stroke DVT PE
Luo 2019 Multi‐centre China N = 90	60+ years Intertrochanteric fracture treated with PFNA, or closed fracture with low‐energy damage	TXA, IV, 15 mg/kg (body weight), pre‐op, and 3 h later, repeated dose Mean age: 75 years 23 M, 21 F		Placebo (saline), IV, 100 mL, pre‐op Mean age: 76 years 20 M, 26 F	Transfusions CVA/stroke DVT
Ma 2021 Single‐centre China N = 125	65+ years First fresh unilateral femoral intertrochanteric fracture (within 6 h)	IV TXA: 1 g (200 mL) post‐admission (pre‐op) Mean age: 78 years 42 F, 21 M		IV saline (200 mL) post‐admission (pre‐op) Mean age: 79 years 40 F, 22 M	CVA/stroke DVT PE Serious drug reaction
Zhang 2020a Single‐centre China N = 122	18+ years Hip fracture surgery for isolated intertrochanteric fracture treated with PFNA	TXA, IV, 1 g in 100 mL, 10 min pre‐incision (intra‐op) and post‐op Mean age: 79 years 28 M, 33 F		Placebo (saline), IV, 100 mL, 10 min pre‐incision (intra‐op) and post‐op Mean age: 76 years 34 M, 27 F	Mortality Transfusions MI CVA/stroke DVT PE
ACTRN 12620001059954 (ongoing study) Pakistan N = 184 Expected start: 2 Jan 2021 Expected end: not reported	18+ years Trochanteric fracture types AO 31‐A1, A2	1 g TXA (IV), intra‐op		Saline, intra‐op	Transfusions DVT PE Mortality infection
NCT03182751 (ongoing study) USA N = 156 Expected start: 2 April 2018 Expected end: 1 Dec 2022	18+ years AO/OTA fracture classification 31A, surgically treated with sliding hip screw or cephalomedullary nail (short or long)	TXA (IV) 1 g pre‐op, over 8 h		Placebo	Transfusions Blood loss VTE Complication MI CVA Mortality
**Subgroup: mixed**					
Parish 2021 Single‐centre Iran N = 60	18+ years T Type, transverse and associated acetabular fracture (femoral fracture surgery with concher insertion)	TXA IV, 10 mg/kg 15 min before infusion, then infusion at 1 mg/kg/h until end of surgery (intra‐op) Mean age: 44 years 8 F, 22 M		NS (10 mg/kg) 15 min before infusion (intra‐op) Mean age: 47 years 7 F, 23 M	Transfusions DVT PE Serious drug reaction
Sadeghi 2007 Single‐centre Iran N = 67	People with hip fractures with extracapsular fractures treated by plating and nailing, and intracapsular fractures, treated by hemiarthroplasty	TXA, IV, 15 mg/kg, pre‐op (at anaesthesia) Mean age: 52 years 17 M, 15 F		Placebo (saline), IV, 15 mg/kg, pre‐op (at anaesthesia) Mean age: 44 years 24 M, 11 F	Mortality Transfusions DVT PE
ChiCTR 2000032758 (ongoing study) 4‐arm, N = 120 (30 per group) China Expected start: 31 July 2020 Expected end: 31 May 2021	18+ years Unilateral hip fractures (femoral neck fracture, intertrochanteric and subtrochanteric fractures)	15 mg/kg TXA (IV) at 3 different times: Pre‐op Pre‐op + 3 h post‐op Pre‐op + 3 h post‐op + 6 h post‐op		Saline, pre‐op, 3 h post‐op, 6 h post‐op	Blood loss Transfusions VTE
NCT02972294 (HiFIT) (ongoing study) France N = 780 (4‐arm) Expected start: 31 March 2017 Expected end: Oct 2021	18+ years Osteoporotic fractures of the upper end of the femur requiring surgical repair	TXA (IV) + iron (IV) TXA (IV) + iron placebo (IV)		Placebo (saline) (IV) Placebo TXA + iron (IV)	Transfusions Blood loss LOS QOL Mortality Complications
NCT03063892 (ongoing study) USA N = 200 Expected start: 30 Aug 2017 Expected end: 1 Sept 2023	60+ years Hip fracture requiring surgical intervention	TXA (IV) 15 mg/kg, pre‐op, intra‐op, post‐op		Saline (IV), slow over 8 h pre‐op, and intra‐op, post‐op	Transfusions Blood loss
NCT03211286 (ongoing study) Spain N = 129 Expected start: 30 Jan 2018 Expected end: 8 March 2022	60+ years Hip fracture, any surgical procedure	TXA (IV), intra‐op (surgical incision)		Saline (IV)	Transfusions Blood loss Infections Thrombotic events Mortality
NCT03923959 (ongoing study) USA N = 400 Expected start: 1 June 2019 Expected end: 1 Jan 2023	65+ years Hip fracture (femoral neck, intertrochanteric, and subtrochanteric) requiring hemiarthroplasty, total hip replacement, sliding plate and screw fixation, or intramedullary fixation	TXA (IV), 1 g, pre‐op (prior to incision)		Saline (IV), 100 mL, pre‐op	Transfusions Complications Readmission Mortality
ChiCTR 1800018334 (ongoing study) China N = 80 Expected start: 1 Oct 2018 Expected end: 1 July 2019	18‐80 years Acetabular or pelvic fractures	TXA (IV), 10 mg/kg: 3 doses; before incision, 3 h later (intra‐op), 1 g 24 h post‐op		Saline (IV)	Blood loss Transfusion Thrombotic events Wound complications LOS Mortality Readmission
CTRI/2019/09/021302 (ongoing study) India N = 80 Expected start: 1 Oct 2019 Expected end: 12 Nov 2019 Duration: 1 year, 6 months, 15 days	18‐64 years Open reduction surgeries from orthopedic ward	TXA (IV) 10 mg/kg TXA in 100 mL saline, 20 min before incision		Saline, 100 mL	Blood loss (2 days) Transfusions (2 days)
**Subgroup: other **					
Kashefi 2012 Iran N = 80	18‐64 years femoral trunk/shaft surgery	TXA, IV, 15 mg/kg (5 mL), pre‐op Mean age: 43 years 31 M, 9 F		Placebo (saline), IV, 5 mL of liquid (15 mg/kg), pre‐op Mean age: 40 years 33M, 7F	No usable data (translation unclear for group allocation and baseline data)
Monsef Kasmaei 2019 Single‐centre Iran N = 106	18‐60 years Pelvic trauma (within 3 h)	TXA, IV, 1 g, loading dose time point not reported, repeated dose time point not reported Age: not reported 36 M, 17 F		Placebo, IV, 0.9%, time point not reported Age: not reported 29 M, 24 F	No relevant outcomes
TCTR 2021 0311001 Thailand N = 30 Expected start: 23 June 2021 Expected end: 23 Aug 2023	15+ years Non‐union midshaft humerus, undergoing open reduction and plating	TXA (IV), 750 mg, 15 min pre‐op		Placebo, 15 min pre‐op	Blood loss Transfusions
ChiCTR‐ICC‐15006070 (ongoing study) China N = 70 Expected start: 1 Apr 2015 Expected end: 31 Mar 2017	18‐70 years Pelvic trauma	TXA (IV), pre‐op, 10 mg/kg		Saline (IV), pre‐op	Blood loss Transfusion
EUCTR 2018‐000528‐32 (ongoing study) Spain N = 276 Expected start: not reported Expected end: not reported Duration: 1 year, 6 months	64+ years Femur fracture that needs surgical treatment	TXA (IV)		Saline (IV)	Transfusion (30 days) Blood loss
IRCT 2017 1030037093N18 (ongoing study) Iran N = 60 Expected start: 2 Oct 2019 Expected end: 30 Jan 2020	16‐65 years Femoral fixation surgeries	TXA (IV), 15 mg/kg, pre‐op, 30 min before surgery		Saline (IV), 200 mL, pre‐op	Blood loss Transfusions
NCT02428868 (ongoing study) Tunisia N = 150 3‐arms Expected start: April 2015 Expected end: April 2016	60+ years Hip fracture surgery (within 72 h of trauma) Anaemia	TXA (IV) TXA (IV) + iron (IV) TXA: 1 g in 20 mL saline, over 30 min, 5 min before incision, and 1 g 3 h later Iron: 2 x10 mL of 100 mg iron, with TXA (repeat on days 2 and 3)		Placebo (IV), 20 mL saline, over 30 min, 5 min before incision and 3 h later	Transfusion (5 days) Blood loss (5 days) Thromboembolic events (60 days) Infection (60 days) LOS (10 days) Mortality (5, 30, 60 days)
**TXA (topical)** versus **placebo**					
**Subgroup: hip arthroplasty**					
NCT02664909** 2021** Single‐centre USA N = 36	55+ years Hip hemiarthroplasty surgery for a displaced femoral neck fracture	1 g TXA (topical) into surgical wound, at wound closure (intra‐op) Mean age: 83 years 14 F, 3 M	50 mL saline (topical) into surgical wound, at wound closure (intra‐op) Mean age: 83 years 17 F, 2 M		Mortality Transfusions MI DVT
**Subgroup: mixed**					
Costain 2021 Single‐centre Canada N = 65	18+ years Hip fracture: intracapsular, intratrochanteric or subtrochanteric	3 g TXA, topical, intra‐op Mean age: 80 years 20 F, 11 M	50 mL saline; topical, intra‐op Mean age: 79 years 25 F, 9 M		Transfusions CVA/stroke DVT
NCT01727843** 2018** Single‐centre Canada N = 15 (terminated prematurely)	65+ years Hip fracture	3 g TXA, topical, end of surgery (intra‐op) Age: not reported Gender: not reported	3 g saline; topical, end of surgery (intra‐op) Age: not reported Gender: not reported		No data available (terminated prematurely)
ChiCTR 1900021948 (ongoing study) China 4‐arm trial N = 200 (50 per group) Expected start: 1 Apr 2019 Expected end: 31 Mar 2021	18+ years Hip fracture treated with any surgical procedure	TXA (route unclear) + IV iron TXA (route unclear) + IV placebo N = 100	Placebo (route unclear) + IV iron Placebo (route unclear) + IV placebo N = 100		Transfusion Blood loss LOS Wound complication Transfusion‐related events Readmission Mortality
**Subgroup: other**					
ChiCTR 1800014309 (ongoing study) China N = 100 Expected start: 10 Jan 2018 Expected end: 1 Jan 2020	60+ years Intertrochanteric fracture treated with PFNA	TXA 1 g, into proximal medullary cavity	Saline, 20 mL, into proximal medullary cavity		Blood loss Transfusion Thrombotic events (6 weeks) Mortality (6 weeks)
**New comparison: TXA (tablet + injection)** versus **placebo (tablet + injection)**					
**Subgroup: hip fixation**					
CTRI/2021/09/036855 (ongoing study) India N = 100 Expected start: 30 Sept 2021 Expected end: not reported (1 year, 8 months, 10 days later)	18‐75 years major periarticular hip surgeries	TXA (tablets), 1950 mg + 2 g TXA (injection) in post‐op drain	Saline, (tablets) pre‐op and saline (injection) in post‐op drain		Blood loss Transfusions Thromboembolic events
**AO/OTA:** fracture classification**; ASA:** American Society of Anesthesiologists; **CVA:** cerebrovascular accident; **DVT:** deep vein thrombosis; **F:** female; **IV:** intravenous; **LOS:** length of stay; **M:** male; **MI:** myocardial infarction; **NS:** normal saline; **PE:** pulmonary embolism; **PFNA:** proximal femoral nail anti‐rotation; **QOL:** quality of life; **TXA:** tranexamic acid; **VTE:** venous thromboembolism					

^a^'Transfusions' relates to the reporting of the proportion of participants who required allogeneic blood transfusion.

**7:** All studies (included and ongoing): tranexamic acid versus other tranexamic acid

**Study**	**Participants (inclusion criteria)**	**Intervention**	**Comparator **	**Outcomes**
**TXA (local)** versus **TXA (IV)**				
**Subgroup: hip arthroplasty**				
TCTR 2021 0316006 (ongoing study) Thailand N = 130 Expected start: 1 June 2021 Expected end: 31 Aug 2023	60+ years Displaced femoral neck fracture treated with bipolar hemiarthroplasty	TXA (topical), 3 g, femoral canal and under fascia, intra‐op (after closed wound)	TXA (IV), 20 mg/kg, pre‐op	Blood loss Transfusions^a^ Complications
ChiCTR 1800015809 (ongoing study) China N = 360 (60 per group) 4‐arm trial Expected start: 1 May 2018 Expected end: 31 Aug 2018	18‐85 years Femoral neck fracture and total hip arthroplasty	TXA (topical) N = 60	TXA (IV) N = 60	Blood measurement Inflammation Function
NCT02938962 (ongoing study) Canada N = 160 Expected start: Oct 2016 Expected end: Nov 2018	18+ years Revision hip arthroplasty	TXA (topical), 100 mL solution (3 g TXA in 100 mL of NS) instilled into the surgical field throughout the operative procedure	TXA (IV), single 20 mg/kg dose of TXA prior to the skin incision	Transfusions (4 days) LOS Blood loss (intra‐op) Complications (3 months)
**Subgroup: hip fixation**				
CTRI/2019/10/021667 (ongoing study) India N = 120 (3‐arms: third arm is standard care, not relevant) Expected start: 1 Nov 2019 Expected end: not reported	18‐80 years 1. Evans types 1 and 2 2. Internal fixation by dynamic hip screw	1 g TXA (local), intramuscular, intra‐op	10 mg/kg TXA (IV), pre‐op and 2 h later	Transfusions Complications Blood loss
**New comparison: TXA (IV)** versus **TXA (oral)**				
**Subgroup: hip arthroplasty**				
ChiCTR 1800015809 (ongoing study) China N = 360 (60 per group) 4‐arm trial Expected start: 1 May 2018 Expected end: 31 Aug 2018	18‐85 years Femoral neck fracture and total hip arthroplasty	TXA (IV) N = 60	TXA (oral) – 2 groups: 2 g (n = 60) and various doses (n = 180)	Blood measurement Inflammation Function
**New comparison: TXA (topical) versus TXA (oral)**				
**Subgroup: hip arthroplasty**				
ChiCTR 1800015809 (ongoing study) China N = 360 (60 per group) 4‐arm trial Expected start: 1 May 2018 Expected end: 31 Aug 2018	18‐85 years Femoral neck fracture and total hip arthroplasty	TXA (topical) N = 60	TXA (oral) – 2 groups: 2 g (n = 60) and various doses (n = 180)	Blood measurement Inflammation Function
**New comparison: TXA (different administration)**				
**Subgroup: hip fixation**				
CTRI/2019/04/018735 (ongoing study) India N = 30 Expected start: 1 May 2019 Expected end: 21 February 2020	18‐65 years surgery for pelviacetabular fracture under regional anaesthesia.	TXA (IV) bolus (1 g over 10 min) + TXA (continuous infusion 1 mg/kg/h for 4 h)	TXA (IV) bolus (1 g over 10 min)	Blood loss (24 h) DVT (24 h)
**Subgroup: mixed**				
ChiCTR‐IPR‐17013477 (ongoing study) China 3‐arm trial (100 per arm; only 2 arms relevant) N = 200 Expected start: 1 Mar 2018 Expected end: 31 Dec 2019	18+ years Spinal internal fixation, internal fixation of acetabular fractures, internal fixation of femoral shaft fractures, internal fixation of pelvic fractures, internal fixation of humeral shaft fractures, internal fixation of proximal people with humerus fractures undergoing total hip arthroplasty	TXA (intermittent)	TXA (continuous)	Blood loss Transfusion DVT
**DVT:** deep vein thrombosis; **IV:** intravenous; **LOS:** length of stay; **NS:** normal saline; **TXA:** tranexamic acid				

^a^'Transfusions' relates to the reporting of the proportion of participants requiring allogeneic blood transfusion.

**8:** All studies (included and ongoing): tranexamic acid versus non‐tranexamic acid

**Study**	**Participants (inclusion criteria)**	**Intervention**	**Comparator **	**Outcomes**
**New comparison: TXA** versus **fibrin glue**				
**Subgroup: hip arthroplasty**				
EUCTR 2011‐006278‐15 (ongoing study) Spain N = 220 Expected start: not reported Expected end: not reported	18+ years Unilateral subcapital femoral fracture, requiring hip replacement	TXA (topical)	Fibrin glue (topical)	Blood loss (24 h) Transfusion Wound infection LOS Side effects Mortality
**LOS:** length of stay; **TXA:** tranexamic acid				

^a^'Transfusions' relates to the reporting of the proportion of participants requiring allogeneic blood transfusion.

**9:** All studies (included and ongoing): non‐tranexamic acid versus placebo

**Study**	**Participants (inclusion criteria)**	**Intervention**	**Comparator **	**Outcomes**
**rFVIIa** versus **placebo**				
**Subgroup: other **				
**Raobaikady 2005** Single‐centre UK N = 48	18‐60 years Major pelvic–acetabular fracture caused by trauma, requiring “large” reconstruction	rfVIIa, IV, 90 µg/kg, intra‐op Age: median 44 years 16 M, 8 F	Placebo, IV, 90 µg/kg, intra‐op Age: median 38 years 18 M, 6 F	Transfusions^a^ Reoperation
**New comparison:** f**ibrinogen (injection)** versus **placebo (injection)**				
**Subgroup: other**				
IRCT 2020 0109046064N1 (ongoing study) Iran N = 42 Expected start: 20 February 2020 Expected end: 20 April 2020	18‐60 years Non‐emergency pelvic surgery	Fibrinogen, 1 g injected, intra‐op	Placebo, saline, intra‐op	Plasma fibrinogen
**F:** female; **M:** male; **rFVIIa:** recombinant factor VIIa				

^a^'Transfusions' relates to the reporting of the proportion of participants requiring allogeneic blood transfusion.

### Summary of main results

We grouped the data into three comparisons of interest.

#### Comparison 1: intravenous tranexamic acid versus placebo

We found the most data for this comparison. See Table 1

Intravenous tranexamic acid may reduce the risk of requiring allogeneic blood transfusion, based on evidence from six trials: four trials in people undergoing hip fixation (Haghighi 2017; Lei 2017; Luo 2019; Zhang 2020a), and two trials in a mixed population (Parish 2021; Sadeghi 2007).

Intravenous tranexamic acid may result in little to no difference in all‐cause mortality (2 RCTs; hip fixation: Lei 2017; mixed: Sadeghi 2007), risk of myocardial infarction (2 RCTs; hip fixation: Lei 2017; Zhang 2020a), and cerebrovascular accident/stroke (3 RCTs; hip fixation: Lei 2017; Ma 2021; Zhang 2020a).

We are uncertain if intravenous tranexamic acid has any impact on risk of deep vein thrombosis (4 RCTs; hip fixation: Lei 2017; Ma 2021; mixed: Parish 2021; Sadeghi 2007), pulmonary embolism (4 RCTs; hip fixation: Lei 2017; Ma 2021; mixed: Parish 2021; Sadeghi 2007), and suspected serious drug reaction (2 RCTs; hip fixation: Ma 2021; mixed: Parish 2021). 

No other outcomes of interest were reported.

#### Comparison 2: topical tranexamic acid versus placebo

See Table 2. We are uncertain if topical tranexamic acid has any impact on the risk of requiring allogeneic blood transfusion, mortality, or adverse events (myocardial infarction, cerebrovascular accident/stroke, deep vein thrombosis), based on the evidence from two trials: in people undergoing hip arthroplasty (NCT02664909), and in a mixed population (Costain 2021). No other outcomes of interest were reported.

#### Comparison 3: recombinant factor VIIa versus placebo

Based on the evidence from one trial in people undergoing pelvic surgery (Raobaikady 2005), we are uncertain whether recombinant factor V11a has any impact on the risk of requiring allogeneic blood transfusion, reoperation due to bleeding, risk of deep vein thrombosis, pulmonary embolism, or suspected serious drug reaction. No other outcomes of interest were reported.

### Overall completeness and applicability of evidence

We excluded all studies published after 2010 that were unregistered, or retrospectively registered, as per our protocol (Gibbs 2019a), and in line with Cochrane Injuries Editorial Policy (Broughton 2021; Cochrane policy; Roberts 2015). This may have excluded some relevant and useful studies from the review (Excluded studies). As a result, our review included comparatively few trials exploring pharmacological interventions to prevent bleeding in hip, pelvic and long bone fractures.

We included one study related to femoral shaft fixation and three relating to pelvic and acetabular fracture studies. The remaining 10 studies assessed bleeding in people with hip fractures. With this spread it is very difficult to generalise the findings to other long bone fractures. Hip fractures were by far the most studied population and had the highest number of prospectively registered RCTs (Table 4; Table 5; Table 6). Tranexamic acid was the most common intervention studied and the routes used were intravenous and topical (Table 4; Table 5). The demographic of the participants within the trials differed between hip fracture trials and pelvic/acetabular and femoral shaft fractures, as we would expect. This is likely related to the injury sustained: hip fractures are typically sustained in an older population due to a reduction in bone quality and associated co‐morbidities, whereas pelvic/acetabular and femoral shaft fractures are more likely to be sustained with a higher energy injury, and are often associated with polytrauma injuries. Polytrauma injuries is the subject of a different Cochrane Review that is currently underway (Erasu 2022).

Trials were conducted in a variety of countries., Only one included study assessed a non‐tranexamic acid intervention (Raobaikady 2005 used recombinant coagulation factor VII). Only a few ongoing trials are investigating non‐tranexamic acid interventions, as described in Table 10 and Table 11. 

We were unable to perform any meaningful subgroup analysis with the available data. Furthermore, we were unable to perform an NMA due to inadequate data and therefore have reported pairwise analyses only. We were not able to explore the optimal route, dose or timing of tranexamic acid as we had hoped in our protocol, as all doses were similar (approximately 15 mg/kg), and more research is required to delineate the optimal dose and route of tranexamic acid administration. We hope to perform an update of this review when more data become available from trials currently underway (Characteristics of ongoing studies: Table 8; Table 9; Table 10; Table 11).

All included studies were small, and at moderate to high risk of bias. Of our primary outcomes, four studies did not report the requirement for allogeneic blood transfusion, and only three studies reported all‐cause mortality within 30 days.  

Our evidence is also limited by the lack of analysable data regarding volume of blood (mean red blood cell units) transfused due to the reporting, interpretation, and analysis of skewed data (presented as median and range or IQR): some studies reported the total number of red blood cell units transfused, to the whole group, or the number of participants who required more than a specific number of red blood cell units (e.g. the number pf people requiring more than one, two, three, or four units of blood), though this was reported inconsistently across trials. Unfortunately, we were unable to convert these data for this review, as we had specified a continuous outcome using the mean and SD. We also encountered issues in interpreting the mean and standard deviation (SD) reported, as it could not be confirmed whether these data were for all participants randomised, or for only those who had been transfused. Where we could ascertain this information, often we could not analyse the data, as one arm had zero transfusions (mean 0, SD 0, N = 0). Due to the variability in the need for red blood cell units ‐ as the expectation is that most people require very few units and one or two people may require upwards of 20 units in cases of extreme blood loss ‐ a significant portion of the data are skewed, and so are presented as median and IQR, or median and range.

Consequently, in future updates of this review, we will consider introducing an additional dichotomous variable to assess the number of participants who required more than a set number of units to be transfused, to highlight where there is greater need for further intervention. 

More robust trials are required to draw any firm conclusions for pharmacological interventions for the prevention of bleeding in hip, pelvic, and long bone fractures. There may be some benefit to using tranexamic acid intravenously for the prevention of bleeding in people with hip fractures, however this is based on very low‐certainty evidence, and further evidence from high‐quality trials is required. 

### Quality of the evidence

Overall, we rated the certainty of the evidence according to GRADE methodology across all comparisons for the outcomes of risk of requiring allogeneic transfusion, all‐cause mortality, reoperation due to bleeding, and adverse events as very low to low (Table 1; Table 2).

We downgraded certainty of the evidence for imprecision (wide confidence intervals around the estimate and small sample size, particularly for rare events), and risk of bias (unclear or high‐risk methods of blinding and allocation concealment in the assessment of subjective measures, and high risk of attrition and reporting biases).

The studies were very small, far below the optimal information size for rare events associated with long bone trauma (specifically mortality, stroke, deep vein thrombosis, pulmonary embolism, and myocardial infarction). Power or sample size calculations were only reported by nine of the 13 included studies, of which only four achieved their required sample size, significantly weakening the results. The trial authors did not base the power calculation on these rare events (mortality, stroke, deep vein thrombosis, pulmonary embolism, myocardial infarction), largely using blood loss or change in haemoglobin or haematocrit, which we did not assess.

We were unable to assess publication bias using a funnel plot, as there were not enough studies per comparison and outcome (fewer than 10 studies).

### Potential biases in the review process

We have attempted to minimise bias in the review process. We conducted a comprehensive search: we searched multiple data sources (including multiple databases, and clinical trials registries) to ensure that all relevant studies would be captured. There were no restrictions for the language in which reports were originally published. We assessed the relevance of each publication carefully and performed all screening and data extractions in duplicate. We prespecified all outcomes and subgroups prior to analysis. We were unable to assess publication bias using funnel plots as no individual outcome in a single comparison included enough studies (fewer than 10 studies).

We excluded trials that did not prospectively register their protocol (for publications since 2010) to minimise potential for bias from the included data, though we accept this may have excluded some relevant and useful studies. However, the decision to exclude unregistered (or retrospectively registered) was taken due to the evidence highlighting issues surrounding false data, including the possibility of 'zombie' trials, where a trial did not even take place (Carlisle 2021; Roberts 2015). Prospective registration reduces the chance of publication bias, and has been compulsory for RCTs since 2005, thus suggesting that those that have not been registered (or registered retrospectively) are less likely to be of low risk of bias (Roberts 2015).

### Agreements and disagreements with other studies or reviews

Two recent systematic reviews have explored the effectiveness of tranexamic acid in reducing blood loss (Haj‐Younes 2020; Masouros 2021). These studies concluded that tranexamic acid reduced blood loss and the need for transfusion in people with hip fractures undergoing surgery. Masouros 2021 suggested that the optimal dose of tranexamic acid for prevention of bleeding was 15 mg/kg. Furthermore, this review reported that the overall reduction in total blood loss following use of tranexamic acid was 240 mL, though the authors acknowledged that the quality of the evidence may be limited by the small number of studies included (10 studies). Masouros 2021 included seven trials (834 participants) that we excluded from this review because they were retrospectively registered (Nikolaou 2021; Tengberg 2016; Watts 2017; Zufferey 2010), we were unable to confirm trial registration from the author (Chen 2019; Tian 2018), or the trial author confirmed that the trial had not been registered (Zhou 2019).

Haj‐Younes 2020 reported that tranexamic acid reduced the need for blood transfusion in people with hip fractures by 25%, with no significant increase in mortality, thromboembolic events or wound complications. Both reviews found that a dose of between 10 mg/kg to 15 mg/kg of tranexamic acid reduced the need for allogeneic blood transfusion. We were unable to draw such conclusions for people sustaining a hip fracture due to the lack of high‐quality evidence available. Haj‐Younes 2020 included data from six trials (570 participants) that we excluded from this review because they were retrospectively registered (Tengberg 2016; Watts 2017; Zufferey 2010), we were unable to confirm trial registration from the author (Tian 2018; Vijay 2013), or the author confirmed that the trial had not been registered (Baruah 2016).

A recent systematic review investigated tranexamic acid use in people undergoing pelvic/acetabular fracture surgery (Shu 2021). They included four studies in their review: two were retrospective cohort studies and two were RCTs; one that we found to be retrospectively registered (Lack 2017), and another that used usual care as the comparator (Spitler 2019). Of the three studies that were combined in the meta‐analysis (308 participants) the authors found that tranexamic acid reduced the need for blood transfusion, however, they acknowledged that very few trials contributed to this finding. We identified one ongoing study assessing the use of tranexamic acid in pelvic/acetabular fractures (ChiCTR‐ICC‐15006070), which may provide more information in the future. 

We were able to identify only two ongoing studies assessing tranexamic acid use in femoral shaft fractures (IRCT 2017 1030037093N18, EUCTR 2018‐000528‐32). We were not able to find any other systematic reviews looking at people requiring definitive fixation for long bone fractures.

In this review, we have focused exclusively on people undergoing trauma (non‐elective) surgeries, excluding those studies that had a mixed population where we could not separate the relevant data. Our sister review focused on elective surgery only (Gibbs 2019b), and identified sufficient data to undertake some of the network analyses described in both reviews. The certainty of the evidence in that review varied from low to high across the networks and pairwise analyses for elective (planned) surgery, with similar reasons for downgrading the evidence as in this review: unclear or lack of true randomisation processes (baseline imbalances), and imprecision (wide confidence intervals and small sample sizes, especially for rarer outcomes such as mortality). The elective and trauma reviews found similar gaps in the literature surrounding this topic, including poor study design (within‐study heterogeneity from mixed populations with no subgrouping, per‐protocol analysis instead of intention‐to‐treat), few interventions of interest, unregistered (or retrospectively registered) trials, or discrepancies between the published protocol or trial registration and the published data, and limited reporting of important outcomes (e.g. number of red blood cell units transfused, and adverse events: transfusion reactions, suspected drug reactions, need for reoperation).

## Authors' conclusions

**Implications for practice**

We are unable to draw any strong conclusions about the use of interventions to reduce blood loss in people undergoing definitive fixation of hip, pelvic, and long bone fractures due to the lack of data. The included studies predominately concern the use of tranexamic acid, and most were performed in people with hip fractures. Our review suggests that tranexamic acid may be effective at reducing the need for transfusion in people requiring hip fracture surgery, thereby suggesting a reduction in blood loss, but more evidence is required to state this with certainty. 

Several ongoing studies are due to be completed by the end of 2023, so an update of this review from 2025 onwards may enable us to re‐assess the effectiveness of tranexamic acid to reduce blood loss and the need for transfusion during definitive fixation of hip, pelvic, and long bone fractures (Table 8; Table 9; Table 10) alongside other interventions being trialled. If all ongoing studies complete and publish, this would enable us to add assessment of 27 new trials with a total of 4177 participants, in addition to the 13 already included in our analyses.  

**Implications for research**

We have identified a number of areas where the quality and quantity of relevant data available for this review could be improved, which are presented below.

**Trial registration**

By far the most common preventable reason for exclusion of trials from this review was the lack of prospective trial registration, whether the trial remained unregistered, or was registered retrospectively (after recruitment or randomisation, or both, had already started). Prospective trial registration for drug interventions became compulsory in 2005, and we did not expect to identify such a high number of trials (63) that did not fulfil this requirement. We encourage future researchers to actively pursue prospective registration on national and international databases, in order to allow complete transparency in the design of the trial, and an audit trail for any changes that may have been made (with rationale for those changes) during the various study phases (active recruitment, through to data analysis and publication or dissemination, or both).

**Participants (potential risk modifiers)**

We found very few research studies exploring pharmacological interventions to prevent bleeding in the definitive fixation of hip, pelvic, and long bone fractures. Tranexamic acid has been studied, but really only in the context of hip fractures, and clear evidence for its benefits in pelvic and long bone fractures remains unknown. The predominance of data from hip surgery is in line with the incidence of these types of surgery in the general population each year (Wu 2021). Additionally, it is likely that the research focus has been largely in hip fracture (arthroplasty or fixation) due to the homogeneous population and a standardised surgical procedure, the high prevalence of preoperative anaemia and thus high risk of blood transfusion, and a high rate of post‐operative complications and death in this population, which also contribute to a high economic burden. However, it remains important to expand the evidence base of surgery of the pelvis and long bone as well.

Other potential risk modifiers (or potential subgroups in a pairwise analysis) that we identified a priori, include the incidence of preoperative anaemia, and the use of anticoagulants, or antiplatelets, or both, at the time of injury. These characteristics were largely unreported in the included studies, though their impact on the intervention effectiveness could be important.

**Interventions and comparators**

The most studied intervention included in this review was tranexamic acid, administered intravenously, topically or locally, or as a combination of the two treatment modalities. Exploration of the effectiveness of alternative pharmacological agents to tranexamic acid in hip, pelvic, and long bone fractures remains largely unexplored. While it is likely that tranexamic acid is the most effective intervention, based on evidence for other orthopaedic procedures, there may be some benefit to exploring other pharmacological interventions. 

Several ongoing studies exploring tranexamic acid are yet to be completed and published, and may provide more insight into the most effective route, dose, and timing of administration. 

**Outcome reporting**

In the Results we have described the evidence for 10 outcomes, of which seven are presented in the summary of findings tables, and deemed most important for this review. Of these outcomes, there was little to no information available for the mean number of red blood cell unit transfused (in units of blood or another measure of volume), the need for reoperation due to bleeding, and the incidence of acute transfusion reaction or suspected serious drug reaction (as defined by the International Conference on Good Clinical Practice (ICH GCP 2018), though this was usually reported as 'number of adverse events related to [the drug]'). 

As mentioned in the Discussion (Overall completeness and applicability of evidence), we encountered a number of issues surrounding the reporting, interpretation, and analysis of the average (mean) volume of red blood cell units due to lack of clarity on what was being reported (whether based on the number of people randomised, or the number of people transfused, and issues arising for analysis where no one was transfused in one arm). We therefore encourage researchers to be clear with regard to their analysis (mean and standard deviation, or median interquartile range depending on skewness, of red blood cell units per participant randomised, or per participant transfused), and also present categories of the number of red blood cell units transfused (e.g. number of participants requiring one, two, three, four, or five or more units) to aid future analyses.

Ideally, the current ongoing studies and future trials should report these important outcomes to provide a full picture of any adverse events that may affect the risk profile, and recovery process, of each individual who may experience a transfusion or drug reaction. The need for reoperation may also impact the economic profile of chosen interventions, though we have not focused on cost here.

Additionally, whilst we had planned to perform an overall analysis of thromboembolic events, we have presented the various diagnoses separately (pulmonary embolism, myocardial infarction, cerebrovascular accident/stroke, deep vein thrombosis), as they were not consistently reported: some studies only reported one or other, but did not state they had zero cases of other thromboembolic events, and we could not assume this. Moving forward, we encourage researchers to report any and all thromboembolic events, both individually (as pulmonary embolism, myocardial infarction, stroke, deep vein thrombosis, etc.), and as the number of people experiencing any thromboembolic event (in case some people had multiple events).

**Timing and follow‐up**

Whilst we have defined our follow‐up period as up to and including 30 days for most outcomes, some studies reported longer than this instead (up to 90 days), or 'in‐hospital stay'. Where average length of stay was unreported (for in‐hospital stay), we have assumed this was within the defined 30 days, or have inferred data where zero cases or events were reported. We encourage future studies to report a defined time period, and report at regular intervals within that time period (e.g. up to 14 days, 30 days, 60 days), especially where follow‐up is lengthy (up to three months and more) or in the case of people experiencing trauma, as they are more likely to have a wider range of inpatient care.  

## What's new

**Table:** 

**Date**	**Event**	**Description**
23 June 2023	Amended	Error in order of authors corrected

## History

Protocol first published: Issue 12, 2019 Review first published: Issue 6, 2023

## Appendices

### Appendix 1. Methods specific to network meta‐analyses for future review updates

#### Methods specific to network meta‐analyses (NMAs) for future review updates

Processes of identifying, selecting, and extracting data remain the same for the pairwise and network meta‐analysis (NMA) process. Only sections relating to NMAs that differ from the pairwise method have been described here, and in the full protocol, available from Gibbs 2019a.

##### Assessment of heterogeneity

In future updates, if the extracted data appear to be homogeneous, we will amalgamate the data and undertake an NMA. We will look for clinical and methodological heterogeneity within each comparison by comparing trial and baseline characteristics across the included trials. If we find important clinical or methodological heterogeneity, we may not be able to perform a meta‐analysis. If this is the case, we will provide a descriptive summary instead.

When performing the NMA, we will assume a common estimate for heterogeneity across all our comparisons, and we will estimate a value for the total I² statistic value across the network. We will assess statistical heterogeneity across the whole network based on the magnitude of the heterogeneity variance parameter (Tau²), which we will estimate from the NMA models. We will perform a likelihood ratio test for the null hypothesis of no heterogeneity versus presence of heterogeneity.

##### Data synthesis

In future updates, we will use Stata to undertake a multivariate NMA which will treat each comparison as a different outcome. The analyses will be done using the network package in Stata (Stata 2017). We will provide the estimated treatment effect for each comparison with a 95% confidence interval (CI). 

Where appropriate, we will categorise interventions into clinically meaningful groups during the first stage of data extraction. Each group will act as a single node within the network. We will run sensitivity analyses using different groupings. Each group will contain one type of pharmacological intervention, for example, only tranexamic acid, but may include a narrow dose range, route and timing variables, to have a pharmacologically similar predicted effect.

###### Potential risk modifiers

In order to perform meta‐regression, we will extract data on the following characteristics, which may behave as treatment risk modifiers in a future review update.

- **Type of surgery:** different types of definitive fixation surgery are likely to result in different volumes of blood loss. We expect that the effect of the interventions will be greater in surgery with greater blood loss, therefore, we will examine this through subgroup analysis according to the expected amount of blood loss in the different types of surgery.
  - Group 1: pelvic fixation, revision joint replacement for periprosthetic hip/knee fracture, femoral fixation and neck of femur intramedullary nailing (the highest risk of bleeding)
  - Group 2: hip joint replacement surgery (hip hemiarthroplasty, total hip replacement) knee joint replacement (high risk of bleeding)
  - Group 3: cannulated hip screws, dynamic hip screw, tibial fixation, shoulder replacement surgery, humerus fixation (lower risk of bleeding)
  - Group 4: elbow replacement surgery, clavicle fixation, fibula fixation, radius fixation and ulna fixation (the lowest risk of bleeding)
- **Incidence of preoperative anaemia:** after surgery, people with anaemia are likely to have a higher risk of needing blood transfusion (Sim 2018), an increased length of hospital stay (Abdullah 2017), and an increased risk of complications (Viola 2015). We expect that the effect of the interventions will vary depending on the presence or absence of preoperative anaemia, with the treatment being less effective and resulting in greater reported complication rates in people with preoperative anaemia. We will examine this through subgroup analysis of participants with and without preoperative anaemia.
- **Consumption of anticoagulant or antiplatelet drugs at the time of injury:** people taking these medications are likely to bleed more. A previous review reported that desmopressin, an intervention of interest, may be effective at reducing the need for blood transfusion in people taking antiplatelet drugs (Desborough 2017). We anticipate that the interventions will be more effective in people taking anticoagulants or antiplatelets. We will examine this through subgroup analysis of participants taking these medications and those who were not.

##### Subgroup analysis and investigation of heterogeneity

###### Investigation of heterogeneity

In future updates, for the NMA, we will estimate the heterogeneity variance parameter Tau²and use it to assess statistical heterogeneity within the network. With any NMA, we will also estimate a total I² statistic for the whole network (see Assessment of heterogeneity).

###### Assessment of transitivity

In future updates, where NMA is possible, we will evaluate the assumption of transitivity by comparing the distribution of effect modifiers (listed above) across different comparisons (Chaimani 2022). We will assess incoherence and inconsistency of each network both locally (evaluate regions of the network separately to detect possible 'incoherence spots') and globally (evaluate coherence in the entire network) using the *ifplot* macro available for Stata (Chaimani 2015). We will consider the confidence intervals for incoherence factors, and decide whether they include values that are sufficiently large to suggest clinically important discrepancies between direct and indirect evidence.

If we have any concern that clinical safety and effectiveness are dependent upon effect modifiers, we will continue to do traditional Cochrane pair‐wise comparisons and will not perform a network meta‐analysis on all participant subgroups.

###### Assessment of statistical inconsistency

In future updates, where an NMA is possible, and to gauge any inconsistency within each loop of the network, we will use the 'loop' inconsistency model of Lu and Ades (Lu 2006), using the 'luades' option in Stata (Stata 2017). This will give an assessment of consistency within each loop of the network. If there are no closed loops, we will calculate transitivity to determine the presence of inconsistency. We will assume there is common heterogeneity within each loop. We will present results in a forest plot through the network graphs package in Stata (frequentist analysis approach). If we find evidence of global inconsistency, we will use the node‐splitting method to explore this further (Dias 2010).

##### Summary of findings and assessment of the certainty of evidence

In future updates, where NMA is possible, we will evaluate the confidence of the evidence using the CINeMA framework (Confidence in Network Meta‐Analysis; Salanti 2014). We will use the online CINeMA tool which assesses confidence for each comparison within the network and is based on: within‐study bias, across‐studies bias, indirectness, imprecision, heterogeneity and incoherence (CINeMA 2017).

##### Ranking interventions

We will present effect estimates with 95% credible interval (CrI) for each pair‐wise comparison calculated from direct comparisons and network meta‐analysis. We will present the cumulative probability of treatment ranks (i.e. the probability that the treatment is within the top two, the probability that the treatment is within the top three, etc.) in graphs (surface under the cumulative ranking curve, or SUCRA) (Salanti 2011). We will plot the probability that each treatment is best, second best, third best, etc. for each of the different outcomes (rankograms), which generally are considered more informative (Chaimani 2022; Salanti 2011).

### Appendix 2. Search strategies

#### CENTRAL (The Cochrane Library)

#1 MeSH descriptor: [Femoral Fractures] explode all trees

#2 MeSH descriptor: [Ankle Fractures] this term only

#3 MeSH descriptor: [Humeral Fractures] this term only

#4 MeSH descriptor: [Osteoporotic Fractures] this term only

#5 MeSH descriptor: [Periprosthetic Fractures] this term only

#6 MeSH descriptor: [Shoulder Fractures] explode all trees

#7 MeSH descriptor: [Tibial Fractures] this term only

#8 MeSH descriptor: [Ulna Fractures] this term only

#9 MeSH descriptor: [Radius Fractures] explode all trees

#10 MeSH descriptor: [Fractures, Bone] this term only

#11 ((pelvi* or sacrum or coccyx or ischium or pubis or pubic or ilium or tailbone or diaphys* or epiphys* or metaphys* or acetabulum or acetabular or femor* or femur* or hip* or thigh* or tibia* or fibula* or intertrochanteric or subtrochanteric or petrochanteric or intracapsular or subcapsular or subcapital or osteoporo* or osteoarthritis or orthop?edic) near/6 (fracture* or break* or broke* or trauma* or injur* or surg* or operat* or repair* or reconstruct* or fixation* or implant* or prosthes* or "plate and screw" or "plate and screws" or "intramedullary nail" or "intramedullary nails")):ti,ab

#12 (("long bone" or "long bones" or long‐bone* or humerus or humeral or "upper arm" or "upper arms" or shoulder* or clavicle* or clavicula* or "collar bone" or "collar bones" or ankle* or pilon or "lower leg" or "lower legs" or calf* or knee* or tibiofibular or menisci or meniscus or femoropatellar or patellofemoral or radial or radius or ulna or forearm* or elbow*) near/6 (fracture* or break* or broke* or trauma* or injur* or surg* or operat* or repair* or reconstruct* or fixation* or implant* or prosthes* or "plate and screw" or "plate and screws" or "intramedullary nail" or "intramedullary nails")):ti,ab

#13 ((malleol* or talus or trochanteric or crural or crus or olecranon or antebrachial or monteggi* or bankart) near/6 (fracture* or break* or broke* or trauma* or injur* or surg* or operat* or repair* or reconstruct* or fixation* or implant* or prosthes* or "plate and screw" or "plate and screws" or "intramedullary nail" or "intramedullary nails")):ti,ab

#14 ((wrist* or capitate or hamtae or lunate or carpal or metacarpal or pisiform or scaphoid or trapezium or triquetral) near/6 (fracture* or break* or broke* or trauma* or injur* or surg* or operat* or repair* or reconstruct* or fixation* or implant* or prosthes* or "plate and screw" or "plate and screws" or "intramedullary nail" or "intramedullary nails")):ti,ab

#15 ((peri‐implant or periprosthetic) near/1 fracture*)

#16 MeSH descriptor: [Pelvic Bones] explode all trees and with qualifier(s): [injuries ‐ IN, surgery ‐ SU]

#17 MeSH descriptor: [Leg Bones] explode all trees and with qualifier(s): [injuries ‐ IN, surgery ‐ SU]

#18 MeSH descriptor: [Arm Bones] explode all trees and with qualifier(s): [injuries ‐ IN, surgery ‐ SU]

#19 MeSH descriptor: [Clavicle] explode all trees and with qualifier(s): [injuries ‐ IN, surgery ‐ SU]

#20 MeSH descriptor: [Bones of Upper Extremity] this term only and with qualifier(s): [injuries ‐ IN, surgery ‐ SU]

#21 MeSH descriptor: [Bones of Lower Extremity] this term only and with qualifier(s): [injuries ‐ IN, surgery ‐ SU]

#22 MeSH descriptor: [Hip Joint] explode all trees and with qualifier(s): [surgery ‐ SU]

#23 MeSH descriptor: [Shoulder Joint] this term only and with qualifier(s): [surgery ‐ SU]

#24 MeSH descriptor: [Knee Joint] explode all trees and with qualifier(s): [surgery ‐ SU]

#25 MeSH descriptor: [Ankle Joint] this term only and with qualifier(s): [surgery ‐ SU]

#26 MeSH descriptor: [Elbow Joint] this term only and with qualifier(s): [injuries ‐ IN, surgery ‐ SU]

#27 MeSH descriptor: [Hip Injuries] explode all trees and with qualifier(s): [surgery ‐ SU]

#28 MeSH descriptor: [Knee Injuries] explode all trees and with qualifier(s): [surgery ‐ SU]

#29 MeSH descriptor: [Lower Extremity] this term only and with qualifier(s): [surgery ‐ SU, injuries ‐ IN]

#30 MeSH descriptor: [Upper Extremity] this term only and with qualifier(s): [surgery ‐ SU, injuries ‐ IN]

#31 ((hip* or shoulder* or knee*) near/5 (replac* or arthroplast* or hemiarthroplast* or hemi‐arthroplast*)):ti,ab

#32 MeSH descriptor: [Bones of Lower Extremity] explode all trees

#33 MeSH descriptor: [Bones of Upper Extremity] explode all trees

#34 #32 or #33

#35 MeSH descriptor: [Fracture Fixation] explode all trees

#36 (trauma* or fracture* or injur* or surg* or operat* or repair* or fixation):ti

#37 #35 or #36

#38 #34 and #37

#39 #1 or #2 or #3 or #4 or #5 or #6 or #7 or #8 or #9 or #10 or #11 or #12 or #13 or #14 or #15 or #16 or #17 or #18 or #19 or #21 or #22 or #23 or #24 or #25 or #26 or #27 or #28 or #29 or #30 or #31 or #38

#40 MeSH descriptor: [Antifibrinolytic Agents] this term only

#41 MeSH descriptor: [Tranexamic Acid] this term only

#42 MeSH descriptor: [Aminocaproic Acid] explode all trees

#43 (antifibrinolytic* or anti‐fibrinolytic* or antifibrinolysin* or antiplasmin* or plasmin inhibitor* or tranexamic or tranhexamic or cyclohexanecarboxylic acid* or amcha or t‐amcha or amca or transamin or amchafibrin or anvitoff or spotof or cyklokapron or femstrual or ugurol):ti,ab

#44 (AMCHA or amchafibrin or amikapron or amstat or antivoff or caprilon or cl65336 or cyclocapron or cyclokapron or cyklocapron or cyklokapron or exacyl or frenolyse or fibrinon or hemostan or hexacapron):ti,ab

#45 (hexakapron or kalnex or lysteda or rikaparin or ronex or theranex or tranexam or tranexanic or tranexic or "trans achma" or transexamic or trenaxin or TXA):ti,ab

#46 (fibrinolysis near/2 inhibitor*):ti,ab

#47 (Agretax or Bio‐Stat or Capiloc or Capitrax or Clip Inj or Clot‐XL or Clotawin‐T or Coastat or Cuti or Cymin or Dubatran or Espercil or Examic or Existat or Extam or Fibran or Gynae‐Pil or Hemstate or Kapron or Menogia or Monitex or Nestran or Nexamic or Nexi‐500 or Nexmeff or Nicolda or Nixa‐500 or Pause or Rheonex or Sylstep TX or Synostat or T‐nex or T Stat or T Stat or Tanmic or Temsyl‐T or Texakind or Texanis or Texapar or Texid or Thams or Tonopan or Traklot or Tramic or Tramix or Tranarest or Trance Inj or Tranecid or Tranee or Tranemic or Tranex or Tranexa or Tranfib or Tranlok or Transtat or Transys or Transcam or Tranxi or Trapic or Traxage or Traxamic or Traxyl or Trenaxa or Trexamic or Trim Inj or Tx‐1000 or Tx 500 or Wistran or X‐Tran or Xamic):ti,ab

#48 (ecapron or ekaprol or epsamon or epsicaprom or epsicapron or epsilcapramin or epsilon amino caproate or epsilon aminocaproate or epsilonaminocaproic or epsilonaminocapronsav or ethaaminocaproic or ethaaminocaproich or emocaprol or hepin or ipsilon or neocaprol or resplamin or tachostyptan):ti,ab

#49 (lederle or acikaprin or afibrin or amicar or caprocid or capracid or capramol or caprogel or caprolest or caprolisin* or caprolysin* or capromol or epsikapron or hemocaprol or caproamin or EACA or caprolest or capralense or hexalense or hamostat or hemocid):ti,ab

#50 (aminohexanoic or aminocaproic or aminohexanoic or amino caproic or amino‐caproic or amino‐n‐hexanoic):ti,ab

#51 #40 or #41 or #42 or #43 or #44 or #45 or #46 or #47 or #48 or #49 or #50

#52 MeSH descriptor: [Aprotinin] this term only

#53 (antagosan or antilysin* or aprotimbin or apronitin* or aprotinin* or bayer a128 or contrical or contrycal or contrykal or dilmintal or frey inhibitor or kontrycal or Kunitz inhibitor or gordox or haemoprot or kallikrein‐trypsin inactivator or iniprol or kontrikal or kontrykal or kunitz pancreatic trypsin inhibitor or midran or pulmin or tracylol or trascolan or trasilol or tra?ylol or traskolan or zymofren or pancreas antitrypsin or protinin or riker 52g or Rivilina zymofren):ti,ab

#54 #52 or #53

#55 MeSH descriptor: [Factor VIIa] this term only

#56 (factor viia or factor 7a or rfviia or fviia or novoseven* or novo seven* or aryoseven or acset or eptacog* or proconvertin):ti,ab

#57 (activated near/1 (factor seven or factor vii or rfvii or fvii)):ti,ab

#58 (factor seven or factor vii or factor 7):ti

#59 #55 or #56 or #57 or #58

#60 MeSH descriptor: [Fibrinogen] this term only

#61 ("fibrinogen concentrate" or "factor I" or Haemocomplettan* or Riastap* or Fibryga* or Fibryna*):ti,ab

#62 #60 or #61

#63 MeSH descriptor: [Deamino Arginine Vasopressin] this term only

#64 (desmopressin* or vasopressin deamino or D amino D arginine vasopressin or deamino 8 d arginine vasopressin or vasopressin desamino 8 arginine or desmotabs or DDAVP or desmogalen or adin or adiuretin or concentraid or d void or dav ritter or deamino 8 dextro arginine vasopressin or deamino 8d arginine vasopressin or deamino dextro arginine vasopressin or deaminovasopressin or defirin or defirin melt or desmirin pr desmomelt or desmopresina or desmospray or desmotab* or desurin or emosint or enupresol or minirin or minirinette or minirinmelt or minrin or minurin or miram or nictur or noctisson or nocturin or nocutil or nordurine or novidin or nucotil or octim or octostim or presinex or stimate or wetirin):ti,ab

#65 #63 or #64

#66 MeSH descriptor: [Factor XIII] explode all trees

#67 (factor xiii or fxiii or fibrin stabili?ing factor* or Tretten* or Catridecacog):ti,ab

#68 #66 or #67

#69 MeSH descriptor: [Tissue Adhesives] explode all trees

#70 MeSH descriptor: [Collagen] explode all trees and with qualifier(s): [therapeutic use ‐ TU]

#71 MeSH descriptor: [Thrombin] explode all trees and with qualifier(s): [therapeutic use ‐ TU]

#72 MeSH descriptor: [Gelatin] explode all trees and with qualifier(s): [therapeutic use ‐ TU]

#73 MeSH descriptor: [Gelatin Sponge, Absorbable] this term only

#74 ((fibrin* or collagen or cellulose or gelatin or gel or thrombin* or albumin or hemostatic* or haemostatic*) next (glu* or seal* or adhesive* or topical* or local* or matrix or matrices or spong* or fleece* or foam* or scaffold* or patch* or sheet* or bandag* or aerosol* or dressing* or paste or powder*)):ti,ab

#75 ((nonfibrin* or non‐fibrin* or synthetic* or non‐biological* or nonbiological* or biological*) near/3 (glue* or seal* or adhesive*)):ti,ab

#76 (surgical* near/3 (glue* or sealant* or adhesive*)):ti,ab

#77 ((fibrin* or collagen or cellulose or gelatin or thrombin) near/3 (hemosta* or haemosta*)):ti,ab

#78 (8Y or Aafact or Actif‐VIII or Advate or Artiss or Bioglue or Biocol or Collaseal or Omrixil or Transglutine or Raplixa or Evarrest or Aleviate or Alphanate or Amofil or Beriate or Beriplast or Biostate or Bolheal or Cluvot or Conco‐Eight‐HT or Crosseel or Crosseal or Crosseight or Emoclot or Evarrest or Evicel or Factane or Fanhdi or Fibrogammin P or Green VIII or Green VIII Factor or Greengene or Greenmono or Greenplast or Haemate or Haemate P or Haemate P or Haemate P500 or Haemate‐P or Haemoctin or Haemoctin SDH or Haemoctin‐SDH or Hemaseel or Hemaseal or Hemofil M or Hemoraas or Humaclot or Humafactor‐8 or Humate‐P or Immunate or Innovate or Koate or Koate‐DVI or Kogenate Bayer or Kogenate FS or Monoclate‐P or NovoThirteen or Octafil or Octanate or Octanate or Optivate or Quixil or Talate or Tisseel or Tisseal or Tissel or Tissucol or Tricos or Vivostat or Voncento or Wilate or Wilnativ or Wilstart or Xyntha):ti,ab

#79 (Glubran or Gluetiss or Ifabond or Indermil or LiquiBand or TissuGlu or Evithrom or Floseal or Hemopatch or Gel‐Flow or Gelfoam or Gelfilm or Recothrom or Surgifoam or Surgiflo* or "rh Thrombin" or Thrombi‐Gel or Thrombi‐Pad or Thrombin‐JMI or Thrombinar or Thrombogen or Thrombostat):ti,ab

#80 (porcine gelatin or bovine collagen or bovine gelatin or nu‐knit or arista or hemostase or vita sure or thrombin‐jmi or thrombinjmi or avicel or vivagel or lyostypt or tabotamp or arterx or omnex or veriset):ti,ab

#81 (polysaccharide next (sphere* or hemostatic powder)):ti,ab

#82 MeSH descriptor: [Chitosan] this term only

#83 MeSH descriptor: [Polyethylene Glycols] this term only and with qualifier(s): [therapeutic use ‐ TU]

#84 MeSH descriptor: [Hydrogel, Polyethylene Glycol Dimethacrylate] explode all trees and with qualifier(s): [therapeutic use ‐ TU]

#85 MeSH descriptor: [Polyurethanes] explode all trees and with qualifier(s): [pharmacology ‐ PD, adverse effects ‐ AE, toxicity ‐ TO, administration & dosage ‐ AD, therapeutic use ‐ TU]

#86 ((polymer‐derived elastic* or polymer tissue adhesive* or elastic hydrogel* or glutaraldehyde or PEG‐based or polyurethane‐based tissue or polyethylene glycol* or polyvinyl alcohol‐based tissue or PVA‐based tissue or natural biopolymer* or polypeptide‐based or protein‐based or polysaccharide‐based or chitosan or poliglusam or cyanoacrylic or cyanoacrylate or cyacrin or dextran‐based or chondroitin sulfate‐based or mussel‐inspired elastic* or glycol hydrogel or polymer‐based) next (glu* or seal* or adhesive* or topical* or local* or matrix or matrices or spong* or fleece* or foam* or scaffold* or patch* or sheet* or bandag* or aerosol* or dressing* or paste* or powder*)):ti,ab

#87 MeSH descriptor: [Cellulose, Oxidized] this term only

#88 (absorbable cellulose or resorbable cellulose or oxidi?ed cellulose or carboxycellulose or oxycellulose or cellulosic acid or oxycel or oxidi?ed regenerated cellulose):ti,ab

#89 (BioGlue or Progel or Duraseal or Coseal or FocalSeal or ADAL‐1 or AdvaSeal or Pleuraseal or Angio‐Seal or Avitene or Instat or Helitene or Helistat or TDM‐621 or Dermabond or Tissueseal or PolyStat or Raplixa or Spongostan or Surgicel or Surgilux or Tachosil or Traumstem):ti,ab

#90 (collagen‐thrombin or thrombin‐collagen or gelatin‐fibrinogen or fibrinogen‐gelatin or gelatin‐thrombin or thrombin‐gelatin or fibrinogen‐thrombin or thrombin‐fibrinogen or collagen‐fibrinogen or fibrinogen‐collagen or microfibrillar collagen or CoStasis or "GRF Glue" or GR‐Dial or Algosterile or TraumaStat or HemCon or ChitoFlex or Celox or QuikClot or WoundStat or Vitagel or TachSeal or TachoComb or Cryoseal):ti,ab

#91 #69 or #70 or #71 or #72 or #73 or #74 or #75 or #76 or #77 or #78 or #79 or #80 or #81 or #82 or #83 or #84 or #85 or #86 or #87 or #88 or #89 or #90

#92 MeSH descriptor: [Waxes] explode all trees

#93 (bonewax* or bone wax* or bone putty or hemasorb or ostene):ti,ab

#94 #92 or #93

#95 MeSH descriptor: [Blood Coagulation Factors] this term only

#96 (prothrombin near/5 (complex* or concentrate*))

#97 (PCC* or 3F‐PCC* or 4F‐PCC* or Beriplex* or Feiba* or Autoplex* or Ocplex* or Octaplex* or Kcentra* or Cofact or Prothrombinex* or "Proplex‐T" or Prothroraas* or Haemosolvex* or Prothromplex* or "HT Defix" or Facnyne* or Kaskadil* or Kedcom* or Confidex* or PPSB or Profil?ine* or Pronativ* or Proplex* or Prothar* or ProthoRAAS* or Protromplex* or "Pushu Laishi" or "Uman Complex")

#98 #95 or #96 or #97

#99 (((haemosta* or hemosta* or antihaemorrhag* or antihemorrhag* or anti haemorrhag* or anti‐hemorrhag*) next (drug* or agent* or treat* or therap*)) or ((coagulat* or clotting) next factor*)):ti,ab

#100 #51 or #54 or #59 or #62 or #65 or #68 or #91 or #94 or #98 or #99

#101 #39 and #100  

#### MEDLINE (OvidSP)

1. exp Femoral Fractures/

2. Ankle Fractures/

3. Humeral Fractures/

4. Osteoporotic Fractures/

5. Periprosthetic Fractures/

6. exp Shoulder Fractures/

7. Tibial Fractures/

8. exp Ulna Fractures/

9. Radius Fractures/

10. Fractures, Bone/

11. ((pelvi* or sacrum or coccyx or ischium or pubis or pubic or ilium or tailbone or diaphys* or epiphys* or metaphys* or acetabulum or acetabular or femor* or femur* or hip* or thigh* or tibia* or fibula* or intertrochanteric or subtrochanteric or petrochanteric or intracapsular or subcapsular or subcapital or osteoporo* or osteoarthritis or orthop?edic) adj6 (fracture* or break* or broke* or trauma* or injur* or surg* or operat* or repair* or reconstruct* or fixation* or implant* or prosthes* or "plate and screw" or "plate and screws" or intramedullary nail*)).tw,kf.

12. ((long bone* or long‐bone* or humerus or humeral or upper arm* or shoulder* or clavicle* or clavicula* or collar bone* or ankle* or pilon or lower leg* or calf* or knee* or tibiofibular or menisci or meniscus or femoropatellar or patellofemoral or radial or radius or ulna or forearm* or elbow*) adj6 (fracture* or break* or broke* or trauma* or injur* or surg* or operat* or repair* or reconstruct* or fixation* or implant* or prosthes* or "plate and screw" or "plate and screws" or intramedullary nail*)).tw,kf.

13. ((malleol* or talus or trochanteric or crural or crus or olecranon or antebrachial or monteggi* or bankart) adj6 (fracture* or break* or broke* or trauma* or injur* or surg* or operat* or repair* or reconstruct* or fixation* or implant* or prosthes* or "plate and screw" or "plate and screws" or intramedullary nail*)).tw,kf.

14. ((wrist* or capitate or hamtae or lunate or carpal or metacarpal or pisiform or scaphoid or trapezium or triquetral) adj6 (fracture* or break* or broke* or trauma* or injur* or surg* or operat* or repair* or reconstruct* or fixation* or implant* or prosthes* or "plate and screw" or "plate and screws" or intramedullary nail*)).tw,kf.

15. ((peri‐implant or periprosthetic) adj1 fracture*).tw,kf.

16. exp Pelvic Bones/in, su

17. exp Leg Bones/in, su

18. exp Arm Bones/in, su

19. Clavicle/in, su

20. "Bones of Upper Extremity"/in, su or "Bones of Lower Extremity"/in, su

21. exp Hip Joint/su or Shoulder Joint/su or exp Knee Joint/su

22. Ankle Joint/su or Elbow Joint/in, su

23. exp Hip Injuries/su or exp Knee Injuries/su

24. exp Arm Injuries/su or exp Shoulder Injuries/su

25. Lower Extremity/in, su or Hip/su or Thigh/su or Leg/su or Knee/su

26. Upper Extremity/in, su or Arm/su or Elbow/su or Forearm/su or Shoulder/su

27. ((hip* or shoulder* or knee*) adj5 (replac* or arthroplast* or hemiarthroplast* or hemi‐arthroplast*)).mp.

28. exp Leg Bones/ or exp Arm Bones/ or Clavicle/ or exp Humerus/ or exp Pelvic Bones/ or exp Femur/ or Tibia/ or Fibula/ or "Bones of Upper Extremity"/ or "Bones of Lower Extremity"/

29. exp Fracture Fixation/ or (trauma* or fracture* or injur* or surg* or operat* or repair* or fixation).ti.

30. 28 and 29

31. (or/1‐27) or 30

32. Antifibrinolytic Agents/

33. Tranexamic Acid/

34. Aminocaproic Acid/

35. (antifibrinolytic* or anti‐fibrinolytic* or antifibrinolysin* or antiplasmin* or plasmin inhibitor* or tranexamic or tranhexamic or cyclohexanecarboxylic acid* or amcha or trans‐4‐aminomethyl‐cyclohexanecarboxylic acid* or t‐amcha or amca or "kabi 2161" or transamin or amchafibrin or anvitoff or spotof or cyklokapron or cyclo‐F or femstrual or ugurol or aminomethylcyclohexanecarbonic acid or aminomethylcyclohexanecarboxylic acid or AMCHA or amchafibrin or amikapron or aminomethyl cyclohexane carboxylic acid or aminomethyl cyclohexanecarboxylic acid or aminomethylcyclohexane carbonic acid or aminomethylcyclohexane carboxylic acid or aminomethylcyclohexanecarbonic acid or aminomethylcyclohexanecarboxylic acid or aminomethylcyclohexanocarboxylic acid or aminomethylcyclohexanoic acid or amstat or antivoff or caprilon or cl?65336 or cl65336 or cyclocapron or cyclokapron or cyklocapron or cyklokapron or exacyl or frenolyse or fibrinon or hemostan or hexacapron or hexakapron or kalnex or lysteda or rikaparin or ronex or theranex or tranexam or tranexanic or tranexic or trans achma or transexamic or trenaxin or TXA or (fibrinolysis adj2 inhibitor*)).tw,kf.

36. (Agretax or Bio‐Stat or Capiloc or Capitrax or Clip Inj or Clot‐XL or Clotawin‐T or Coastat or Cuti or Cymin or Dubatran or Espercil or Examic or Existat or Extam or Fibran or Gynae‐Pil or Hemstate or Kapron or Menogia or Monitex or Nestran or Nexamic or Nexi‐500 or Nexmeff or Nicolda or Nixa‐500 or Pause or Rheonex or Sylstep TX or Synostat or T‐nex or T Stat or T Stat or Tanmic or Temsyl‐T or Texakind or Texanis or Texapar or Texid or Thams or Tonopan or Traklot or Tramic or Tramix or Tranarest or Trance Inj or Tranecid or Tranee or Tranemic or Tranex or Tranexa or Tranfib or Tranlok or Transtat or Transys or Transcam or Tranxi or Trapic or Traxage or Traxamic or Traxyl or Trenaxa or Trexamic or Trim Inj or Tx‐1000 or Tx 500 or Wistran or X‐Tran or Xamic).tw,kf.

37. (6‐aminohexanoic or amino?caproic or amino?hexanoic or amino caproic or amino‐caproic or amino‐n‐hexanoic or cy‐116 or cy116 or lederle or acikaprin or afibrin or amicar or caprocid or capracid or capramol or caprogel or caprolest or caprolisin* or caprolysin* or capromol or epsikapron or hemocaprol or caproamin or EACA or caprolest or capralense or hexalense or hamostat or hemocid or cl 10304 or cl10304 or ecapron or ekaprol or epsamon or epsicaprom or epsicapron or epsilcapramin or epsilon amino caproate or epsilon aminocaproate or epsilonaminocaproic or epsilonaminocapronsav or etha?aminocaproic or ethaaminocaproich or emocaprol or hepin or ipsilon or jd?177or neocaprol or nsc?26154 or resplamin or tachostyptan).tw,kf.

38. or/32‐37

39. Aprotinin/

40. (antagosan or antilysin* or aprotimbin or apronitin* or aprotinin* or bayer a128 or contrical or contrycal or contrykal or dilmintal or frey inhibitor or kontrycal or Kunitz inhibitor or gordox or haemoprot or kallikrein‐trypsin inactivator).tw,kf.

41. (iniprol or kontrikal or kontrykal or kunitz pancreatic trypsin inhibitor or midran or pulmin or tracylol or trascolan or trasilol or tra?ylol or traskolan or zymofren or pancreas antitrypsin or protinin or riker 52g or Rivilina zymofren).tw,kf.

42. or/39‐41

43. Factor VIIa/

44. (factor viia or factor 7a or rfviia or fviia or novoseven* or novo seven* or aryoseven or acset or eptacog* or proconvertin).tw,kf.

45. ((activated adj2 factor seven) or (activated adj2 factor vii) or (activated adj3 rfvii) or (activated adj2 fvii)).tw,kf.

46. (factor seven or factor vii or factor 7).ti.

47. 43 or 44 or 45 or 46

48. Fibrinogen/ad, ae, de, sd, tu, th, to

49. *Fibrinogen/

50. (fibrinogen concentrate* or factor I or Haemocomplettan* or Riastap* or Fibryga* or Fibryna*).tw,kf.

51. 48 or 49 or 50

52. Deamino Arginine Vasopressin/

53. (desmopressin* or vasopressin deamino or D‐amino D‐arginine vasopressin or deamino‐8‐d‐arginine vasopressin or vasopressin 1‐desamino‐8‐arginine or desmotabs or DDAVP or desmogalen or adin or adiuretin or concentraid or d‐void or dav ritter or deamino 8 dextro arginine vasopressin or deamino 8d arginine vasopressin or deamino dextro arginine vasopressin or deaminovasopressin or defirin or defirin melt or desmirin or desmomelt or desmopresina or desmospray or desmotab* or desurin or emosint or enupresol or minirin or minirinette or minirinmelt or minrin or minurin or miram or nictur or noctisson or nocturin or nocutil or nordurine or novidin or nucotil or octim or octostim or presinex or stimate or wetirin).tw,kf.

54. 52 or 53

55. exp Factor XIII/

56. (factor XIII or fXIII or fibrin stabili?ing factor* or Tretten* or Catridecacog).tw,kf.

57. 55 or 56

58. exp Tissue Adhesives/

59. *Adhesives/

60. Collagen/tu

61. Thrombin/tu

62. Gelatin/tu

63. Gelatin Sponge, Absorbable/

64. ((fibrin* or collagen or cellulose or gelatin or gel or thrombin* or albumin or hemostatic* or haemostatic*) adj3 (glu* or seal* or adhesive* or topical* or local* or matrix or matrices or spong* or fleece* or foam* or scaffold* or patch* or sheet* or bandag* or aerosol* or dressing* or paste or powder*)).tw,kf.

65. ((nonfibrin* or non‐fibrin* or synthetic* or non‐biological* or nonbiological* or biological*) adj3 (glue* or seal* or adhesive*)).tw,kf.

66. (surgical* adj3 (glue* or sealant* or adhesive*)).tw,kf.

67. ((fibrin* or collagen or cellulose or gelatin or thrombin) adj3 (hemosta* or haemosta*)).tw,kf.

68. (8Y or Aafact or Actif‐VIII or Advate or Artiss or Bioglue or Biocol or Collaseal or Omrixil or Transglutine or Raplixa or Evarrest or Aleviate or Alphanate or Amofil or Beriate or Beriplast or Biostate or Bolheal or Cluvot or Conco‐Eight‐HT or Crosseel or Crosseal or Crosseight or Emoclot or Evarrest or Evicel or Factane or Fanhdi or Fibrogammin P or Green VIII or Green VIII Factor or Greengene or Greenmono or Greenplast or Haemate or Haemate P or Haemate P or Haemate P500 or Haemate‐P or Haemoctin or Haemoctin SDH or Haemoctin‐SDH or Hemaseel or Hemaseal or Hemofil M or Hemoraas or Humaclot or Humafactor‐8 or Humate‐P or Immunate or Innovate or Koate or Koate‐DVI or Kogenate Bayer or Kogenate FS or Monoclate‐P or NovoThirteen or Octafil or Octanate or Octanate or Optivate or Quixil or Talate or Tisseel or Tisseal or Tissel or Tissucol or Tricos or Vivostat or Voncento or Wilate or Wilnativ or Wilstart or Xyntha).tw,kf.

69. (Glubran or Gluetiss or Ifabond or Indermil or LiquiBand or TissuGlu).tw,kf.

70. (Evithrom or Floseal or Hemopatch or Gel‐Flow or Gelfoam or Gelfilm or Recothrom or Surgifoam or Surgiflo* or "rh Thrombin" or Thrombi‐Gel or Thrombi‐Pad or Thrombin‐JMI or Thrombinar or Thrombogen or Thrombostat).tw,kf.

71. (porcine gelatin or bovine collagen or bovine gelatin or nu‐knit or arista or hemostase or vita sure or thrombin‐jmi or thrombinjmi or avicel or vivagel or lyostypt or tabotamp or arterx or omnex or veriset).tw,kf.

72. (polysaccharide adj (sphere* or hemostatic powder)).tw,kf.

73. *Chitosan/

74. *Polyethylene Glycols/

75. *Hydrogel, Polyethylene Glycol Dimethacrylate/

76. Polyurethanes/ad, ae, pd, tu, to

77. ((polymer‐derived elastic* or polymer tissue adhesive* or elastic hydrogel* or glutaraldehyde or PEG‐based or polyurethane‐based tissue or polyethylene glycol* or polyvinyl alcohol‐based tissue or PVA‐based tissue or natural biopolymer* or polypeptide‐based or protein‐based or polysaccharide‐based or chitosan or poliglusam or cyanoacrylic or cyanoacrylate or cyacrin or dextran‐based or chondroitin sulfate‐based or mussel‐inspired elastic* or glycol hydrogel or polymer‐based) adj3 (glu* or seal* or adhesive* or topical* or local* or matrix or matrices or spong* or fleece* or foam* or scaffold* or patch* or sheet* or bandag* or aerosol* or dressing* or paste* or powder*)).tw,kf.

78. Cellulose, Oxidized/

79. (absorbable cellulose or resorbable cellulose or oxidi?ed cellulose or carboxycellulose or oxycellulose or cellulosic acid or oxycel or oxidi?ed regenerated cellulose).tw,kf.

80. (BioGlue or Progel or Duraseal or Coseal or FocalSeal or ADAL‐1 or AdvaSeal or Pleuraseal or Angio‐Seal or Avitene or Instat or Helitene or Helistat or TDM‐621 or Dermabond or Tissueseal or PolyStat or Raplixa or Spongostan or Surgicel or Surgilux or Tachosil or Traumstem).tw,kf.

81. (collagen‐thrombin or thrombin‐collagen or gelatin‐fibrinogen or fibrinogen‐gelatin or gelatin‐thrombin or thrombin‐gelatin or fibrinogen‐thrombin or thrombin‐fibrinogen or collagen‐fibrinogen or fibrinogen‐collagen or microfibrillar collagen or CoStasis or "GRF Glue" or GR‐Dial or Algosterile or TraumaStat or HemCon or ChitoFlex or Celox or QuikClot or WoundStat or Vitagel or TachSeal or TachoComb or Cryoseal).tw,kf.

82. or/58‐81

83. exp Waxes/

84. (bonewax* or bone wax* or bone putty or hemasorb or ostene).tw,kf.

85. 83 or 84

86. (((haemosta* or hemosta* or antihaemorrhag* or antihemorrhag* or anti haemorrhag* or anti‐hemorrhag*) adj5 (drug* or agent* or treat* or therap*)) or ((coagulat* or clotting) adj factor*)).tw,kf.

87. 38 or 42 or 47 or 51 or 54 or 57 or 82 or 85 or 86

88. 31 and 87

89. Systematic Review.pt.

90. Meta‐Analysis.pt.

91. ((meta analy* or metaanaly*) and (trials or studies)).ab.

92. (meta analy* or metaanaly* or evidence‐based).ti.

93. ((systematic* or evidence‐based) adj2 (review* or overview*)).tw,kf.

94. (evidence synthes* or cochrane or medline or pubmed or embase or cinahl or cinhal or lilacs or "web of science" or science citation index or scopus or search terms or literature search or electronic search* or comprehensive search* or systematic search* or published articles or search strateg* or reference list* or bibliograph* or handsearch* or hand search* or manual* search*).ab.

95. Cochrane Database of systematic reviews.jn.

96. (additional adj (papers or articles or sources)).ab.

97. ((electronic* or online) adj (sources or resources or databases)).ab.

98. (relevant adj (journals or articles)).ab.

99. or/89‐98

100. Review.pt.

101. exp Randomized Controlled Trials as Topic/

102. selection criteria.ab. or critical appraisal.tw.

103. (data adj (abstract* or extract* or analys*)).ab.

104. exp Randomized Controlled Trial/

105. or/101‐104

106. 100 and 105

107. 99 or 106

108. exp Randomized Controlled Trial/

109. Controlled Clinical Trial/

110. (placebo or randomly or groups).ab.

111. (randomi* or trial).tw,kf.

112. exp Clinical Trial as Topic/

113. 107 or 108 or 109 or 110 or 111 or 112

114. exp animals/not humans/

115. 113 not 114

116. 88 and 115  

#### PubMed

(fracture*[TIAB] OR break*[TIAB] OR broke*[TIAB] OR trauma[TIAB] OR traumatic[TIAB] OR injury[TIAB] OR injured[TIAB] OR injuries[TIAB] OR repair*[TIAB] OR reconstruct*[TIAB] OR fixation*[TIAB] OR implant*[TIAB] OR prosthes*[TIAB] OR "plate and screw"[TIAB] OR "plate and screws"[TIAB] OR "intramedullary nail"[TIAB] OR surgery[TIAB] OR surgical[TIAB] OR operation*[TIAB] OR operate*[TIAB] OR operating[TIAB]) AND (long bone[TIAB] OR pelvic[TIAB] OR ischium[TIAB] OR pubis[TIAB] OR pubic[TIAB] OR ilium[TIAB] OR acetabular[TIAB] OR acetabulum[TIAB] OR femoral[TIAB] OR femur[TIAB] OR hip[TIAB] OR knee[TIAB] OR shoulder[TIAB] OR clavicle[TIAB] OR collar bone[TIAB] OR diaphysis[TIAB] OR epiphysis[TIAB] OR metaphysis[TIAB] OR humerus[TIAB] OR humeral[TIAB] OR tibia[TIAB] OR tibial[TIAB] OR fibula[TIAB] OR ankle[TIAB] OR pilon[TIAB] OR ulna[TIAB] OR radius[TIAB] OR radial[TIAB] OR elbow[TIAB] OR intertrochanteric[TIAB] OR subtrochanteric[TIAB] OR petrochanteric[TIAB] OR intracapsular[TIAB] OR subcapsular[TIAB] OR osteoporosis[TIAB] OR osteoporotic[TIAB] OR osteoarthritis[TIAB] OR orthopedic trauma[TIAB] OR surgical fixation[TIAB] OR hemiarthroplasty[TIAB] OR arthroplasty[TIAB] OR periprosthetic[TIAB]) AND (hemostatic[TIAB] OR antifibrinolytic[TIAB] OR tranexamic[TIAB] OR EACA[TIAB] OR aminocaproic[TIAB] OR aprotinin[TIAB] OR desmopressin[TIAB] OR DDAVP[TIAB] OR factor viia[TIAB] OR novoseven[TIAB] OR aryoseven[TIAB] OR fibrinogen[TIAB] OR haemocomplettan[TIAB] OR Riastap[TIAB] OR Fibryna[TIAB] OR Fibryga[TIAB] OR factor XIII[TIAB] OR Tretten[TIAB] OR sealant[TIAB] OR adhesive[TIAB] OR collagen[TIAB] OR cellulose[TIAB] OR gelatin[TIAB] OR glue[TIAB] OR matrix[TIAB] OR sponge[TIAB] OR fleece[TIAB] OR foam[TIAB] OR scaffold[TIAB] OR patch[TIAB] OR sheet[TIAB] OR gelfoam[TIAB] OR chitosan[TIAB] OR hydrogel[TIAB] OR polyethylene glycol[TIAB] OR tachocomb[TIAB] OR BioGlue[TIAB] OR Surgicel[TIAB] OR Veriset[TIAB] OR Evithrom[TIAB] OR Floseal[TIAB] OR Tachosil[TIAB] OR Cryoseal[TIAB] OR Hemopatch[TIAB] OR Progel[TIAB] OR Duraseal[TIAB] OR Coseal[TIAB] OR FocalSeal[TIAB] OR Algosterile[TIAB] OR TraumaStat[TIAB] OR HemCon[TIAB] OR ChitoFlex[TIAB] OR Celox[TIAB] OR QuikClot[TIAB] OR WoundStat[TIAB] OR Vitagel[TIAB] OR TachSeal[TIAB] OR bonewax[TIAB] OR hemasorb[TIAB] OR ostene[TIAB] OR iniprol[TIAB] OR kontrikal[TIAB] OR CloSys[TIAB] OR Glubran[TIAB] OR Gluetiss[TIAB] OR Ifabond[TIAB] OR Indermil[TIAB] OR LiquiBand[TIAB] OR Octafil[TIAB] OR Octanate[TIAB] OR Optivate[TIAB] OR Quixil[TIAB] OR Tisseel[TIAB] OR Tissucol[TIAB] OR TissuGlu[TIAB] OR Thrombi‐Gel[TIAB] OR Vivostat[TIAB] OR Voncento[TIAB] OR Wilate[TIAB] OR Wilnativ[TIAB] OR Wilstart[TIAB]) AND (random* OR blind* OR "control group" OR placebo* OR controlled OR trial* OR "systematic review" OR "meta‐analysis" OR metaanalysis OR "evidence synthesis" OR "literature search" OR medline OR pubmed OR cochrane OR embase) AND (publisher[sb] OR inprocess[sb] OR pubmednotmedline[sb]))

#### Embase (OvidSP)

1. exp leg fracture/

2. exp arm fracture/

3. exp pelvis fracture/

4. clavicle fracture/

5. fragility fracture/

6. periprosthetic fracture/

7. ((pelvi* or sacrum or coccyx or ischium or pubis or pubic or ilium or tailbone or diaphys* or epiphys* or metaphys* or acetabulum or acetabular or femor* or femur* or hip* or thigh* or tibia* or fibula* or intertrochanteric or subtrochanteric or petrochanteric or intracapsular or subcapsular or subcapital or osteoporo* or osteoarthritis or orthop?edic) adj6 (fracture* or break* or broke* or trauma* or injur* or surg* or operat* or repair* or reconstruct* or fixation* or implant* or prosthes* or "plate and screw" or "plate and screws" or intramedullary nail*)).tw,kw.

8. ((long bone* or long‐bone* or humerus or humeral or upper arm* or shoulder* or clavicle* or clavicula* or collar bone* or ankle* or pilon or lower leg* or calf* or knee* or tibiofibular or menisci or meniscus or femoropatellar or patellofemoral or radial or radius or ulna or forearm* or elbow*) adj6 (fracture* or break* or broke* or trauma* or injur* or surg* or operat* or repair* or reconstruct* or fixation* or implant* or prosthes* or "plate and screw" or "plate and screws" or intramedullary nail*)).tw,kw.

9. ((malleol* or talus or trochanteric or crural or crus or olecranon or antebrachial or monteggi* or bankart) adj6 (fracture* or break* or broke* or trauma* or injur* or surg* or operat* or repair* or reconstruct* or fixation* or implant* or prosthes* or "plate and screw" or "plate and screws" or intramedullary nail*)).tw,kw.

10. ((wrist* or capitate or hamtae or lunate or carpal or metacarpal or pisiform or scaphoid or trapezium or triquetral) adj6 (fracture* or break* or broke* or trauma* or injur* or surg* or operat* or repair* or reconstruct* or fixation* or implant* or prosthes* or "plate and screw" or "plate and screws" or intramedullary nail*)).tw,kw.

11. ((peri‐implant or periprosthetic) adj1 fracture*).tw,kw.

12. exp long bone/su

13. exp pelvic girdle/su [Surgery]

14. exp "bones of the leg and foot"/su [Surgery]

15. exp "bones of the arm and hand"/su [Surgery]

16. exp fibula/su [Surgery]

17. exp femur/su [Surgery]

18. exp tibia/su

19. exp shoulder/su

20. exp knee/su

21. exp hip/su

22. exp elbow/su

23. exp ankle/su

24. exp humerus/su [Surgery]

25. exp hip injury/su [Surgery]

26. exp knee injury/su [Surgery]

27. exp arm injury/su [Surgery]

28. exp leg injury/su [Surgery]

29. exp pelvis injury/su [Surgery]

30. exp lower limb/su

31. exp upper limb/su

32. ((hip* or shoulder* or knee*) adj5 (replac* or arthroplast* or hemiarthroplast* or hemi‐arthroplast*)).mp.

33. exp leg bone/

34. exp arm bone/

35. exp pelvic girdle/

36. exp long bone/

37. exp shoulder girdle/

38. 33 or 34 or 35 or 36 or 37

39. exp fracture treatment/

40. (trauma* or fracture* or injur* or surg* or operat* or repair* or fixation).ti.

41. 39 or 40

42. 38 and 41

43. (or/1‐32) or 42

44. Antifibrinolytic Agent/

45. Tranexamic Acid/

46. Aminocaproic Acid/

47. (antifibrinolytic* or anti‐fibrinolytic* or antifibrinolysin* or antiplasmin* or plasmin inhibitor* or tranexamic or tranhexamic or cyclohexanecarboxylic acid* or amcha or trans‐4‐aminomethyl‐cyclohexanecarboxylic acid* or t‐amcha or amca or "kabi 2161" or transamin or amchafibrin or anvitoff or spotof or cyklokapron or cyclo‐F or femstrual or ugurol or aminomethylcyclohexanecarbonic acid or aminomethylcyclohexanecarboxylic acid or AMCHA or amchafibrin or amikapron or aminomethyl cyclohexane carboxylic acid or aminomethyl cyclohexanecarboxylic acid or aminomethylcyclohexane carbonic acid or aminomethylcyclohexane carboxylic acid or aminomethylcyclohexanecarbonic acid or aminomethylcyclohexanecarboxylic acid or aminomethylcyclohexanocarboxylic acid or aminomethylcyclohexanoic acid or amstat or antivoff or caprilon or cl?65336 or cl65336 or cyclocapron or cyclokapron or cyklocapron or cyklokapron or exacyl or frenolyse or fibrinon or hemostan or hexacapron or hexakapron or kalnex or lysteda or rikaparin or ronex or theranex or tranexam or tranexanic or tranexic or trans achma or transexamic or trenaxin or TXA or (fibrinolysis adj2 inhibitor*)).tw,kw.

48. (Agretax or Bio‐Stat or Capiloc or Capitrax or Clip Inj or Clot‐XL or Clotawin‐T or Coastat or Cuti or Cymin or Dubatran or Espercil or Examic or Existat or Extam or Fibran or Gynae‐Pil or Hemstate or Kapron or Menogia or Monitex or Nestran or Nexamic or Nexi‐500 or Nexmeff or Nicolda or Nixa‐500 or Pause or Rheonex or Sylstep TX or Synostat or T‐nex or T Stat or T Stat or Tanmic or Temsyl‐T or Texakind or Texanis or Texapar or Texid or Thams or Tonopan or Traklot or Tramic or Tramix or Tranarest or Trance Inj or Tranecid or Tranee or Tranemic or Tranex or Tranexa or Tranfib or Tranlok or Transtat or Transys or Transcam or Tranxi or Trapic or Traxage or Traxamic or Traxyl or Trenaxa or Trexamic or Trim Inj or Tx‐1000 or Tx 500 or Wistran or X‐Tran or Xamic).tw,kw.

49. (6‐aminohexanoic or amino?caproic or amino?hexanoic or amino caproic or amino‐caproic or amino‐n‐hexanoic or cy‐116 or cy116 or lederle or acikaprin or afibrin or amicar or caprocid or capracid or capramol or caprogel or caprolest or caprolisin* or caprolysin* or capromol or epsikapron or hemocaprol or caproamin or EACA or caprolest or capralense or hexalense or hamostat or hemocid or cl 10304 or cl10304 or ecapron or ekaprol or epsamon or epsicaprom or epsicapron or epsilcapramin or epsilon amino caproate or epsilon aminocaproate or epsilonaminocaproic or epsilonaminocapronsav or etha?aminocaproic or ethaaminocaproich or emocaprol or hepin or ipsilon or jd?177or neocaprol or nsc?26154 or resplamin or tachostyptan).tw,kw.

50. or/44‐49

51. Aprotinin/

52. (antagosan or antilysin* or aprotimbin or apronitin* or aprotinin* or bayer a128 or contrical or contrycal or contrykal or dilmintal or frey inhibitor or kontrycal or Kunitz inhibitor or gordox or haemoprot or kallikrein‐trypsin inactivator).tw,kw.

53. (iniprol or kontrikal or kontrykal or kunitz pancreatic trypsin inhibitor or midran or pulmin or tracylol or trascolan or trasilol or tra?ylol or traskolan or zymofren or pancreas antitrypsin or protinin or riker 52g or Rivilina zymofren).tw,kw.

54. or/51‐53

55. Blood Clotting Factor 7a/

56. (factor viia or factor 7a or rfviia or fviia or novoseven* or novo seven* or aryoseven or acset or eptacog* or proconvertin).tw,kw.

57. ((activated adj2 factor seven) or (activated adj2 factor vii) or (activated adj3 rfvii) or (activated adj2 fvii)).tw,kw.

58. (factor seven or factor vii or factor 7).ti.

59. 55 or 56 or 57 or 58

60. Fibrinogen Concentrate/

61. (fibrinogen concentrate* or factor I or Haemocomplettan* or Riastap* or Fibryga* or Fibryna*).tw,kw.

62. 60 or 61

63. Desmopressin/

64. (desmopressin* or vasopressin deamino or D‐amino D‐arginine vasopressin or deamino‐8‐d‐arginine vasopressin or vasopressin 1‐desamino‐8‐arginine or desmotabs or DDAVP or desmogalen or adin or adiuretin or concentraid or d‐void or dav ritter or deamino 8 dextro arginine vasopressin or deamino 8d arginine vasopressin or deamino dextro arginine vasopressin or deaminovasopressin or defirin or defirin melt or desmirin or desmomelt or desmopresina or desmospray or desmotab* or desurin or emosint or enupresol or minirin or minirinette or minirinmelt or minrin or minurin or miram or nictur or noctisson or nocturin or nocutil or nordurine or novidin or nucotil or octim or octostim or presinex or stimate or wetirin).tw,kw.

65. 63 or 64

66. Blood Clotting Factor 13/

67. (factor xiii or fxiii or fibrin stabili?ing factor* or Tretten* or Catridecacog).tw,kw.

68. 66 or 67

69. exp Tissue Adhesive/

70. *Adhesive Agent/

71. *Hemostatic Agent/

72. ((fibrin* or collagen or cellulose or gelatin or gel or thrombin* or albumin or hemostatic* or haemostatic*) adj3 (glu* or seal* or adhesive* or topical* or local* or matrix or matrices or spong* or fleece* or foam* or scaffold* or patch* or sheet* or bandag* or aerosol* or dressing* or paste or powder*)).tw,kw.

73. ((nonfibrin* or non‐fibrin* or synthetic* or non‐biological* or nonbiological* or biological*) adj3 (glue* or seal* or adhesive*)).tw,kw.

74. (surgical* adj3 (glue* or sealant* or adhesive*)).tw,kw.

75. ((fibrin* or collagen or cellulose or gelatin or thrombin) adj3 (hemosta* or haemosta*)).tw,kw.

76. (8Y or Aafact or Actif‐VIII or Advate or Artiss or Raplixa or Evarrest or Aleviate or Alphanate or Amofil or Beriate or Beriplast or Biostate or Bolheal or Cluvot or Conco‐Eight‐HT or Crosseel or Crosseal or Crosseight or Emoclot or Evarrest or Evicel or Factane or Fanhdi or Fibrogammin P or Green VIII or Green VIII Factor or Greengene or Greenmono or Greenplast or Haemate or Haemate P or Haemate P or Haemate P500 or Haemate‐P or Haemoctin or Haemoctin SDH or Haemoctin‐SDH or Hemaseel or Hemaseal or Hemofil M or Hemoraas or Humaclot or Humafactor‐8 or Humate‐P or Immunate or Innovate or Koate or Koate‐DVI or Kogenate Bayer or Kogenate FS or Monoclate‐P or NovoThirteen or Octafil or Octanate or Octanate or Optivate or Quixil or Talate or Tisseel or Tisseal or Tissel or Tissucol or Tricos or Vivostat or Voncento or Wilate or Wilnativ or Wilstart or Xyntha).tw,kw.

77. (Glubran or Gluetiss or Ifabond or Indermil or LiquiBand or TissuGlu).tw,kw.

78. Collagen Sponge/or Collagen Dressing/

79. Gelatin Sponge/or Gelfoam/

80. (Evithrom or Floseal or Hemopatch or Gel‐Flow or Gelfoam or Gelfilm or Recothrom or Surgifoam or Surgiflo* or "rh Thrombin" or Thrombi‐Gel or Thrombi‐Pad or Thrombin‐JMI or Thrombinar or Thrombogen or Thrombostat).tw,kw.

81. *Chitosan/

82. Hydrogel Dressing/

83. Fibrinogen plus Thrombin/

84. Polyvinyl Alcohol Sponge/

85. (porcine gelatin or bovine collagen or bovine gelatin or nu‐knit or arista or hemostase or vita sure or thrombin‐jmi or thrombinjmi or avicel or vivagel or lyostypt or tabotamp or arterx or omnex or veriset).tw,kw.

86. (polysaccharide adj (sphere* or hemostatic powder)).tw,kw.

87. ((polymer‐derived elastic* or polymer tissue adhesive* or elastic hydrogel* or glutaraldehyde or PEG‐based or polyurethane‐based tissue or polyethylene glycol* or polyvinyl alcohol‐based tissue or PVA‐based tissue or natural biopolymer* or polypeptide‐based or protein‐based or polysaccharide‐based or chitosan or poliglusam or cyanoacrylic or cyanoacrylate or cyacrin or dextran‐based or chondroitin sulfate‐based or mussel‐inspired elastic* or glycol hydrogel or polymer‐based) adj3 (glu* or seal* or adhesive* or topical* or local* or matrix or matrices or spong* or fleece* or foam* or scaffold* or patch* or sheet* or bandag* or aerosol* or dressing* or paste* or powder*)).tw,kw.

88. Oxidized Cellulose/

89. Oxidized Regenerated Cellulose/

90. Recombinant Thrombin/

91. Tachocomb/

92. (absorbable cellulose or resorbable cellulose or oxidi?ed cellulose or carboxycellulose or oxycellulose or cellulosic acid or oxycel or oxidi?ed regenerated cellulose).tw,kw.

93. (BioGlue or Progel or Duraseal or Coseal or FocalSeal or ADAL‐1 or AdvaSeal or Pleuraseal or Angio‐Seal or Avitene or Instat or Helitene or Helistat or TDM‐621 or Dermabond or Tissueseal or PolyStat or Raplixa or Spongostan or Surgicel).tw,kw.

94. (Tachosil or Traumstem or CoStasis or "GRF Glue" or GR‐Dial or Algosterile or TraumaStat or HemCon or ChitoFlex or Celox or QuikClot or WoundStat or Vitagel or TachSeal or TachoComb or Cryoseal).tw,kw.

95. (collagen‐thrombin or thrombin‐collagen or gelatin‐fibrinogen or fibrinogen‐gelatin or gelatin‐thrombin or thrombin‐gelatin or fibrinogen‐thrombin or thrombin‐fibrinogen or collagen‐fibrinogen or fibrinogen‐collagen or microfibrillar collagen).tw,kw.

96. or/69‐95

97. Bone Wax/

98. (bonewax* or bone wax* or bone putty or hemasorb or ostene).tw,kw.

99. or/97‐98

100. Prothrombin Complex/

101. (prothrombin adj5 (complex* or concentrate*)).tw,kw.

102. (PCC* or 3F‐PCC* or 4F‐PCC* or Beriplex* or Feiba* or Autoplex* or Ocplex* or Octaplex* or Kcentra* or Cofact or Prothrombinex* or "Proplex‐T" or Prothroraas* or Haemosolvex* or Prothromplex* or "HT Defix" or Facnyne* or Kaskadil* or Kedcom* or Confidex* or PPSB or Profil?ine* or Pronativ* or Proplex* or Prothar* or ProthoRAAS* or Protromplex* or "Pushu Laishi" or "Uman Complex").tw,kw.

103. or/100‐102

104. (((haemosta* or hemosta* or antihaemorrhag* or antihemorrhag* or anti haemorrhag* or anti‐hemorrhag*) adj5 (drug* or agent* or treat* or therap*)) or ((coagulat* or clotting) adj factor*)).tw,kw.

105. 50 or 54 or 59 or 62 or 65 or 68 or 96 or 99 or 103 or 104

106. Meta Analysis/

107. (meta analy* or metaanaly* or evidence‐based).ti.

108. ((meta analy* or metaanaly*) and (trials or studies)).ab.

109. Systematic Review/

110. ((systematic* or evidence‐based) adj2 (review* or overview*)).tw,kw.

111. (evidence synthes* or cochrane or medline or pubmed or embase or cinahl or cinhal or lilacs or "web of science" or science citation index or scopus or search terms or literature search or electronic search* or comprehensive search* or systematic search* or published articles or search strateg* or reference list* or bibliograph* or handsearch* or hand search* or manual* search*).ab.

112. ((electronic* or online) adj (sources or resources or databases)).ab.

113. ((additional adj (papers or articles or sources)) or (relevant adj (journals or articles))).ab.

114. or/106‐113

115. Review.pt.

116. (data extraction or selection criteria).ab.

117. 115 and 116

118. 114 or 117

119. Editorial.pt.

120. 118 not 119

121. crossover‐procedure/or double‐blind procedure/or randomized controlled trial/or single‐blind procedure/

122. (random* or factorial* or crossover* or cross over* or cross‐over* or placebo* or doubl* blind* or singl* blind* or assign* or allocat* or volunteer*).mp.

123. 120 or 121 or 122

124. (exp animal/or nonhuman/) not exp human/

125. 123 not 124

126. 43 and 105 and 125  

#### CINAHL (EBSCOhost)

S1 (MH "Femoral Fractures+") OR (MH "Ankle Fractures") OR (MH "Elbow Fractures") OR (MH "Fibula Fractures") OR (MH "Humeral Fractures+") OR (MH "Knee Fractures+") OR (MH "Osteoporotic Fractures") OR (MH "Pelvic Fractures") OR (MH "Periprosthetic Fractures") OR (MH "Radius Fractures") OR (MH "Shoulder Fractures+") OR (MH "Ulna Fractures+") OR (MH "Wrist Fractures+") OR (MH "Tibial Fractures+")

S2 TI ( ((pelvi* or sacrum or coccyx or ischium or pubis or pubic or ilium or tailbone or diaphys* or epiphys* or metaphys* or acetabulum or acetabular or femor* or femur* or hip* or thigh* or tibia* or fibula* or intertrochanteric or subtrochanteric or petrochanteric or intracapsular or subcapsular or subcapital or osteoporo* or osteoarthritis or orthopedic or orthopaedic) N6 (fracture* or break* or broke* or trauma* or injur* or surg* or operat* or repair* or reconstruct* or fixation* or implant* or prosthes* or "plate and screw" or "plate and screws" or intramedullary nail*)) ) OR AB ( ((pelvi* or sacrum or coccyx or ischium or pubis or pubic or ilium or tailbone or diaphys* or epiphys* or metaphys* or acetabulum or acetabular or femor* or femur* or hip* or thigh* or tibia* or fibula* or intertrochanteric or subtrochanteric or petrochanteric or intracapsular or subcapsular or subcapital or osteoporo* or osteoarthritis or orthopedic or orthopaedic) N6 (fracture* or break* or broke* or trauma* or injur* or surg* or operat* or repair* or reconstruct* or fixation* or implant* or prosthes* or "plate and screw" or "plate and screws" or intramedullary nail*)) )

S3 TI ( ((long bone* or long‐bone* or humerus or humeral or upper arm* or shoulder* or clavicle* or clavicula* or collar bone* or ankle* or pilon or lower leg* or calf* or knee* or tibiofibular or menisci or meniscus or femoropatellar or patellofemoral or radial or radius or ulna or forearm* or elbow*) N6 (fracture* or break* or broke* or trauma* or injur* or surg* or operat* or repair* or reconstruct* or implant* or fixation* or prosthes* or "plate and screw" or "plate and screws" or intramedullary nail*)) ) OR AB ( ((long bone* or long‐bone* or humerus or humeral or upper arm* or shoulder* or clavicle* or clavicula* or collar bone* or ankle* or pilon or lower leg* or calf* or knee* or tibiofibular or menisci or meniscus or femoropatellar or patellofemoral or radial or radius or ulna or wrist* or forearm* or elbow*) N6 (fracture* or break* or broke* or trauma* or injur* or surg* or operat* or repair* or reconstruct* or implant* or fixation* or prosthes* or "plate and screw" or "plate and screws" or intramedullary nail*)) )

S4 TI ( ((malleol* or talus or trochanteric or crural or crus or olecranon or antebrachial or monteggi* or bankart) N6 (fracture* or break* or broke* or trauma* or injur* or surg* or operat* or repair* or reconstruct* or fixation* or implant* or prosthes* or "plate and screw" or "plate and screws" or intramedullary nail*)) ) OR AB ( ((malleol* or talus or trochanteric or crural or crus or olecranon or antebrachial or monteggi* or bankart) N6 (fracture* or break* or broke* or trauma* or injur* or surg* or operat* or repair* or reconstruct* or fixation* or implant* or prosthes* or "plate and screw" or "plate and screws" or intramedullary nail*)) )

S5 TI ( ((wrist* or capitate or hamtae or lunate or carpal or metacarpal or pisiform or scaphoid or trapezium or triquetral) N6 (fracture* or break* or broke* or trauma* or injur* or surg* or operat* or repair* or reconstruct* or fixation* or implant* or prosthes* or "plate and screw" or "plate and screws" or intramedullary nail*)) ) OR AB ( ((wrist* or capitate or hamtae or lunate or carpal or metacarpal or pisiform or scaphoid or trapezium or triquetral) N6 (fracture* or break* or broke* or trauma* or injur* or surg* or operat* or repair* or reconstruct* or fixation* or implant* or prosthes* or "plate and screw" or "plate and screws" or intramedullary nail*)) )

S6 TI ( ((peri‐implant or periprosthetic) N1 fracture*) ) OR AB ( ((peri‐implant or periprosthetic) N1 fracture*) )

S7 (MH "Arm Bones+/IN/SU") OR (MH "Leg Bones+/IN/SU") OR (MH "Pelvic Bones+/IN/SU") OR (MH "Epiphyses/IN/SU") OR (MH "Diaphyses/IN/SU") OR (MH "Lower Extremity/IN/SU") OR (MH "Upper Extremity/IN/SU") S8 (MH "Ankle Joint/IN/SU") OR (MH "Elbow Joint/IN/SU") OR (MH "Hip Joint/IN/SU") OR (MH "Knee Joint+/IN/SU") OR (MH "Shoulder Joint+/IN/SU")

S9 (MH "Knee Injuries+/SU") OR (MH "Hip Injuries+/SU") OR (MH "Ankle Injuries+/SU")

S10 (MH "Hip/IN/SU") OR (MH "Knee/IN/SU") OR (MH "Leg/IN/SU") OR (MH "Thigh/IN/SU") OR (MH "Lower Extremity/IN/SU")

S11 (MH "Arm Injuries+/SU") OR (MH "Shoulder Injuries+/SU") S12 (MH "Arm/IN/SU") OR (MH "Elbow/IN/SU") OR (MH "Forearm/IN/SU") OR (MH "Shoulder/IN/SU")

S13 TI ( ((hip* or shoulder* or knee*) N5 (replac* or arthroplast* or hemiarthroplast* or hemi‐arthroplast*)) ) OR AB ( ((hip* or shoulder* or knee*) N5 (replac* or arthroplast* or hemiarthroplast* or hemi‐arthroplast*)) )

S14 (MH "Arm Bones+") OR (MH "Leg Bones+") OR (MH "Pelvic Bones+")

S15 (MH "Fractures+") OR (MH "Fracture Fixation+") OR TI (trauma* or fracture* or injur* or surg* or operat* or repair* or fixation)

S16 S14 AND S15

S17 S1 OR S2 OR S3 OR S4 OR S5 OR S6 OR S7 OR S8 OR S9 OR S10 OR S11 OR S12 OR S13 OR S16 S19 (MH "Antifibrinolytic Agents") OR (MH "Aminocaproic Acids") OR (MH "Tranexamic Acid")

S20 TI ( (antifibrinolytic* or anti‐fibrinolytic* or antifibrinolysin* or antiplasmin* or plasmin inhibitor* or tranexamic or tranhexamic or cyclohexanecarboxylic acid* or amcha or trans‐4‐aminomethyl‐cyclohexanecarboxylic acid* or t‐amcha or amca or "kabi 2161" or transamin or amchafibrin or anvitoff or spotof or cyklokapron or cyclo‐F or femstrual or ugurol or aminomethylcyclohexanecarbonic acid or aminomethylcyclohexanecarboxylic acid or AMCHA or amchafibrin or amikapron or aminomethyl cyclohexane carboxylic acid or aminomethyl cyclohexanecarboxylic acid or aminomethylcyclohexane carbonic acid or aminomethylcyclohexane carboxylic acid or aminomethylcyclohexanecarbonic acid or aminomethylcyclohexanecarboxylic acid or aminomethylcyclohexanocarboxylic acid or aminomethylcyclohexanoic acid or amstat or antivoff or caprilon or cl?65336 or cl65336 or cyclocapron or cyclokapron or cyklocapron or cyklokapron or exacyl or frenolyse or fibrinon or hemostan or hexacapron or hexakapron or kalnex or lysteda or rikaparin or ronex or theranex or tranexam or tranexanic or tranexic or trans achma or transexamic or trenaxin or TXA or (fibrinolysis N2 inhibitor*)) ) OR AB ( (antifibrinolytic* or anti‐fibrinolytic* or antifibrinolysin* or antiplasmin* or plasmin inhibitor* or tranexamic or tranhexamic or cyclohexanecarboxylic acid* or amcha or trans‐4‐aminomethyl‐cyclohexanecarboxylic acid* or t‐amcha or amca or "kabi 2161" or transamin or amchafibrin or anvitoff or spotof or cyklokapron or cyclo‐F or femstrual or ugurol or aminomethylcyclohexanecarbonic acid or aminomethylcyclohexanecarboxylic acid or AMCHA or amchafibrin or amikapron or aminomethyl cyclohexane carboxylic acid or aminomethyl cyclohexanecarboxylic acid or aminomethylcyclohexane carbonic acid or aminomethylcyclohexane carboxylic acid or aminomethylcyclohexanecarbonic acid or aminomethylcyclohexanecarboxylic acid or aminomethylcyclohexanocarboxylic acid or aminomethylcyclohexanoic acid or amstat or antivoff or caprilon or cl?65336 or cl65336 or cyclocapron or cyclokapron or cyklocapron or cyklokapron or exacyl or frenolyse or fibrinon or hemostan or hexacapron or hexakapron or kalnex or lysteda or rikaparin or ronex or theranex or tranexam or tranexanic or tranexic or trans achma or transexamic or trenaxin or TXA or (fibrinolysis N2 inhibitor*)) )

S21 TI ( (6‐aminohexanoic or amino?caproic or amino?hexanoic or amino caproic or amino‐caproic or amino‐n‐hexanoic or cy‐116 or cy116 or lederle or acikaprin or afibrin or amicar or caprocid or capracid or capramol or caprogel or caprolest or caprolisin* or caprolysin* or capromol or epsikapron or hemocaprol or caproamin or EACA or caprolest or capralense or hexalense or hamostat or hemocid or cl 10304 or cl10304 or ecapron or ekaprol or epsamon or epsicaprom or epsicapron or epsilcapramin or epsilon amino caproate or epsilon aminocaproate or epsilonaminocaproic or epsilonaminocapronsav or etha?aminocaproic or ethaaminocaproich or emocaprol or hepin or ipsilon or jd?177or neocaprol or nsc?26154 or resplamin or tachostyptan) ) OR AB ( (6‐aminohexanoic or amino?caproic or amino?hexanoic or amino caproic or amino‐caproic or amino‐n‐hexanoic or cy‐116 or cy116 or lederle or acikaprin or afibrin or amicar or caprocid or capracid or capramol or caprogel or caprolest or caprolisin* or caprolysin* or capromol or epsikapron or hemocaprol or caproamin or EACA or caprolest or capralense or hexalense or hamostat or hemocid or cl 10304 or cl10304 or ecapron or ekaprol or epsamon or epsicaprom or epsicapron or epsilcapramin or epsilon amino caproate or epsilon aminocaproate or epsilonaminocaproic or epsilonaminocapronsav or etha?aminocaproic or ethaaminocaproich or emocaprol or hepin or ipsilon or jd?177or neocaprol or nsc?26154 or resplamin or tachostyptan) )

S22 S19 OR S20 OR S21

S23 (MH "Aprotinin")

S24 TI ( (antagosan or antilysin* or aprotimbin or apronitin* or aprotinin* or bayer a128 or contrical or contrycal or contrykal or dilmintal or frey inhibitor or kontrycal or Kunitz inhibitor or gordox or haemoprot or kallikrein‐trypsin inactivator) ) OR AB ( (antagosan or antilysin* or aprotimbin or apronitin* or aprotinin* or bayer a128 or contrical or contrycal or contrykal or dilmintal or frey inhibitor or kontrycal or Kunitz inhibitor or gordox or haemoprot or kallikrein‐trypsin inactivator) )

S25 TI ( (iniprol or kontrikal or kontrykal or kunitz pancreatic trypsin inhibitor or midran or pulmin or tracylol or trascolan or trasilol or tra?ylol or traskolan or zymofren or pancreas antitrypsin or protinin or riker 52g or Rivilina zymofren) ) OR AB ( (iniprol or kontrikal or kontrykal or kunitz pancreatic trypsin inhibitor or midran or pulmin or tracylol or trascolan or trasilol or tra?ylol or traskolan or zymofren or pancreas antitrypsin or protinin or riker 52g or Rivilina zymofren) )

S26 S23 OR S24 OR S25

S27 TX ( (factor viia or factor 7a or rfviia or fviia or novoseven* or novo seven* or aryoseven or acset or eptacog* or proconvertin) ) OR TX ( ((activated N2 factor seven) or (activated N2 factor vii) or (activated N3 rfvii) or (activated N2 fvii)) )

S28 TX (factor seven or factor vii or factor 7)

S29 S27 OR S28

S30 (MH "Fibrinogen")

S31 TX (fibrinogen concentrate* or factor I or Haemocomplettan* or Riastap* or Fibryga* or Fibryna*)

S32 S30 OR S31

S33 (MH "Desmopressin")

S34 TI ( (desmopressin* or vasopressin deamino or D‐amino D‐arginine vasopressin or deamino‐8‐d‐arginine vasopressin or vasopressin 1‐desamino‐8‐arginine or desmotabs or DDAVP or desmogalen or adin or adiuretin or concentraid or d‐void or dav ritter or deamino 8 dextro arginine vasopressin or deamino 8d arginine vasopressin or deamino dextro arginine vasopressin or deaminovasopressin or defirin or defirin melt or desmirin or desmomelt or desmopresina or desmospray or desmotab* or desurin or emosint or enupresol or minirin or minirinette or minirinmelt or minrin or minurin or miram or nictur or noctisson or nocturin or nocutil or nordurine or novidin or nucotil or octim or octostim or presinex or stimate or wetirin) ) OR AB ( (desmopressin* or vasopressin deamino or D‐amino D‐arginine vasopressin or deamino‐8‐d‐arginine vasopressin or vasopressin 1‐desamino‐8‐arginine or desmotabs or DDAVP or desmogalen or adin or adiuretin or concentraid or d‐void or dav ritter or deamino 8 dextro arginine vasopressin or deamino 8d arginine vasopressin or deamino dextro arginine vasopressin or deaminovasopressin or defirin or defirin melt or desmirin or desmomelt or desmopresina or desmospray or desmotab* or desurin or emosint or enupresol or minirin or minirinette or minirinmelt or minrin or minurin or miram or nictur or noctisson or nocturin or nocutil or nordurine or novidin or nucotil or octim or octostim or presinex or stimate or wetirin) )

S35 S33 OR S34

S36 TX (factor XIII or fXIII or fibrin stabili?ing factor* or Tretten* or Catridecacog)

S37 (MH "Tissue Adhesives")

S38 (MH "Fibrin Tissue Adhesive")

S39 (MH "Collagen/TU")

S40 (MH "Thrombin/TU")

S41 (MH "Surgical Sponges")

S42 TI ( ((fibrin* or collagen or cellulose or gelatin or gel or thrombin* or albumin or hemostatic* or haemostatic*) N3 (glu* or seal* or adhesive* or topical* or local* or matrix or matrices or spong* or fleece* or foam* or scaffold* or patch* or sheet* or bandag* or aerosol* or dressing* or paste or powder*)) ) OR AB ( ((fibrin* or collagen or cellulose or gelatin or gel or thrombin* or albumin or hemostatic* or haemostatic*) N3 (glu* or seal* or adhesive* or topical* or local* or matrix or matrices or spong* or fleece* or foam* or scaffold* or patch* or sheet* or bandag* or aerosol* or dressing* or paste or powder*)) )

S43 TI ( ((nonfibrin* or non‐fibrin* or synthetic* or non‐biological* or nonbiological* or biological*) N3 (glue* or seal* or adhesive*)) ) OR AB ( ((nonfibrin* or non‐fibrin* or synthetic* or non‐biological* or nonbiological* or biological*) N3 (glue* or seal* or adhesive*)) )

S44 TI ( (surgical* N3 (glue* or sealant* or adhesive*)) ) OR AB ( (surgical* N3 (glue* or sealant* or adhesive*)) )

S45 TI ( ((fibrin* or collagen or cellulose or gelatin or thrombin) N3 (hemosta* or haemosta*)) ) OR AB ( ((fibrin* or collagen or cellulose or gelatin or thrombin) N3 (hemosta* or haemosta*)) )

S46 TI ( (8Y or Aafact or Actif‐VIII or Advate or Artiss or Bioglue or Biocol or Collaseal or Omrixil or Transglutine or Raplixa or Evarrest or Aleviate or Alphanate or Amofil or Beriate or Beriplast or Biostate or Bolheal or Cluvot or Conco‐Eight‐HT or Crosseel or Crosseal or Crosseight or Emoclot or Evarrest or Evicel or Factane or Fanhdi or Fibrogammin P or Green VIII or Green VIII Factor or Greengene or Greenmono or Greenplast or Haemate or Haemate P or Haemate P or Haemate P500 or Haemate‐P or Haemoctin or Haemoctin SDH or Haemoctin‐SDH or Hemaseel or Hemaseal or Hemofil M or Hemoraas or Humaclot or Humafactor‐8 or Humate‐P or Immunate or Innovate or Koate or Koate‐DVI or Kogenate Bayer or Kogenate FS or Monoclate‐P or NovoThirteen or Octafil or Octanate or Octanate or Optivate or Quixil or Talate or Tisseel or Tisseal or Tissel or Tissucol or Tricos or Vivostat or Voncento or Wilate or Wilnativ or Wilstart or Xyntha) ) OR AB ( (8Y or Aafact or Actif‐VIII or Advate or Artiss or Bioglue or Biocol or Collaseal or Omrixil or Transglutine or Raplixa or Evarrest or Aleviate or Alphanate or Amofil or Beriate or Beriplast or Biostate or Bolheal or Cluvot or Conco‐Eight‐HT or Crosseel or Crosseal or Crosseight or Emoclot or Evarrest or Evicel or Factane or Fanhdi or Fibrogammin P or Green VIII or Green VIII Factor or Greengene or Greenmono or Greenplast or Haemate or Haemate P or Haemate P or Haemate P500 or Haemate‐P or Haemoctin or Haemoctin SDH or Haemoctin‐SDH or Hemaseel or Hemaseal or Hemofil M or Hemoraas or Humaclot or Humafactor‐8 or Humate‐P or Immunate or Innovate or Koate or Koate‐DVI or Kogenate Bayer or Kogenate FS or Monoclate‐P or NovoThirteen or Octafil or Octanate or Octanate or Optivate or Quixil or Talate or Tisseel or Tisseal or Tissel or Tissucol or Tricos or Vivostat or Voncento or Wilate or Wilnativ or Wilstart or Xyntha) )

S47 TI ( (Glubran or Gluetiss or Ifabond or Indermil or LiquiBand or TissuGlu) ) OR AB ( (Glubran or Gluetiss or Ifabond or Indermil or LiquiBand or TissuGlu) )

S48 TI ( (Evithrom or Floseal or Hemopatch or Gel‐Flow or Gelfoam or Gelfilm or Recothrom or Surgifoam or Surgiflo* or "rh Thrombin" or Thrombi‐Gel or Thrombi‐Pad or Thrombin‐JMI or Thrombinar or Thrombogen or Thrombostat) ) OR AB ( (Evithrom or Floseal or Hemopatch or Gel‐Flow or Gelfoam or Gelfilm or Recothrom or Surgifoam or Surgiflo* or "rh Thrombin" or Thrombi‐Gel or Thrombi‐Pad or Thrombin‐JMI or Thrombinar or Thrombogen or Thrombostat) )

S49 TI ( (porcine gelatin or bovine collagen or bovine gelatin or nu‐knit or arista or hemostase or vita sure or thrombin‐jmi or thrombinjmi or avicel or vivagel or lyostypt or tabotamp or arterx or omnex or veriset) ) OR AB ( (porcine gelatin or bovine collagen or bovine gelatin or nu‐knit or arista or hemostase or vita sure or thrombin‐jmi or thrombinjmi or avicel or vivagel or lyostypt or tabotamp or arterx or omnex or veriset) )

S50 TX (polysaccharide NEXT (sphere* or hemostatic powder))

S51 (MM "Polyethylene Glycols")

S52 (MH "Hydrogel Dressings")

S53 (MH "Polyurethanes/AD/AE/TU/ST/DE")

S54 TI ( ((polymer‐derived elastic* or polymer tissue adhesive* or elastic hydrogel* or glutaraldehyde or PEG‐based or polyurethane‐based tissue or polyethylene glycol* or polyvinyl alcohol‐based tissue or PVA‐based tissue or natural biopolymer* or polypeptide‐based or protein‐based or polysaccharide‐based or chitosan or poliglusam or cyanoacrylic or cyanoacrylate or cyacrin or dextran‐based or chondroitin sulfate‐based or mussel‐inspired elastic* or glycol hydrogel or polymer‐based) N3 (glu* or seal* or adhesive* or topical* or local* or matrix or matrices or spong* or fleece* or foam* or scaffold* or patch* or sheet* or bandag* or aerosol* or dressing* or paste* or powder*)) ) OR AB ( ((polymer‐derived elastic* or polymer tissue adhesive* or elastic hydrogel* or glutaraldehyde or PEG‐based or polyurethane‐based tissue or polyethylene glycol* or polyvinyl alcohol‐based tissue or PVA‐based tissue or natural biopolymer* or polypeptide‐based or protein‐based or polysaccharide‐based or chitosan or poliglusam or cyanoacrylic or cyanoacrylate or cyacrin or dextran‐based or chondroitin sulfate‐based or mussel‐inspired elastic* or glycol hydrogel or polymer‐based) N3 (glu* or seal* or adhesive* or topical* or local* or matrix or matrices or spong* or fleece* or foam* or scaffold* or patch* or sheet* or bandag* or aerosol* or dressing* or paste* or powder*)) )

S55 (MH "Cellulose/TU")

S56 TI ( (absorbable cellulose or resorbable cellulose or oxidi?ed cellulose or carboxycellulose or oxycellulose or cellulosic acid or oxycel or oxidi?ed regenerated cellulose) ) OR AB ( (absorbable cellulose or resorbable cellulose or oxidi?ed cellulose or carboxycellulose or oxycellulose or cellulosic acid or oxycel or oxidi?ed regenerated cellulose) )

S57 TI ( (BioGlue or Progel or Duraseal or Coseal or FocalSeal or ADAL‐1 or AdvaSeal or Pleuraseal or Angio‐Seal or Avitene or Instat or Helitene or Helistat or TDM‐621 or Dermabond or Tissueseal or PolyStat or Raplixa or Spongostan or Surgicel or Surgilux or Tachosil or Traumstem) ) OR AB ( (BioGlue or Progel or Duraseal or Coseal or FocalSeal or ADAL‐1 or AdvaSeal or Pleuraseal or Angio‐Seal or Avitene or Instat or Helitene or Helistat or TDM‐621 or Dermabond or Tissueseal or PolyStat or Raplixa or Spongostan or Surgicel or Surgilux or Tachosil or Traumstem) )

S58 TI ( (collagen‐thrombin or thrombin‐collagen or gelatin‐fibrinogen or fibrinogen‐gelatin or gelatin‐thrombin or thrombin‐gelatin or fibrinogen‐thrombin or thrombin‐fibrinogen or collagen‐fibrinogen or fibrinogen‐collagen or microfibrillar collagen or CoStasis or "GRF Glue" or GR‐Dial or Algosterile or TraumaStat or HemCon or ChitoFlex or Celox or QuikClot or WoundStat or Vitagel or TachSeal or TachoComb or Cryoseal) ) OR AB ( (collagen‐thrombin or thrombin‐collagen or gelatin‐fibrinogen or fibrinogen‐gelatin or gelatin‐thrombin or thrombin‐gelatin or fibrinogen‐thrombin or thrombin‐fibrinogen or collagen‐fibrinogen or fibrinogen‐collagen or microfibrillar collagen or CoStasis or "GRF Glue" or GR‐Dial or Algosterile or TraumaStat or HemCon or ChitoFlex or Celox or QuikClot or WoundStat or Vitagel or TachSeal or TachoComb or Cryoseal) )

S59 S36 OR S37 OR S38 OR S39 OR S40 OR S41 OR S42 OR S43 OR S44 OR S45 OR S46 OR S47 OR S48 OR S49 OR S50 OR S51 OR S52 OR S53 OR S54 OR S55 OR S56 OR S57 OR S58

S60 (MH "Waxes/TU")

S61 TI ( (bonewax* or bone wax* or bone putty or hemasorb or ostene) ) OR AB ( (bonewax* or bone wax* or bone putty or hemasorb or ostene) )

S62 S60 OR S61

S63 TI ( (((haemosta* or hemosta* or antihaemorrhag* or antihemorrhag* or anti haemorrhag* or anti‐hemorrhag*) N5 (drug* or agent* or treat* or therap*)) or ((coagulat* or clotting) NEXT factor*)) ) OR AB ( (((haemosta* or hemosta* or antihaemorrhag* or antihemorrhag* or anti haemorrhag* or anti‐hemorrhag*) N5 (drug* or agent* or treat* or therap*)) or ((coagulat* or clotting) NEXT factor*)) )

S64 S22 OR S26 OR S29 OR S32 OR S35 OR S59 OR S62 OR S63

S65 (MH Clinical Trials+)

S66 PT Clinical Trial

S67 TI ((controlled trial*) or (clinical trial*)) OR AB ((controlled trial*) or (clinical trial*))

S68 TI ((singl* blind*) OR (doubl* blind*) OR (trebl* blind*) OR (tripl* blind*) OR (singl* mask*) OR (doubl* mask*) OR (tripl* mask*)) OR AB ((singl* blind*) OR (doubl* blind*) OR (trebl* blind*) OR (tripl* blind*) OR (singl* mask*) OR (doubl* mask*) OR (tripl* mask*))

S69 TI randomi* OR AB randomi*

S70 MH RANDOM ASSIGNMENT

S71 TI ((phase three) or (phase III) or (phase three)) or AB ((phase three) or (phase III) or (phase three))

S72 ( TI (random* N2 (assign* or allocat*)) ) OR ( AB (random* N2 (assign* or allocat*)) )

S73 MH PLACEBOS

S74 MH META ANALYSIS

S75 MH SYSTEMATIC REVIEW

S76 TI ("meta analys*" OR metaanalys* OR "systematic review" OR "systematic overview" OR "systematic search*") OR AB ("meta analys*" OR metaanalys* OR "systematic review" OR "systematic overview" OR "systematic search*")

S77 TI ("literature review" OR "literature overview" OR "literature search*") OR AB ("literature review" OR "literature overview" OR "literature search*")

S78 TI (cochrane OR embase OR cinahl OR cinhal OR lilacs OR BIDS OR science AND citation AND index OR cancerlit) OR AB (cochrane OR embase OR cinahl OR cinhal OR lilacs OR BIDS OR science AND citation AND index OR cancerlit)

S79 TI placebo* OR AB placebo*

S80 MH QUANTITATIVE STUDIES

S81 S65 or S66 or S67 or S68 or S69 or S70 or S71 or S72 or S73 or S74 or S75 or S76 or S77 or S78 or S79 or S80

S82 (MH "Blood Coagulation Factors") OR (MH "Prothrombin")

S83 TI ( (prothrombin N5 (complex* or concentrate*)) ) OR AB ( (prothrombin N5 (complex* or concentrate*)) )

S84 TI ( (PCC* or 3F‐PCC* or 4F‐PCC* or Beriplex* or Feiba* or Autoplex* or Ocplex* or Octaplex* or Kcentra* or Cofact or Prothrombinex* or "Proplex‐T" or Prothroraas* or Haemosolvex* or Prothromplex* or "HT Defix" or Facnyne* or Kaskadil* or Kedcom* or Confidex* or PPSB or Profil?ine* or Pronativ* or Proplex* or Prothar* or ProthoRAAS* or Protromplex* or "Pushu Laishi" or "Uman Complex") ) OR AB ( (PCC* or 3F‐PCC* or 4F‐PCC* or Beriplex* or Feiba* or Autoplex* or Ocplex* or Octaplex* or Kcentra* or Cofact or Prothrombinex* or "Proplex‐T" or Prothroraas* or Haemosolvex* or Prothromplex* or "HT Defix" or Facnyne* or Kaskadil* or Kedcom* or Confidex* or PPSB or Profil?ine* or Pronativ* or Proplex* or Prothar* or ProthoRAAS* or Protromplex* or "Pushu Laishi" or "Uman Complex") )

S85 S82 OR S83 OR S84

S86 S64 OR S85

S87 S18 AND S81 AND S86

#### Transfusion Evidence Library

Clinical Specialty: Orthopaedic Surgery AND Subject Areas: Alternatives to Blood/Antifibrinolytics OR Alternatives to Blood/Fractionated Blood Products OR Alternatives to Blood/Recombinant Coagulation Factors  

#### ClinicalTrials.gov

1. Other terms: randomized or randomised OR randomly OR random Condition: fracture OR long bone OR pelvic OR ischium OR pubis OR pubic OR ilium OR acetabular OR acetabulum OR diaphysis OR epiphysis OR metaphysis OR femoral OR femur OR hip OR knee OR shoulder OR clavicle OR humerus OR humeral OR tibia OR tibial OR fibula OR ankle OR pilon OR ulna OR radius OR radial OR elbow OR intertrochanteric OR subtrochanteric OR petrochanteric OR intracapsular OR subcapsular OR osteoporosis OR osteoporotic OR osteoarthritis OR orthopedic trauma OR surgical fixation OR hemiarthroplasty OR arthroplasty OR periprosthetic Intervention: hemostatic OR antifibrinolytic OR tranexamic OR EACA OR aminocaproic OR aprotinin OR desmopressin OR DDAVP OR factor viia OR novoseven OR aryoseven OR fibrinogen OR haemocomplettan OR Riastap OR Fibryna OR Fibryga OR factor XIII OR Tretten Study Type: Interventional Studies (Clinical Trials)

2. Other terms: randomized or randomised OR randomly OR random Condition: fracture OR long bone OR pelvic OR ischium OR pubis OR pubic OR ilium OR acetabular OR acetabulum OR diaphysis OR epiphysis OR metaphysis OR femoral OR femur OR hip OR knee OR shoulder OR clavicle OR humerus OR humeral OR tibia OR tibial OR fibula OR ankle OR pilon OR ulna OR radius OR radial OR elbow OR intertrochanteric OR subtrochanteric OR petrochanteric OR intracapsular OR subcapsular OR osteoporosis OR osteoporotic OR osteoarthritis OR orthopedic trauma OR surgical fixation OR hemiarthroplasty OR arthroplasty OR periprosthetic Intervention: sealant OR adhesive OR collagen OR cellulose OR gelatin OR glue OR matrix OR sponge OR fleece OR foam OR scaffold OR patch OR sheet OR gelfoam OR chitosan OR hydrogel OR polyethylene glycol OR tachocomb OR BioGlue OR Surgicel OR Veriset Study Type: Interventional Studies (Clinical Trials) 3. Other terms: randomized or randomised OR randomly OR random Condition: fracture OR long bone OR pelvic OR ischium OR pubis OR pubic OR ilium OR acetabular OR acetabulum OR diaphysis OR epiphysis OR metaphysis OR femoral OR femur OR hip OR knee OR shoulder OR clavicle OR humerus OR humeral OR tibia OR tibial OR fibula OR ankle OR pilon OR ulna OR radius OR radial OR elbow OR intertrochanteric OR subtrochanteric OR petrochanteric OR intracapsular OR subcapsular OR osteoporosis OR osteoporotic OR osteoarthritis OR orthopedic trauma OR surgical fixation OR hemiarthroplasty OR arthroplasty OR periprosthetic Intervention: Evithrom OR Floseal OR Tachosil OR Cryoseal OR Hemopatch OR Progel OR Duraseal OR Coseal OR FocalSeal OR Algosterile OR TraumaStat OR HemCon OR ChitoFlex OR Celox OR QuikClot OR WoundStat OR Vitagel OR TachSeal OR bonewax OR hemasorb OR ostene Study Type: Interventional Studies (Clinical Trials)  

4. Other terms: randomized or randomised OR randomly OR random Condition: fracture OR long bone OR pelvic OR ischium OR pubis OR pubic OR ilium OR acetabular OR acetabulum OR diaphysis OR epiphysis OR metaphysis OR femoral OR femur OR hip OR knee OR shoulder OR clavicle OR humerus OR humeral OR tibia OR tibial OR fibula OR ankle OR pilon OR ulna OR radius OR radial OR elbow OR intertrochanteric OR subtrochanteric OR petrochanteric OR intracapsular OR subcapsular OR osteoporosis OR osteoporotic OR osteoarthritis OR orthopedic trauma OR surgical fixation OR hemiarthroplasty OR arthroplasty OR periprosthetic Intervention: iniprol or kontrikal OR CloSys OR Glubran OR Gluetiss OR Ifabond OR Indermil OR LiquiBand OR Octafil OR Octanate OR Optivate OR Quixil OR Tisseel OR Tissucol OR TissuGlu OR Thrombi‐Gel OR Vivostat OR Voncento OR Wilate OR Wilnativ OR Wilstart Study Type: Interventional Studies (Clinical Trials)  

5. Other terms: randomized or randomised OR randomly OR random Condition: fracture OR long bone OR pelvic OR ischium OR pubis OR pubic OR ilium OR acetabular OR acetabulum OR diaphysis OR epiphysis OR metaphysis OR femoral OR femur OR hip OR knee OR shoulder OR clavicle OR humerus OR humeral OR tibia OR tibial OR fibula OR ankle OR pilon OR ulna OR radius OR radial OR elbow OR intertrochanteric OR subtrochanteric OR petrochanteric OR intracapsular OR subcapsular OR osteoporosis OR osteoporotic OR osteoarthritis OR orthopedic trauma OR surgical fixation OR hemiarthroplasty OR arthroplasty OR periprosthetic Title: hemostasis OR hemostatic OR antifibrinolytic OR factor OR fibrinogen OR thrombin OR collagen OR gelatin OR cellulose Study Type: Interventional Studies (Clinical Trials)  

6. Other Terms: fracture OR long bone OR pelvic OR ischium OR pubis OR pubic OR ilium OR acetabular OR acetabulum OR diaphysis OR epiphysis OR metaphysis OR femoral OR femur OR hip OR knee OR shoulder OR clavicle OR humerus OR humeral OR tibia OR tibial OR fibula OR ankle OR pilon OR ulna OR radius OR radial OR elbow OR intertrochanteric OR subtrochanteric OR petrochanteric OR intracapsular OR subcapsular OR osteoporosis OR osteoporotic OR osteoarthritis OR orthopedic trauma OR surgical fixation OR hemiarthroplasty OR arthroplasty OR periprosthetic Study Type: Interventional Studies (Clinical Trials) Condition: bleeding OR haemorrhage OR hemorrhage OR blood loss OR bloodloss  

7. 1 OR 2 OR 3 OR 4 OR 5 OR 6 [N.B. combined and de‐duplicated in EndNote]

#### World Health Organization International Clinical Trials Registry Platform (ICTRP)

1. Title OR Condition: fracture OR long bone OR pelvic OR ischium OR pubis OR pubic OR ilium OR acetabular OR acetabulum OR femoral OR femur OR hip OR knee OR shoulder OR clavicle OR collar bone OR diaphysis OR epiphysis OR metaphysis OR humerus OR humeral OR tibia OR tibial OR fibula OR ankle OR pilon OR ulna OR radius OR radial OR elbow OR intertrochanteric OR subtrochanteric OR petrochanteric OR intracapsular OR subcapsular OR osteoporosis OR osteoporotic OR osteoarthritis OR orthopedic trauma OR surgical fixation OR hemiarthroplasty OR arthroplasty OR periprosthetic Intervention: hemostatic OR antifibrinolytic OR tranexamic OR EACA OR aminocaproic OR aprotinin OR desmopressin OR DDAVP OR factor viia OR novoseven OR aryoseven OR fibrinogen OR haemocomplettan OR Riastap OR Fibryna OR Fibryga OR factor XIII OR Tretten Recruitment Status: ALL  

2. Title OR Condition: fracture OR long bone OR pelvic OR ischium OR pubis OR pubic OR ilium OR acetabular OR acetabulum OR femoral OR femur OR hip OR knee OR shoulder OR clavicle OR collar bone OR diaphysis OR epiphysis OR metaphysis OR humerus OR humeral OR tibia OR tibial OR fibula OR ankle OR pilon OR ulna OR radius OR radial OR elbow OR intertrochanteric OR subtrochanteric OR petrochanteric OR intracapsular OR subcapsular OR osteoporosis OR osteoporotic OR osteoarthritis OR orthopedic trauma OR surgical fixation OR hemiarthroplasty OR arthroplasty OR periprosthetic Intervention: sealant OR adhesive OR collagen OR cellulose OR gelatin OR glue OR matrix OR sponge OR fleece OR foam OR scaffold OR patch OR sheet OR gelfoam OR chitosan OR hydrogel OR polyethylene glycol OR tachocomb OR BioGlue OR Surgicel OR Veriset Recruitment Status: ALL  

3. Title OR Condition: fracture OR long bone OR pelvic OR ischium OR pubis OR pubic OR ilium OR acetabular OR acetabulum OR femoral OR femur OR hip OR knee OR shoulder OR clavicle OR collar bone OR diaphysis OR epiphysis OR metaphysis OR humerus OR humeral OR tibia OR tibial OR fibula OR ankle OR pilon OR ulna OR radius OR radial OR elbow OR intertrochanteric OR subtrochanteric OR petrochanteric OR intracapsular OR subcapsular OR osteoporosis OR osteoporotic OR osteoarthritis OR orthopedic trauma OR surgical fixation OR hemiarthroplasty OR arthroplasty OR periprostheticIntervention: Evithrom OR Floseal OR Tachosil OR Cryoseal OR Hemopatch OR Progel OR Duraseal OR Coseal OR FocalSeal OR Algosterile OR TraumaStat OR HemCon OR ChitoFlex OR Celox OR QuikClot OR WoundStat OR Vitagel OR TachSeal OR bonewax OR hemasorb OR ostene Recruitment Status: ALL

4. Title OR Condition: fracture OR long bone OR pelvic OR ischium OR pubis OR pubic OR ilium OR acetabular OR acetabulum OR femoral OR femur OR hip OR knee OR shoulder OR clavicle OR collar bone OR diaphysis OR epiphysis OR metaphysis OR humerus OR humeral OR tibia OR tibial OR fibula OR ankle OR pilon OR ulna OR radius OR radial OR elbow OR intertrochanteric OR subtrochanteric OR petrochanteric OR intracapsular OR subcapsular OR osteoporosis OR osteoporotic OR osteoarthritis OR orthopedic trauma OR surgical fixation OR hemiarthroplasty OR arthroplasty OR periprosthetic Intervention: iniprol or kontrikal OR CloSys OR Glubran OR Gluetiss OR Ifabond OR Indermil OR LiquiBand OR Octafil OR Octanate OR Optivate OR Quixil OR Tisseel OR Tissucol OR TissuGlu OR Thrombi‐Gel OR Vivostat OR Voncento OR Wilate OR Wilnativ OR Wilstart Recruitment Status: ALL

5. Title: fracture OR long bone OR pelvic OR ischium OR pubis OR pubic OR ilium OR acetabular OR acetabulum OR femoral OR femur OR hip OR knee OR shoulder OR humerus OR humeral OR tibia OR tibial OR fibula OR ankle OR pilon OR ulna OR radius OR radial OR elbow OR intertrochanteric OR subtrochanteric OR petrochanteric OR intracapsular OR subcapsular OR osteoporosis OR osteoporotic OR osteoarthritis OR orthopedic trauma OR surgical fixation OR hemiarthroplasty OR arthroplasty OR periprosthetic Condition: bleeding OR hemorrhage OR haemorrhage OR blood loss OR bloodloss Recruitment Status: ALL

6. 1 OR 2 OR 3 OR 4 OR 5 [N.B. combined and de‐duplicated in EndNote]

### Appendix 3. Details regarding contact with study authors

We contacted 12 authors for information on their study to determine eligibility or missing or unclear data in the published literature. 

**Table:**

**Study**	**Date of 1^st^ email sent**	**Information requested by review authors**	**Information provided by study authors**
ACTRN 12617000391370	26 May 2022	Update on status of trial	Update on trials status given
Baruah 2016	23 June 2021	Trial registration number	Confirmed not registered (only ethics registered with the Hospital)
Batibay 2018	23 June 2021	Trial registration number	Confirmed not registered (only ethics registered with the Hospital)
ChiCTR 1800016634	19 May 2021	Update on status of trial	Trial was stopped, and did not enrol any patients
CTRI/2019/04/018735	24 May 2021	Update on status of trial	Stated he would be in touch with further update
	24 May 2021	Update on status of trial	Stated that a publication was being prepared
CTRI/2019/09/021302	13 Oct 2021	Update on status of trial	Update on trials status given, and some slides of the results from her dissertation
CTRI/2019/10/021667	13 Oct 2021	Update on status of trial	Update on trials status given
CTRI/2021/09/036855	25 May 2022	Update on status of trial, and detailed breakdown of population types	Update on trials status given, as well as more clarification on population types (% of split between trauma and elective also given)
Sahni 2021	25 May 2022	Trial registration number	Thanked for email but no trial registration given
Shodipo 2022	24 May 2022	Trial registration number	Trial was not registered

## Data and analyses

**Comparison 1:** TXA (IV) vs placebo

Outcome or subgroup title	No. of studies	No. of participants	Statistical method	Effect size
1.1 Risk of requiring allogeneic blood transfusion (30 days)	6	457	Risk Ratio (M‐H, Random, 95% CI)	0.48 [0.34, 0.69]
1.1.1 Hip fixation	4	330	Risk Ratio (M‐H, Random, 95% CI)	0.47 [0.34, 0.64]
1.1.2 Mixed	2	127	Risk Ratio (M‐H, Random, 95% CI)	0.17 [0.00, 8.33]
1.2 All‐cause mortality (30 days)	2	147	Peto Odds Ratio (Peto, Fixed, 95% CI)	0.38 [0.05, 2.77]
1.2.1 Hip fixation	1	80	Peto Odds Ratio (Peto, Fixed, 95% CI)	0.53 [0.05, 5.26]
1.2.2 Mixed	1	67	Peto Odds Ratio (Peto, Fixed, 95% CI)	0.15 [0.00, 7.46]
1.3 Risk of MI (30 days)	2		Risk Difference (M‐H, Random, 95% CI)	Subtotals only
1.3.1 Hip fixation	2	199	Risk Difference (M‐H, Random, 95% CI)	0.00 [‐0.03, 0.03]
1.4 Risk of CVA/stroke (30 days)	3	324	Risk Difference (M‐H, Random, 95% CI)	0.00 [‐0.02, 0.02]
1.4.1 Hip fixation	3	324	Risk Difference (M‐H, Random, 95% CI)	0.00 [‐0.02, 0.02]
1.5 Risk of DVT (30 days)	4	329	Peto Odds Ratio (Peto, Fixed, 95% CI)	2.15 [0.22, 21.35]
1.5.1 Hip fixation	2	202	Peto Odds Ratio (Peto, Fixed, 95% CI)	2.15 [0.22, 21.35]
1.5.2 Mixed	2	0	Peto Odds Ratio (Peto, Fixed, 95% CI)	Not estimable
1.6 Risk of PE (30 days)	4	329	Peto Odds Ratio (Peto, Fixed, 95% CI)	1.08 [0.07, 17.66]
1.6.1 Hip fixation	2	202	Peto Odds Ratio (Peto, Fixed, 95% CI)	1.08 [0.07, 17.66]
1.6.2 Mixed	2	0	Peto Odds Ratio (Peto, Fixed, 95% CI)	Not estimable
1.7 Risk of suspected serious drug reactions (30 days)	2	185	Risk Difference (M‐H, Random, 95% CI)	0.00 [‐0.03, 0.03]
1.7.1 Hip fixation	1	125	Risk Difference (M‐H, Random, 95% CI)	0.00 [‐0.03, 0.03]
1.7.2 Mixed	1	60	Risk Difference (M‐H, Random, 95% CI)	0.00 [‐0.06, 0.06]

**Comparison 2:** TXA (topical) vs placebo

Outcome or subgroup title	No. of studies	No. of participants	Statistical method	Effect size
2.1 Risk of requiring allogeneic blood transfusion (30 days)	2	101	Risk Ratio (M‐H, Random, 95% CI)	0.31 [0.08, 1.22]
2.1.1 Hip arthroplasty	1	36	Risk Ratio (M‐H, Random, 95% CI)	0.10 [0.01, 1.70]
2.1.2 Mixed	1	65	Risk Ratio (M‐H, Random, 95% CI)	0.44 [0.09, 2.10]
2.2 All‐cause mortality (30 days)	1		Risk Difference (M‐H, Random, 95% CI)	Totals not selected
2.2.1 Hip arthroplasty	1		Risk Difference (M‐H, Random, 95% CI)	Totals not selected
2.3 Risk of MI (30 days)	1		Peto Odds Ratio (Peto, Fixed, 95% CI)	Totals not selected
2.3.1 Hip arthroplasty	1		Peto Odds Ratio (Peto, Fixed, 95% CI)	Totals not selected
2.4 Risk of CVA/stroke (30 days)	1		Risk Difference (M‐H, Random, 95% CI)	Totals not selected
2.4.1 Mixed	1		Risk Difference (M‐H, Random, 95% CI)	Totals not selected
2.5 Risk of DVT (30 days)	2	101	Peto Odds Ratio (Peto, Fixed, 95% CI)	1.11 [0.07, 17.77]
2.5.1 Hip arthroplasty	1	36	Peto Odds Ratio (Peto, Fixed, 95% CI)	8.31 [0.16, 421.42]
2.5.2 Mixed	1	65	Peto Odds Ratio (Peto, Fixed, 95% CI)	0.15 [0.00, 7.48]

**Comparison 3:** rFVIIa vs placebo

Outcome or subgroup title	No. of studies	No. of participants	Statistical method	Effect size
3.1 Risk of requiring allogeneic blood transfusion (30 days)	1		Risk Ratio (M‐H, Random, 95% CI)	Totals not selected
3.2 Re‐operation due to bleeding (7 days)	1		Peto Odds Ratio (Peto, Fixed, 95% CI)	Totals not selected
3.3 Risk of DVT (30 days)	1		Risk Difference (M‐H, Random, 95% CI)	Subtotals only
3.4 Risk of PE (30 days)	1		Risk Difference (M‐H, Random, 95% CI)	Subtotals only
3.5 Risk of suspected serious drug reaction (30 days)	1		Risk Difference (M‐H, Random, 95% CI)	Subtotals only

## Characteristics of studies

### Characteristics of included studies [ordered by study ID]

**Table:** Costain 2021

***Study characteristics***		
Methods	**Study design:** RCT (parallel) **Length of duration of study:** 16 months (November 2017‐February 2019) **Power calculation reached:** for blood loss; not for other outcomes **Transfusion strategy:** yes **Was the trial stopped early:** no **Follow up:** 30 days except mortality (90 days)	
Participants	**Baseline characteristics** **Placebo arm** Age (years) (mean SD): 79.03 (10.42) Gender (male, female): 9 M (26.4%), 25 F (73.5%) Length of surgery (surgical time) (min) (mean SD): not reported Proportion of participants on anticoagulants and/ or antiplatelets prior to surgery (n/N, %): not reported Incidence of preoperative anaemia: not reported Co‐morbidities: not reported ASA I (n/N,%): 0/34, 0% ASA II (n/N,%): 2/34, 5.9% ASA III (n/N,%): 18/34, 52.9% ASA IV (n/N,%): 14/34, 41.2% Number of participants randomised: unclear Number of participants receiving treatment: 34 Number of participants analysed: 34 Dropout rate: 0/34, 0% **TXA (topical) arm** Age (years) (mean SD): 80.32 (10.73) Gender (male, female): 11 M (35.4%), 20 F (64.5%), Length of surgery (surgical time) (min) (mean SD): not reported Proportion of participants on anticoagulants and/or antiplatelets prior to surgery (n/N, %): not reported Incidence of preoperative anaemia: not reported Co‐morbidities: not reported ASA I (n/N,%): 0/31, 0% ASA II (n/N,%): 2/31, 6.4% ASA III (n/N,%): 20/31, 64.5% ASA IV (n/N,%): 9/31, 29.0% Number of participants randomised: unclear Number of participants receiving treatment: 31 Number of participants analysed: 31 Dropout rate: 0/31, 0% **Inclusion criteria** ≥ 18 years of age Diagnosis of hip fracture (intracapsular, intratrochanteric or subtrochanteric) requiring surgical repair Patient/surrogate decision maker provide signed informed consent **Exclusion criteria** Not reported **Tourniquet use:** not reported **Type of anaesthetic:** not reported **Type of surgery:** not reported	
Interventions	**Placebo arm** 50 mL saline control, applied topically at the time of surgery. With open procedures (i.e. hemi‐arthroplasty), the solution was applied directly to the surgical wound and evacuated by suction after 3 min with no further wound irrigation **TXA (topical) arm** 3 g TXA in 50 mL saline applied topically at the time of surgery. With open procedures (i.e. hemi‐arthroplasty), the solution was applied directly to the surgical wound and evacuated by suction after 3 min with no further wound irrigation	
Outcomes	**Primary outcomes** Change in Hb levels Number receiving allogeneic blood transfusion **Secondary outcomes** All‐cause mortality Incidence of VTE Peri‐operative complication rate	
Notes	**Sponsorship source:** non‐pharma (this study was supported by grants from the Northern Ontario Academic Medicine Association and the Sault Ste. Marie Academic Medical Association) **Country:** Canada **Setting:** single‐centre, community hospital **Comments:** none **Authors name:** D Costain **Institution:** Northern Ontario School of Medicine, Sault Ste. Marie Ontario **Email:** dcostain@gmail.com **Address:** Northern Ontario School of Medicine, Division of Orthopedic Surgery, Sault Area Hospital, 955 Queen St E, Suite 100 Sault Ste. Marie ON P6A 2C3 **Native language of paper:** English and French **Reference type:** full text (1), trial registration (1) **Trial registration number:** NCT02993341 (clinicaltrials.gov) **Was it translated for this review:** no	
***Risk of bias***		
**Bias**	**Authors' judgement**	**Support for judgement**
Random sequence generation (selection bias)	Low risk	Quote: participants were randomly allocated to a treatment group using Graphpad Prism, which creates an equally divided treatment algorithm generated using the time of day to create the first random number. Judgement comment: an adequate method was used to generate the random sequence.
Allocation concealment (selection bias)	Low risk	Quote: participants were randomly allocated to a treatment group using Graphpad Prism, which creates an equally divided treatment algorithm generated using the time of day to create the first random number. Judgement comment: the pharmacy technician was privy to the treatment allocation, and treatment group allocation was maintained in a secure binder in the pharmacy.
Blinding of participants and personnel (performance bias) subjective outcomes	Low risk	Quote: none Judgement comment: the participant, surgeon, statistician and clinical staff entering data were not aware of treatment group allocation until all data were tabulated. The medication was delivered to the operating theatre with the participant’s name, participant number and date, without identifying the medication or placebo.
Blinding of participants and personnel (performance bias) objective outcomes	Low risk	Quote: none Judgement comment: the participant, surgeon, statistician and clinical staff entering data were not aware of treatment group allocation until all data were tabulated. The medication was delivered to the operating theatre with the participant’s name, participant number and date, without identifying the medication or placebo.
Blinding of outcome assessment (detection bias) subjective outcomes	Low risk	Quote: none Judgement comment: the participant, surgeon, statistician and clinical staff entering data were not aware of treatment group allocation until all data were tabulated. The medication was delivered to the operating theatre with the participant’s name, participant number and date, without identifying the medication or placebo.
Blinding of outcome assessment (detection bias) objective outcomes	Low risk	Quote: none Judgement comment: the participant, surgeon, statistician and clinical staff entering data were not aware of treatment group allocation until all data were tabulated. The medication was delivered to the operating theatre with the participant’s name, participant number and date, without identifying the medication or placebo.
Incomplete outcome data (attrition bias) All outcomes	Low risk	Quote: none Judgement comment: It is not clear when randomisation occurred in the timeline of the trial. The trial reports what happened to all patients approached. Outcome data provided for the participants detailed as being analysed per treatment group. 9 exclusions explained. Analysis as ITT based on those randomised
Selective reporting (reporting bias)	Low risk	Quote: none Judgement comment: trial registration checked. Data was reported for all outcomes detailed in the trial registration. Primary outcome (Hb and transfusions) were reported. Mortality was meant to be reported at 30 days, but was only reported at 90 days. PE and MI was not reported.
Other bias	Unclear risk	Quote: none Judgement comment: baseline imbalance in some domains ‐ renal function and smoker status; unclear whether this could impact outcomes

**Table:** Haghighi 2017

***Study characteristics***		
Methods	**Study design:** RCT **Length of duration of study:** not reported **Power calculation reached:** no: target sample size n = 80 (calculation not reported), enrolled n = 40 **Transfusion strategy:** not reported **Was the trial stopped early:** not reported **Follow up:** 24 hours	
Participants	**Baseline characteristics** **Placebo arm** Age (years) (mean SD): 66.15 (8.51) Gender (male, female): 17 M (85%), 3 F (15%) Length of surgery (min): 115.00 (66.47) Proportion of participants on anticoagulants and/or antiplatelets prior to surgery (n/N, %): anticoagulant: excluded; antiplatelet: not reported Incidence of preoperative anaemia: not reported Co‐morbidities: not reported ASA I (n/N,%): 0/20, 0% ASA II (n/N,%): 15/20, 75% ASA III (n/N,%): 5/20, 25% ASA IV (n/N,%): not reported Number of participants randomised: not reported Number of participants receiving treatment: 20 Number of participants analysed: 20 Dropout rate: not reported **TXA arm** Age (years) (mean SD): 65.11 (4.89) Gender (male, female): 14 M (77.8%), 4 F (22%) Length of surgery (min): 93.89 (16.94) Proportion of participants on anticoagulants and/or antiplatelets prior to surgery (n/N, %): anticoagulant: excluded; antiplatelet: not reported Incidence of preoperative anaemia: not reported Co‐morbidities: not reported ASA I (n/N,%): 0/18, 0% ASA II (n/N,%): 15/18, 83.3% ASA III (n/N,%): 3/18, 16.6% ASA IV (n/N,%): not reported Number of participants randomised: not reported Number of participants receiving treatment: 18 Number of participants analysed: 18 Dropout rate: not reported **Inclusion criteria** Patients aged between 20‐50 years (ASA grades I‐II) referring to Poursina Hospital, Rasht Iran Undergoing surgery for femoral fracture with intramedullary nailing **Exclusion criteria** Surgery took > 90 min Coronary artery disease History of arterial fibrillation Thrombophilia Chronic renal failure Hb < 10 g/dL Thromboembolic episodes (DVT or pulmonary embolus) Taking anticoagulant medication or oral contraceptive pills Allergy to TXA Presence of subarachnoid haemorrhage Pregnancy Breastfeeding **Tourniquet use:** not reported **Type of anaesthetic:** general **Type of surgery:** proximal femoral shaft fracture surgery with intra medullary nailing	
Interventions	**Placebo arm** Group II participants received identical volumes of normal saline for 10 min **TXA arm** Group I participants received 15 mg/kg IV TXA (Caspian, Iran) injections dissolved in 100 mL normal saline and 20 min before skin incision... for 10 min	
Outcomes	Intraoperative blood loss Need for transfusion haemoglobin and/or haematocrit level/change	
Notes	**Sponsorship source:** non‐pharmaceutical (This study was financially supported by Vice‐Chancellorship of research and technology of Guilan University of Medical Science). **Country:** Iran **Setting:** single‐centre **Comments**: there was no conflict of interest **Authors name:** M Haghighi **Institution:** Guilan University of Medical Sciences **Email:** mohaghighi@gums.ac.ir; a_sedighinejad@yahoo.com (corresponding author: Abbas Sedighinejad) **Address:** Anesthesiology Research Center, Poursina hospital, Guilan University of Medical Sciences, Rasht, Guilan, Iran **Native language of paper:** English **Reference type:** full text (1), trial registration (1) **Trial registration number:** IRCT201104256280N1 **Was it translated for this review:** no	
***Risk of bias***		
**Bias**	**Authors' judgement**	**Support for judgement**
Random sequence generation (selection bias)	Unclear risk	Quote: none Judgement comment: insufficient information to permit judgement, although.. ''Patients were allocated into two groups based on randomized block method''
Allocation concealment (selection bias)	Unclear risk	Quote: none Judgement comment: method of allocation concealment not described
Blinding of participants and personnel (performance bias) subjective outcomes	Unclear risk	Quote: none Judgement comment: referred to as a ''double blind randomised trial'' but no description of methods of blinding.
Blinding of participants and personnel (performance bias) objective outcomes	Low risk	Quote: none Judgement comment: referred to as a ''double blind randomised trial'' but no description of methods of blinding ‐ assumed to be participants and personnel
Blinding of outcome assessment (detection bias) subjective outcomes	Unclear risk	Quote: none Judgement comment: insufficient information to permit judgement (double‐blinding assumed to be referring to participants and personnel)
Blinding of outcome assessment (detection bias) objective outcomes	Low risk	Quote: none Judgement comment: no description given of outcome assessor blinding. Though method of blinding unreported, unlikely to affect objective outcomes
Incomplete outcome data (attrition bias) All outcomes	Low risk	Quote: none Judgement comment: no obvious outcome data missing. 40 people enrolled, 38 people analysed. Reasons for exclusion/dropout explained
Selective reporting (reporting bias)	Low risk	Quote: none Judgement comment: all outcomes planned in the protocol or prospective trial registration are reported.
Other bias	Low risk	Quote: none Judgement comment: no other concerns such as early stopping or imbalanced study arms

**Table:** Kashefi 2012

***Study characteristics***		
Methods	**Study design:** RCT **Length of duration of study:** not reported **Power calculation reached:** not reported **Transfusion strategy:** not reported **Was the trial stopped early:** not reported **Follow up:** NR	
Participants	**Baseline characteristics** **Placebo arm** Age (years) (mean SD): 39.5 (8.9) Gender (male, female): 33 M (82.5%), 7 F (17.5%) Length of surgery (min): not reported Proportion of participants on anticoagulants and/or antiplatelets prior to surgery (n/N, %): anticoagulant: excluded; antiplatelet: not reported Incidence of preoperative anaemia: not reported Co‐morbidities: not reported ASA I (n/N,%): not reported ASA II (n/N,%): not reported ASA III (n/N,%): not eligible ASA IV (n/N,%): not eligible Number of participants randomised: not reported Number of participants receiving treatment: not reported Number of participants analysed: 40 Dropout rate: not reported **TXA arm** Age (years) (mean SD): 43.2 (7.8) Gender (male, female): 31 M (77.5%), 9 F (22.5%) Length of surgery (min): not reported Proportion of participants on anticoagulants and/or antiplatelets prior to surgery (n/N, %): anticoagulant: excluded; antiplatelet: not reported Incidence of preoperative anaemia: not reported Co‐morbidities: not reported ASA I (n/N,%): not reported ASA II (n/N,%): not reported ASA III (n/N,%): not eligible ASA IV (n/N,%): not eligible Number of participants randomised: not reported Number of participants receiving treatment: not reported Number of participants analysed: 40 Dropout rate: not reported **Inclusion criteria** Patients aged 18‐64 years Candidates for femoral trunk surgery Without any history of coagulation disease Normality of coagulation tests that were referred to medical centres **Exclusion criteria** In case of change in anaesthesia or surgery and failure in regional anaesthesia, the patient was excluded from the study **Tourniquet use:** not reported **Type of anaesthetic:** spinal **Type of surgery:** femoral shaft surgery/femoral trunk surgery	
Interventions	**Placebo arm** Only normal saline (same volume as TXA); 1 h before the operation IV injection of 5 mL of liquid of the same colour, shape, with a similar syringe and a specific code **TXA arm** In TXA group, 15 mg/kg TXA (within 5 mL of liquid) via IV injection 1 h before the operation	
Outcomes	**Primary outcomes** Bleeding Need for transfusion Adverse events	
Notes	**Sponsorship source:** not reported **Country:** Iran **Setting:** not reported **Comments:** translation used, limited detail available. No relevant outcome extractable from translation which stated that "The use of 15 mg /kg TXA one hour before femoral shaft surgery reduces ... blood transfusions", the data were not available in the translated document. Unclear baseline characteristics: the translation refers to "first group" and "second group", assumed that "first group" = TXA, and "second group" = placebo, though this is not definite **Author's name:** P Kashefi **Institution:** Isfahan University of Medical Sciences **Email:** m_heidari@med.mui.ac.ir (Corresponding Author: Sayed Morteza Heidari) **Address:** Department of Anesthesiology and Intensive Care Unit, School of Medicine, Isfahan University of Medical Sciences, Isfahan, Iran **Native language of paper:** Persian (Farsi) **Reference type:** full text (1) **Trial registration number:** not reported **Was it translated for this review:** yes	
***Risk of bias***		
**Bias**	**Authors' judgement**	**Support for judgement**
Random sequence generation (selection bias)	Low risk	Quote: ''randomly divided into two groups of control and study using a table of random numbers" Judgement comment: none
Allocation concealment (selection bias)	Low risk	Quote: none Judgement comment: 1 h before the operation 5 mL of liquid of the same colour, shape, with a similar syringe and a specific code was injected into the participants by the first researcher.
Blinding of participants and personnel (performance bias) subjective outcomes	Unclear risk	Quote: none Judgement comment: described as double‐blind study. Researchers appear to be blinded to allocation as syringes were identical, but lacks detail
Blinding of participants and personnel (performance bias) objective outcomes	Low risk	Quote: none Judgement comment: described as double‐blind study. Unlikely to affect objective outcomes
Blinding of outcome assessment (detection bias) subjective outcomes	Unclear risk	Quote: none Judgement comment: no description given of outcome assessor blinding
Blinding of outcome assessment (detection bias) objective outcomes	Low risk	Quote: none Judgement comment: no description given of outcome assessor blinding. Though method of blinding unreported, unlikely to affect objective outcomes
Incomplete outcome data (attrition bias) All outcomes	Unclear risk	Quote: none Judgement comment: lack of detail regarding participant flow, dropout, and exclusions
Selective reporting (reporting bias)	High risk	Quote: none Judgement comment: no data presented for outcomes (number of transfusions), despite being mentioned as significantly difference as result of the intervention
Other bias	Unclear risk	Quote: none Judgement comment: lack of information on baseline characteristics

**Table:** Lei 2017

***Study characteristics***		
Methods	**Study design:** RCT **Length of duration of study:** 7 months (December 2015‐July 2016) + 1 month follow‐up **Power calculation reached:** yes (72 participants were needed, but 77 were analysed) **Transfusion strategy:** not reported **Was the trial stopped early:** no **Follow up:** 3 days	
Participants	**Baseline characteristics** **Placebo arm** Age (years) (mean SD): 79.18 (6.50) Gender (male, female): 7 M (17.5%), 33 F (82.5%) Length of surgery (min): 80.67 (29.44) Proportion of participants on anticoagulants and/or antiplatelets prior to surgery (n/N, %): anticoagulants: excluded 0/40, 0%; antiplatelets: excluded 0/40, 0% Incidence of preoperative anaemia: not reported Co‐morbidities: not reported ASA I (n/N,%): 4/40, 10% ASA II (n/N,%): 18/40, 45% ASA III (n/N,%): 18/40, 45% ASA IV (n/N,%): 1/40, 2.5% Number of participants randomised: 41 Number of participants receiving treatment: 41 Number of participants analysed: 40 Dropout rate: 0/41, 0% **TXA arm** Age (years) (mean SD): 77.80 (9.75) Gender (male, female): 5 M (13.5%), 32 F (86.5%) Length of surgery (min): 81.90 (25.61) Proportion of participants on anticoagulants and/or antiplatelets prior to surgery (n/N, %): anticoagulants: excluded 0/37, 0%; antiplatelets: excluded 0/37, 0% Incidence of preoperative anaemia: not reported Co‐morbidities: not reported ASA I (n/N,%): 5/37, 13.5% ASA II (n/N,%): 16/37, 43.2% ASA III (n/N,%): 16/37, 43.2% ASA IV (n/N,%): 2/37, 5.4% Number of participants randomised: 39 Number of participants receiving treatment: 39 Number of participants analysed: 37 Dropout rate: 0/39, 0% **Inclusion criteria** History of trauma, fall or traffic accident Hip pain, tenderness, dysfunction, local swelling, and vertical percussion pain in the area of the greater trochanter, with limited function in the injured limb Confirmed diagnosis of intertrochanteric fracture and fracture classified according to AO type on X‐ray or CT Eligible for intertrochanteric fracture surgery using the PFNA system (TianJin ZhengTian, XiaMen Double), as determined by the senior orthopedic surgeons **Exclusion criteria** Allergy to TXA Recent or ongoing thromboembolic events (DVT, PE, arterial thrombosis, or cerebral thrombosis stroke) Recently or currently taking anticoagulation therapy including vitamin K‐antagonists, direct thrombin inhibitors, direct factor X‐a inhibitors, and platelet aggregation inhibitors Disseminated intravascular coagulation or hepatic or renal diseases with impairment of coagulation function History of subarachnoid bleeding, malignancy, pathological fracture, or prior surgery on the injured hip **Tourniquet use:** not reported **Type of anaesthetic:** not reported **Type of surgery:** intertrochanteric fracture surgery using the PFNA system	
Interventions	**Placebo arm** 200 mL of IV NS (IV) **TXA arm** After anaesthesia, but before surgery, participants received 1 g IV TXA (200 mL)	
Outcomes	**Primary outcome** Postoperative hidden blood loss **Secondary outcomes** need for transfusion Hb and hematocrit levels 1 day before surgery and on postoperative days 1 and 3 Duration of surgery Visible blood loss Complications associated with surgery	
Notes	**Sponsorship source:** non‐pharmaceutical (this work was supported by the Science and Technology Project of Shaanxi Social Development (2016SF‐312)) **Country:** China **Setting:** single‐centre **Comments:** upon admission, the Hb level in 16 participants was < 90 g/L; these participants received a total of 48.0 U of packed RBC by IV infusion (pre‐op). **Authors name:** J Lei **Institution:** Xi’an Honghui Hospital, Xi’an Jiaotong University Health Science Center **Email:** lei7419@126.com (Y Zhuang); hhyyzk@126.com (K Zhang) **Address:** Department of Orthopaedic Trauma, Xi’an Honghui Hospital, Xi’an Jiaotong University Health Science Center, No 555, Youyi East Road, Xi’an, Shaanxi Province, China **Native language of paper:** English **Reference type:** full text (1), conference abstract (1), trial registration (1) **Trial registration number:** ChiCTR‐INR‐16008134 **Was it translated for this review:** no	
***Risk of bias***		
**Bias**	**Authors' judgement**	**Support for judgement**
Random sequence generation (selection bias)	Low risk	Quote: ''Patients were randomized to a TXA group or a normal‐ saline (NS) group using a random number table.'' Judgement comment: adequate method of sequence generation with computer–generated random numbers
Allocation concealment (selection bias)	Unclear risk	Quote: none Judgement comment: method of allocation concealment not described
Blinding of participants and personnel (performance bias) subjective outcomes	High risk	Quote: none Judgement comment: single‐blinded only (assumed to be patient‐blinded). No information regarding method of blinding
Blinding of participants and personnel (performance bias) objective outcomes	Low risk	Quote: none Judgement comment: insufficient information regarding blinding (single‐blinded only). Though method of blinding insufficiently reported, this is unlikely to affect objective outcomes
Blinding of outcome assessment (detection bias) subjective outcomes	Unclear risk	Quote: none Judgement comment: no description given of outcome assessor blinding
Blinding of outcome assessment (detection bias) objective outcomes	Low risk	Quote: none Judgement comment: no description given of outcome assessor blinding. Though method of blinding unreported, unlikely to affect objective outcomes
Incomplete outcome data (attrition bias) All outcomes	Low risk	Quote: none Judgement comment: participant flow reported; reasons for exclusion recorded. No obvious outcome data missing
Selective reporting (reporting bias)	Low risk	Quote: none Judgement comment: all outcomes planned in the protocol or prospective trial registration are reported. (More outcomes are given in the full text than in the protocol.)
Other bias	Low risk	Quote: none Judgement comment: no other concerns such as early stopping or imbalanced study arms

**Table:** Luo 2019

***Study characteristics***		
Methods	**Study design:** RCT **Length of duration of study:** 16 months (September 2015‐January 2017) + 6‐week follow‐up **Power calculation reached:** no (study underpowered to detect thrombotic events, enrolled 50 per arm (as per calculation); analysed 44‐46 per arm **Transfusion strategy:** blood transfusion administered if Hb was < 8 g/dL or if Hb was ≥8 g/dL, but there were signs of excess blood loss such as tachycardia, tachypnoea, or haemodynamic instability **Was the trial stopped early:** no **Follow up:** hospital stay, except mortality, CVA/stroke, and DVT (6 weeks)	
Participants	**Baseline characteristics** **Placebo arm** Age (years) (mean SD): 76.1 (9.3) Gender (male, female): 20 M (43.5%), 26 F (56.5%) Length of surgery (min): 62.5 (9.1) Proportion of participants on anticoagulants and/or antiplatelets prior to surgery (n/N, %): participants who were on anticoagulant therapy were not excluded; they were asked to stop anticoagulation therapy 5 days before the operation. Proportion of participants on antiplatelets not reported Incidence of preoperative anaemia: not reported Co‐morbidities: hypertension 20/46, 43.5%; diabetes 5/46, 10.9%; cardiac disease 13/46, 28.3%; neurological disease 11/46, 23.9%; pulmonary disease 3/46, 6.5% ASA I (n/N,%): 10/46, 21.7% ASA II (n/N,%): 16/46, 34.8% ASA III (n/N,%): 18/46, 39.1% ASA IV (n/N,%): 2/46, 4.3% Number of participants randomised: 50 Number of participants receiving treatment: 48 Number of participants analysed: 46 Dropout rate: 0/50, 0% **TXA arm** Age (years) (mean SD): 75.1 (8.0) Gender (male, female): 23 M (52.3%), 21 F (47.7%) Length of surgery (min): 60.4 (10.3) Proportion of participants on anticoagulants and/or antiplatelets prior to surgery (n/N, %): participants who were on anticoagulant therapy were not excluded; they were asked to stop anticoagulation therapy 5 days before the operation. Proportion of participants on antiplatelets not reported Incidence of preoperative anaemia: not reported Co‐morbidities: hypertension 18/44, 40.9%; diabetes 2/44, 4.5%; cardiac disease 10/44, 22.7%; neurological disease 10/44, 22.7%; pulmonary disease 2/44, 4.5% ASA I (n/N,%): 8/44, 18.2% ASA II (n/N,%): 19/44, 43.2% ASA III (n/N,%): 15/44, 34.1% ASA IV (n/N,%): 2/44, 4.5% Number of participants randomised: 50 Number of participants receiving treatment: 46 Number of participants analysed: 44 Dropout rate: 0/50, 0% **Inclusion criteria** Intertrochanteric fracture (extracapsular fractures of AO/OTA types 31‐A1 to 31‐A3) treated with PFNA Closed fracture with low‐energy damage Aged ≥ 60 years **Exclusion criteria** Preoperative examination revealed DVT Any contraindication for anticoagulation therapy A pathological fracture One of the following diseases in the preceding year: MI, cerebral infarction, coronary syndrome, DVT, or PE Duration from injury to operation was > 3 weeks Allergy to TXA Patients who had adverse drug reactions when using TXA and stopped the medication Multiple fractures, with the other fracture also needing surgical treatment Preoperative Hb < 8 g/dL Closed reduction failed, and therefore open reduction was performed There was any change in the fixation method or if, intraoperatively, the decision was made to perform arthroplasty *Patients who were on anticoagulant therapy were not excluded; they were asked to stop anticoagulation therapy 5 days before the operation. **Tourniquet use:** not reported **Type of anaesthetic:** general: n = 2 TXA, n = 2 control; spinal: n = 42 TXA, n = 44 control **Type of surgery:** PFNA	
Interventions	**Placebo arm** 100 mL IV saline 15 min before incision **TXA arm** 15 mg/kg body weight of IV TXA 15 min before incision and the same dose again 3 h later	
Outcomes	**Primary outcomes** Total perioperative blood loss Postoperative transfusion rate Postoperative Hb level Length of the hospital stay Occurrence of thrombotic events within 6 weeks after operation **Secondary outcomes** Mortality rate Adverse events related to TXA	
Notes	**Sponsorship source:** none **Country:** China **Setting**: multi‐centre **Comments:** none **Authors name:** X. Luo **Institution:** Zhujiang Hospital, Southern Medical University **Email:** qili565@foxmail.com **Address:** Department of Orthopaedic, Zhujiang Hospital, Southern Medical University, Guangzhou, China **Native language of paper:** English **Reference type:** full text (1), trial registration (1) **Trial registration number:** ChiCTR‐IPR‐15007122 **Was it translated for this review:** no	
***Risk of bias***		
**Bias**	**Authors' judgement**	**Support for judgement**
Random sequence generation (selection bias)	Low risk	Quote: ''Randomization was performed using a computer‐generated random number table'' Judgement comment: adequate method of sequence generation with computer–generated random numbers
Allocation concealment (selection bias)	Unclear risk	Quote: ''and sealed envelopes for the treatment allotment.'' Judgement comment: envelopes not described as sealed, opaque and sequentially numbered
Blinding of participants and personnel (performance bias) subjective outcomes	High risk	Quote: ''The patient and the investigator were blinded to the group allocation.'' Quote: "On the day of surgery, the anesthesiologist received the sealed envelope from the orthopaedic resident and administered the allotted drug". Judgement comment: therefore the resident becomes unblinded at the point of administering the drug and remains unblinded throughout the procedure.''
Blinding of participants and personnel (performance bias) objective outcomes	Low risk	Quote: ''The patient and the investigator were blinded to the group allocation.'' Quote: "On the day of surgery, the anesthesiologist received the sealed envelope from the orthopaedic resident and administered the allotted drug". Judgement comment: therefore the resident becomes unblinded at the point of administering the drug and remains unblinded throughout the procedure, but unlikely to affect objective outcomes.
Blinding of outcome assessment (detection bias) subjective outcomes	Unclear risk	Quote: none Judgement comment: blinding of outcome assessors not reported
Blinding of outcome assessment (detection bias) objective outcomes	Low risk	Quote: none Judgement comment: blinding of outcome assessors not reported. Though method of blinding unreported, unlikely to affect objective outcomes
Incomplete outcome data (attrition bias) All outcomes	Low risk	Quote: none Judgement comment: does not appear to be ITT analysis ‐ 2 in each group had incomplete data and so were excluded. However, this was only 4/90 excluded. No loss to follow‐up, or dropouts
Selective reporting (reporting bias)	Low risk	Quote: none Judgement comment: all outcomes planned in the protocol or prospective trial registration are reported.
Other bias	Low risk	Quote: none Judgement comment: no other concerns such as early stopping or imbalanced study arms

**Table:** Ma 2021

***Study characteristics***		
Methods	**Study design:** RCT (parallel) **Length of duration of study:** 13 months (September 2018‐September 2019) plus 3‐month follow‐up **Power calculation reached:** yes ‐ 51 per group required, 61 per group to allow for dropout: 62 and 63 randomised and analysed **Transfusion strategy**: pre‐op transfusion criterion: Hb < 80 g/L or symptomatic anaemia in a patient with Hb 80 g/L‐100 g/L **Was the trial stopped early:** no **Follow up:** 3 days	
Participants	**Baseline characteristics** **Placebo arm** Age (years) (mean, SD): 78.66 (6.95) Gender (male, female): 22 M (35.5%), 40 F (64.52%) Length of surgery (min): not reported Proportion of participants on anticoagulants and/or antiplatelets prior to surgery (n/N, %): this was an exclusion criterion Incidence of preoperative anaemia: not reported Co‐morbidities: not reported ASA I‐II: 20/62 ASA III‐IV: 42/62 Number of participants randomised: 62 Number of participants receiving treatment: 62 Number of participants analysed: 62 Dropout rate: 0/62, 0% **TXA (IV) arm** Age (years) (mean, SD): 78.05 (7.62) Gender (male, female): 21 M (33.3%), 42 F (66.67%) Length of surgery (min): not reported Proportion of participants on anticoagulants and/or antiplatelets prior to surgery (n/N, %): this was an exclusion criterion Incidence of preoperative anaemia: not reported Co‐morbidities: not reported ASA I‐II: 22/63 ASA III‐IV: 41/63 Number of participants randomised: 63 Number of participants receiving treatment: 63 Number of participants analysed: 63 Dropout rate 0/63, 0% **Inclusion criteria** Radiographic examination (CR, CT, MRI, etc.) confirmed the initial fresh intertrochanteric fracture of the femur Patients over 65 years old Injury time was close to 6 h **Exclusion criteria** Injury time > 6 h Open fractures, other parts of the body with hemorrhagic wounds, or other areas with bleeding disorders (such as gastrointestinal bleeding) Additional fresh fractures in other body parts Recent or ongoing thromboembolic events (DVT, PE, arterial thrombosis, or cerebral thrombosis stroke) Recently or currently taking anticoagulation therapy including vitamin K‐antagonists, direct thrombin inhibitors, direct factor X‐a inhibitors, and platelet aggregation inhibitors Disseminated intravascular coagulation or patients had hepatic or renal diseases with impairment of coagulation function Receiving conservative treatment Known TXA allergy or allergies **Tourniquet use:** not reported **Type of anaesthetic**: not reported **Type of surgery**: intertrochanteric fracture	
Interventions	**Placebo arm** 200 mL of NS (IV) immediately post‐traumatic admission (pre‐op) **TXA (IV) arm ** TXA (0.5 g; Ruiyang Pharmaceutical Co. Ltd. Shandong, China) 1 g (200 mL) immediately post‐traumatic admission (pre‐op)	
Outcomes	**Primary outcomes** Post‐traumatic hidden blood loss preoperative transfusion rate Hb drop Haematocrit change Incidence of DVT Incidence of PE **Secondary outcomes** Length of admission to operation Length of hospital stay Complications (cardiac infarction, ischaemics cerebral infarction, stroke, respiratory infection, renal failure)	
Notes	**Sponsorship source:** non‐pharma (National Natural Science Fund of China (NO. 81874002), Science and Technology Support Project of Sichuan Province (NO.2018SZ0159), Chongqing General Hospital Medical Science and Technology Innovation Fund Project (Y2020MSXM21), and Chongqing Yuzhong district Science and Technology Project (20150131)) **Country:** China **Setting:** hospital ‐ single centre **Comments:** none **Authors name:** Huixu Ma **Institution:** Chongqing General Hospital, China **Email:** Xi Liu (corresponding author) liuinsistence@163.com; liuxi7979@sina.com **Address:** Dept of Radiology, Chongqing Traditional Chinese Medicine Hospital, Chongqing 400022, China **Native language of paper:** English **Reference type:** full text (1), and trial registration (1) **Trial registration number:** ChiCTR1800017761 **Was it translated for this review:** no	
***Risk of bias***		
**Bias**	**Authors' judgement**	**Support for judgement**
Random sequence generation (selection bias)	Low risk	Quote: "patients were randomly allocated into two groups (TXA group: IV TXA; NS group: IV NS) based on a computer‐generated randomization list, which was generated with the use of Randomization.com. The randomization was prepared by a statistician who was not involved in this clinical trial". Judgement comment: randomisation by a computer‐generated list (randomization.com)
Allocation concealment (selection bias)	Low risk	Quote: "patients were randomly allocated into two groups (TXA group: IV TXA; NS group: IV NS) based on a computer‐generated randomization list, which was generated with the use of Randomization.com. The randomization was prepared by a statistician who was not involved in this clinical trial". Judgement comment: randomisation prepared by a statistician not involved in the clinical trial using a computer‐generated list
Blinding of participants and personnel (performance bias) subjective outcomes	Unclear risk	Quote: none: Judgement comment: blinding not mentioned throughout. Use of placebo (saline) suggests participant blinding, but not clear
Blinding of participants and personnel (performance bias) objective outcomes	Low risk	Quote: none Judgement comment: blinding not mentioned throughout. Use of placebo (saline) suggests participant blinding, but not clear. Unlikely to impact objective outcomes
Blinding of outcome assessment (detection bias) subjective outcomes	Unclear risk	Quote: none Judgement comment: blinding not mentioned throughout. use of placebo (saline) suggests participant blinding, but unclear whether outcome assessors or personnel were blinded
Blinding of outcome assessment (detection bias) objective outcomes	Low risk	Quote: none Judgement comment: blinding not mentioned throughout. use of placebo (saline) suggests participant blinding, but not clear. Unlikely to impact objective outcomes
Incomplete outcome data (attrition bias) All outcomes	Low risk	Quote: none Judgement comment: all who were randomised were analysed, with no dropouts
Selective reporting (reporting bias)	Low risk	Quote: none Judgement comment: trial registration checked. Primary outcome measures have been reported.
Other bias	Low risk	Quote: none Judgement comment: none noted

**Table:** Monsef Kasmaei 2019

***Study characteristics***		
Methods	**Study design:** RCT **Length of duration of study:** 6 months (January‐June 2018) + 72 h follow‐up **Power calculation reached:** not reported **Transfusion strategy:** not reported **Was the trial stopped early:** no **Follow up:** 24h, 48h, and 72 h.	
Participants	**Baseline characteristics** **Placebo arm** Age (years) (median, range): not reported Gender (male, female): 29 M (54.7%), 24 F (45.3%) Length of surgery (min): not reported Proportion of participants on anticoagulants and/or antiplatelets prior to surgery (n/N, %): anticoagulants: excluded, antiplatelets: not reported Incidence of preoperative anaemia: not reported Co‐morbidities: not reported ASA I (n/N,%): not reported ASA II (n/N,%): not reported ASA III (n/N,%): not reported ASA IV (n/N,%): not reported Number of participants randomised: not reported Number of participants receiving treatment: not reported Number of participants analysed: 53 Dropout rate: not reported **TXA arm** Age (years) (median, range): not reported Gender (male, female): 36 M (67.9%), 17 F (32.1%) Length of surgery (min): not reported Proportion of participants on anticoagulants and/or antiplatelets prior to surgery (n/N, %): anticoagulants: excluded, antiplatelets: not reported Incidence of preoperative anaemia: not reported Co‐morbidities: not reported ASA I (n/N,%): not reported ASA II (n/N,%): not reported ASA III (n/N,%): not reported ASA IV (n/N,%): not reported Number of participants randomised: not reported Number of participants receiving treatment: not reported Number of participants analysed: 53 Dropout rate: not reported **Inclusion criteria** 106 patients with pelvic trauma who referred to hospital in first 3 h after trauma with age ranging from 18‐60 years old enrolled in this study **Exclusion criteria** Died during the study History of anticoagulant drugs, oral contraceptive use Abnormal INR, PT and PTT range CVA MI Coagulopathy disorders TBI CPR Renal failure Smoking Opioids Diabetes Hypertension Pregnancy Breastfeeding Referred from other hospitals > 3 h after trauma **Tourniquet use:** not reported **Type of anaesthetic:** not reported **Type of surgery:** surgery for pelvic trauma	
Interventions	**Placebo arm** Serum 0.9% NS, IV injected; serum 0.9% NS or placebo **TXA arm** 1 g TXA, IV injected; 1 g IV TXA for loading dose and 3 doses per 8 h for the maintenance	
Outcomes	Blood loss assessed as Hemoglobin (Hb), Hematocrit (HCT), Pulse Rate (PR) and Blood Pressure (BP) was checked at admission, 24 h, 48 h and 72 h after admission.	
Notes	**Sponsorship source:** non‐pharmaceutical (Rasht University of Medical Sciences ‐ found from trial registration) **Country:** Iran **Setting:** single‐centre **Comments** No conflicts of interest Age range reported as 18‐60 years but group mean reported as 12 and 15, clearly incorrect therefore listed as not reported. **Authors name:** V Monsef Kasmaei **Institution:** Guilan university of Medical Sciences **Email:** dr.arsalan2010@gmail.com (Corresponding author: S.A. Naseri Alavi) **Address:** Department of Neurosurgery, Faculty of Medicine, Tabriz University of Medical Sciences, Tabriz, Iran **Native language of paper:** English **Reference type:** full text (1), conference abstract (1), trial registration (1) **Trial registration number:** IRCT20130710013947N7 **Was it translated for this review:** no	
***Risk of bias***		
**Bias**	**Authors' judgement**	**Support for judgement**
Random sequence generation (selection bias)	Unclear risk	Quote: none Judgement comment: method of sequence generation for randomisation not described
Allocation concealment (selection bias)	Unclear risk	Quote: none Judgement comment: envelopes not described as sealed, opaque and sequentially numbered
Blinding of participants and personnel (performance bias) subjective outcomes	Low risk	Quote: none Judgement comment: trial registration states that participant, care provider, investigator and outcome assessor were masked, though unclear of method of blinding
Blinding of participants and personnel (performance bias) objective outcomes	Low risk	Quote: ''The syringes of TXA and N.S were blindly and intravenously injected to the patients by other nurse.'' Judgement comment: objective outcome for personnel and low risk of bias due to blinding. Trial registration states that participant, care provider, investigator and outcome assessor were masked, though unclear of method of blinding
Blinding of outcome assessment (detection bias) subjective outcomes	Unclear risk	Quote: none Judgement comment: no description given of outcome assessor blinding, although trial registration says they were masked. Whether this happened or not is unclear.
Blinding of outcome assessment (detection bias) objective outcomes	Low risk	Quote: none Judgement comment: objective outcome for personnel and low risk of bias due to blinding. Trial registration states that participant, care provider, investigator and outcome assessor were masked, though unclear of method of blinding
Incomplete outcome data (attrition bias) All outcomes	High risk	Quote: none Judgement comment: unclear participant flow. We know 106 (53 per group) were analysed, but no information regarding enrolment and randomisation numbers. Study authors state 56 intervention syringes were prepared and the remainder were saline (50), but then tables say 53 per group.
Selective reporting (reporting bias)	Low risk	Quote: none Judgement comment: all outcomes planned in the protocol or prospective trial registration are reported.
Other bias	Low risk	Quote: none Judgement comment: no other concerns such as early stopping or imbalanced study arms.

**Table:** NCT01727843

***Study characteristics***		
Methods	**Study design:** RCT, parallel, 2 arms **Length of duration of study:** 4 years, 7 months (April 2013‐November 2017) **Power calculation reached:** not reported **Transfusion strategy:** not reported **Was the trial stopped early:** yes (terminated) **Follow up:** 8 days	
Participants	**Baseline characteristics** **Placebo arm ** Age (years) (mean, SD): not reported Gender (male, female): not reported Length of surgery (min) (mean SD): not reported Proportion of participants on anticoagulants and/or antiplatelets prior to surgery (n/N, %): not reported Incidence of preoperative anaemia: not reported Co‐morbidities: not reported ASA I (n/N,%): not reported ASA II (n/N,%): not reported ASA III: (n/N,%): not reported ASA IV: (n/N,%): not reported Number of participants randomised: not reported Number of participants receiving treatment: not reported Number of participants analysed: not reported Dropout rate: not reported **TXA arm** Age (years) (mean, SD): not reported Gender (male, female): not reported Length of surgery (min) (mean SD): not reported Proportion of participants on anticoagulants and/or antiplatelets prior to surgery (n/N, %): not reported Incidence of preoperative anaemia: not reported Co‐morbidities: not reported ASA I (n/N,%): not reported ASA II (n/N,%): not reported ASA III: (n/N,%): not reported ASA IV: (n/N,%): not reported Number of participants randomised: not reported Number of participants receiving treatment: not reported Number of participants analysed: not reported Dropout rate: not reported **Inclusion criteria** Hip fracture patients Aged ≥ 65 **Exclusion criteria** Bilateral femoral neck fracture or fracture that is not suited to a hemiarthroplasty repair, or both **Tourniquet use:** not reported **Type of anaesthetic:** not reported **Type of surgery:** femoral neck fractures	
Interventions	**TXA (topical) arm ** 3000 mg/mL TXA in saline applied directly to the wound at the end of the surgical procedure **Placebo (saline, topical) arm** 3000 mg/mL saline applied directly to the wound at the end of the surgical procedure	
Outcomes	**Primary outcome** Blood loss (up to 8 days) **Secondary outcome** None	
Notes	**Sponsorship source:** non‐pharmaceutical **Country:** Canada **Setting:** single‐centre **Comments**: this trial was terminated (no reason given), but 15 participants were recruited. There has been no response to the multiple emails that have been sent to the author requesting use of the data gathered for these participants. Last update posted: 3 November 2018 **Authors name**/**Contact:** Principal Investigator: Jeff Yach, MD **Institution:** Queen's University **Email:** not reported **Address:** Queen's University, KGH, Kingston, Ontario, Canada, K7L2G7 **Native language of paper:** not applicable **Reference type:** trial registration (1) **Trial registration number:** NCT01727843 **Was it translated for this review:** no	
***Risk of bias***		
**Bias**	**Authors' judgement**	**Support for judgement**
Random sequence generation (selection bias)	Unclear risk	Quote: none Judgement comment: trial registration information only, no results or publications available (trial terminated after recruitment of 15 participants only)
Allocation concealment (selection bias)	Unclear risk	Quote: none Judgement comment: trial registration information only, no results or publications available (trial terminated after recruitment of 15 participants only)
Blinding of participants and personnel (performance bias) subjective outcomes	Unclear risk	Quote: none Judgement comment: trial registration information only, no results or publications available (trial terminated after recruitment of 15 participants only) Described as double‐blind in trial registration, no information on how blinding was implemented As method of blinding is insufficiently reported, an assessment of unclear is given for subjective outcomes.
Blinding of participants and personnel (performance bias) objective outcomes	Low risk	Quote: none Judgement comment: trial registration information only, no results or publications available (trial terminated after recruitment of 15 participants only) Described as double‐blind in trial registration, no information on how blinding was implemented. Unlikely to impact mortality. Though method of blinding unreported, unlikely to affect objective outcomes
Blinding of outcome assessment (detection bias) subjective outcomes	Unclear risk	Quote: none Judgement comment: trial registration information only, no results or publications available (trial terminated after recruitment of 15 participants only)
Blinding of outcome assessment (detection bias) objective outcomes	Low risk	Quote: none Judgement comment: trial registration information only, no results or publications available (trial terminated after recruitment of 15 participants only). Though method of blinding unreported, unlikely to affect objective outcomes.
Incomplete outcome data (attrition bias) All outcomes	Unclear risk	Quote: none Judgement comment: trial registration information only, no results or publications available (trial terminated after recruitment of 15 participants only)
Selective reporting (reporting bias)	Unclear risk	Quote: none Judgement comment: trial registration information only, no results or publications available (trial terminated after recruitment of 15 participants only)
Other bias	Unclear risk	Quote: none Judgement comment: trial registration information only, no results or publications available (trial terminated after recruitment of 15 participants only) Original estimated enrolment: 126; terminated at 15 recruited (November 2018)

**Table:** NCT02664909

***Study characteristics***		
Methods	**Study design:** RCT **Length of duration of study:** not reported, though report stated "Patients will be in the study for 4 to 6 weeks" and the expected timetable was 2 years **Power calculation reached:** no **Transfusion strategy:** not reported **Was the trial stopped early:** no **Follow up: ** 4 days, except complications (4‐6 weeks)	
Participants	**Baseline characteristics** **Placebo arm ** Age (years) (mean, SD) 83 (9.6) Gender (male, female): 2 M (10.5%), 17 F (89.5%) Length of surgery (min) (mean SD): not reported Proportion of participants on anticoagulants and/or antiplatelets prior to surgery (n/N, %): anticoagulants were excluded, antiplatelets data not reported Incidence of preoperative anaemia: not reported Co‐morbidities: not reported ASA I (n/N,%): not reported ASA II (n/N,%): not reported ASA III: (n/N,%): not reported ASA IV: (n/N,%): not reported Number of participants randomised: not reported Number of participants receiving treatment: not reported Number of participants analysed: 19 Dropout rate: not reported **TXA arm** Age (years) (mean, SD) 83 (8.6) Gender (male, female): 3 M (17.6%), 14 F (82.4%) Length of surgery (min) (mean SD): not reported Proportion of participants on anticoagulants and/or antiplatelets prior to surgery (n/N, %): anticoagulants were excluded, antiplatelets data not reported Incidence of preoperative anaemia: not reported Co‐morbidities: not reported ASA I (n/N,%): not reported ASA II (n/N,%): not reported ASA III: (n/N,%): not reported ASA IV: (n/N,%): not reported Number of participants randomised: not reported Number of participants receiving treatment: not reported Number of participants analysed: 17 Dropout rate: not reported **Inclusion criteria** Hip hemiarthroplasty surgery for a displaced femoral neck fracture Age ≥ 55 years **Exclusion criteria** History of haemophilia, DVT, PE, thrombophilia, or chronic renal failure Coronary ischaemia (active or within the past calendar year) MI Previous percutaneous coronary intervention, or coronary artery bypass grafting Any revascularisation procedure within the past calendar year Active subarachnoid haemorrhage Acquired defective colour vision A pathologic fracture (fracture through a neoplastic lesion) Pregnant Known allergy to TXA Taking warfarin, dabigatran, rivaroxaban, apixaban, or FFP **Tourniquet use:** not reported **Type of anaesthetic:** not reported **Type of surgery:** hip hemiarthroplasty surgery (femoral neck fractures)	
Interventions	**Placebo arm** 50 mL of topically applied normal saline into surgical wound at the time of wound closure. Half of this 50 mL dose of normal saline was delivered intra‐articularly and half was delivered in the subfascial space. **TXA arm** 1 g of topically applied TXA into surgical wound at the time of wound closure	
Outcomes	**Primary outcome** Number of participants who needed transfusions **Secondary outcomes** Inpatient transfusion amount Difference between pre/post‐operative Hb Difference between pre/post‐operative haematocrit Length of inpatient hospital stay Number of participants with post‐operative complications Inpatient hospitalisation cost	
Notes	**Sponsorship source:** non‐pharma (UConn Health; Orthopaedic Research and Education Foundation) **Country:** USA **Setting:** single centre ‐ hospital **Comments:** data and risk of bias assessment based on trial registration uploaded results only. Not peer‐reviewed **Authors name:** Vincent Williams, MD **Institution:** University of Connecticut Health Center **Email:** vwilliams@uchc.edu **Address:** University of Connecticut Health Center 263 Farmington Ave. Farmington, CT. 06030 **Native language of paper:** English **Reference type**: trial registration (1) document with full study outcome data provided in the results tab **Trial registration number:** NCT02664909 (clinical trials.gov) **Was it translated for this review:** not applicable	
***Risk of bias***		
**Bias**	**Authors' judgement**	**Support for judgement**
Random sequence generation (selection bias)	Low risk	Quote: "Prior to the start of the study, a randomization schedule will be constructed with block randomization using web‐based software. " Judgement comment: none
Allocation concealment (selection bias)	Low risk	Quote: "Group assignments will be concealed in opaque sealed envelopes with a numerical code of consecutive numbers reflecting patient enrollment. Envelopes will be stored with the pharmacy staff in charge of drug preparation. The pharmacy staff, which will have no patient contact, will remain un‐blinded. The pharmacy staff will maintain the master key identifying which patients received the study drug and which patients received the placebo. Physicians, residents, hospital staff, and patients will be blinded to group assignment. " Judgement comment: none
Blinding of participants and personnel (performance bias) subjective outcomes	Low risk	Quote: none Judgement comment: protocol describes study as double‐blind, and trial registration describes it as quadruple‐blind
Blinding of participants and personnel (performance bias) objective outcomes	Low risk	Quote: none Judgement comment: protocol describes study as double‐blind, and trial registration describes it as quadruple‐blind
Blinding of outcome assessment (detection bias) subjective outcomes	Low risk	Quote: none Judgement comment: protocol describes study as double‐blind, and trial registration describes it as quadruple‐blind
Blinding of outcome assessment (detection bias) objective outcomes	Low risk	Quote: none Judgement comment: protocol describes study as double‐blind, and trial registration describes it as quadruple‐blind
Incomplete outcome data (attrition bias) All outcomes	High risk	Quote: none Judgement comment: insufficient information to make a judgement: expected (and required by power calculation) to recruit 102 total, but they report only 36 "enrolled". Data given for number started (36), number completed (31), and number not completed (5). 36 analysed. Statistical protocol does not state whether they would use ITT or PP (per protocol). No information regarding reason for limited recruitment
Selective reporting (reporting bias)	High risk	Quote: none Judgement comment: history of changes in trial registration show that the primary outcome has been changed from Transfusion rate (2016) to Number of patients who required transfusion (2021).
Other bias	High risk	Quote: none Judgement comment: based on trial registration information and uploaded results only (not peer‐reviewed). No publications located. Sponsors noted in trial registration. No baseline imbalance noted

**Table:** Parish 2021

***Study characteristics***		
Methods	**Study design:** RCT (parallel) **Length of duration of study:** not reported **Power calculation reached:** 30 per arm required, assume 30 per arm analysed, but this is unclear (study reports baseline characteristics for 30 participants per arm) **Transfusion strategy:** not reported **Was the trial stopped early:** no **Follow up:** 24‐48 hours, except complications (3 weeks)	
Participants	**Baseline characteristics** **Placebo arm** Age (years) (mean, SD): 47.40 (12.55) Gender (male, female): 23 M (76.6%), 7 F (23.3%) Length of surgery (min) (mean SD): not reported Proportion of participants on anticoagulants and/or antiplatelets prior to surgery (n/N, %): this was an exclusion criterion Incidence of preoperative anaemia: not reported Co‐morbidities: not reported ASA I (n/N,%): not reported ASA II (n/N,%): not reported ASA III: (n/N,%): not reported ASA IV: (n/N,%): not reported Number of participants randomised: not reported Number of participants receiving treatment: not reported Number of participants analysed: 30 Dropout rate: not reported **TXA arm** Age (years) (mean, SD) 43.77 (15.65) Gender (male, female) 22 M (73.3%), 8 F (26.7%) Length of surgery (min) (mean SD): not reported Proportion of participants on anticoagulants and/or antiplatelets prior to surgery (n/N, %): this was an exclusion criterion Incidence of preoperative anaemia: not reported Co‐morbidities: not reported ASA I (n/N,%): not reported ASA II (n/N,%): not reported ASA III (n/N,%): not reported ASA IV (n/N,%): not reported Number of participants randomised: not reported Number of participants receiving treatment: not reported Number of participants analysed: 30 Dropout rate: not reported **Inclusion criteria** At least 18 years old Candidates for femoral fracture surgery with concher insertion Physical classes 1 and 2 based on ASA T Type, transverse and associated acetabular fracture **Exclusion criteria** Sensitivity to TXA Pre‐existing anaemia Avoidance of blood transfusion History of anticoagulant drugs Coagulation disorders History of thromboembolism, cerebrovascular damage or seizures Ischaemic heart disease Pregnancy Major underlying diseases (pulmonary or heart disease, liver failure, renal failure Need for re‐surgery **Tourniquet use:** not reported **Type of anaesthetic:** anaesthesia induced after regular checks. Indications were propofol, fentanyl, midzolam, atracurium. Therefore, assumed to be general anaesthesia **Type of surgery:** concher femoral insertion surgery	
Interventions	**Placebo arm ** NS (10 mg/kg) 15 min before infusion **TXA (IV) arm ** TXA IV, 10 mg/kg 15 min before infusion, then infusion at 1 mg/kg/h until end of surgery	
Outcomes	Hb level Incidence of DVT Bleeding volume Need for blood transfusion	
Notes	**Sponsorship source:** non‐pharmaceutical (This study is sponsored by Tabriz University of Medical Sciences) **Country:** Iran **Setting:** Single centre ‐ hospital **Comments:** none **Authors name:** M. Parish **Institution:** Tabriz University of Medical Sciences **Email:** corresponding author: Naghi Abedini. naghi26@yahoo.com **Address:** Dept of Anaesthesiology, School of Medicine, Tabriz, Iran **Native language of paper:** English **Reference type:** full text (1), trial registration (1) **Trial registration number:** Iranian Registry Of clinical Trials (NO: IRCT20191208045664N1). **Was it translated for this review:** no	
***Risk of bias***		
**Bias**	**Authors' judgement**	**Support for judgement**
Random sequence generation (selection bias)	Low risk	Quote: "subjects were allocated to the groups of intervention and control using the random block with sizes of 2 and 4. A random sequence was generated using the RAS software" Judgement comment: none
Allocation concealment (selection bias)	Low risk	Quote: none Judgement comment: the main researcher and statistical advisor were not aware of the allocation of participants, and data were collected by the assistant researcher.
Blinding of participants and personnel (performance bias) subjective outcomes	Low risk	Quote: none Judgement comment: drugs were prepared in similar syringes and delivered to the anaesthesiologist who was unaware of the contents. Syringes were coded. Described as double‐blind trial
Blinding of participants and personnel (performance bias) objective outcomes	Low risk	Quote: none Judgement comment: drugs were prepared in similar syringes and delivered to the anaesthesiologist who was unaware of the contents. Syringes were coded. Described as double‐blind trial
Blinding of outcome assessment (detection bias) subjective outcomes	Unclear risk	Quote: none Judgement comment: described as double‐blind trial (likely referring to participants and personnel only). The main researcher and statistical advisor were not aware of allocation, (but) the data were collected by the assistant researcher (no mention if they were blinded)
Blinding of outcome assessment (detection bias) objective outcomes	Low risk	Quote: none Judgement comment: described as double‐blind trial (likely referring to participants and personnel only). The main researcher and statistical advisor were not aware of allocation, (but) the data were collected by the assistant researcher (no mention if they were blinded) Unlikely to affect objective outcomes
Incomplete outcome data (attrition bias) All outcomes	Unclear risk	Quote: none Judgement comment: number randomised and analysed unclear. Assumption that 30 per group as gender count per group was given, and power calculation stated they needed 30 per group (to allow for attrition), but no clear indication to participant flow
Selective reporting (reporting bias)	High risk	Quote: none Judgement comment: primary outcomes poorly reported (e.g. DVT and Hb level). Trial registration states outcomes to be presented at 24 and 48 h post‐surgery, but only presented as "during" and "after". Very little detail (including lack of N per group)
Other bias	High risk	Quote: none Judgement comment: detail varies between trial registration and publication with regards to description of intervention, with no reference to changes or explanation for differing descriptions. Baseline imbalance in urinary extraversion ‐ this has been detailed extensively. Unclear how it would affect outcomes

**Table:** Raobaikady 2005

***Study characteristics***		
Methods	**Study design:** RCT **Length of duration of study:** 19 months (August 2002‐March 2004 ) + 7‐day follow‐up and also day 30 post‐op **Power calculation reached:** yes (48 participants were included in the trial to achieve 80% power at a 5% significance level). **Transfusion strategy:** allogeneic RBC were transfused when Hb was < 8.0 g/dL; platelets when platelet count < 100 x 10^9^/L, FFP when PT‐INR or APTT was > 1.5 times normal; and cryoprecipitate when fibrinogen concentration was < 0.8 g/L. In addition, intraoperative salvaged RBC were retransfused to every participant. **Was the trial stopped early:** no **Folllow up:** 30 days	
Participants	**Baseline characteristics** **Placebo arm** Age (years) (median, range): 38 (18‐57) Gender (male, female): 18 M (75%), 6 F (25%) Length of surgery (min) (median, range): 189 (115‐360) Proportion of participants on anticoagulants and/or antiplatelets prior to surgery (n/N, %): anticoagulant (received prophylactic anticoagulation with low molecular weight heparin and warfarin before and after surgery): 24/24, 100%; antiplatelets: not reported Incidence of preoperative anaemia: not reported Co‐morbidities: not reported ASA I (n/N,%): not reported ASA II (n/N,%): not reported ASA III (n/N,%): not reported ASA IV (n/N,%): not reported Number of participants randomised: 24 Number of participants receiving treatment: 24 Number of participants analysed: 24 Dropout rate: 0/24, 0% **rFVIIa arm** Age (years) (median, range): 44 (18–57) Gender (male, female): 16 M (67%), 8 F (33%) Length of surgery (min) (median, range): 177 (103–320) Proportion of participants on anticoagulants and/or antiplatelets prior to surgery (n/N, %): anticoagulant (received prophylactic anticoagulation with low molecular weight heparin and warfarin before and after surgery): 24/24, 100%; antiplatelets: not reported Incidence of preoperative anaemia: not reported Co‐morbidities: not reported ASA I (n/N,%): not reported ASA II (n/N,%): not reported ASA III (n/N,%): not reported ASA IV (n/N,%): not reported Number of participants randomised: 24 Number of participants receiving treatment: 24 Number of participants analysed: 24 Dropout rate: 0/24, 0% **Inclusion criteria** 18–60 years old Major pelvic–acetabular fracture caused by trauma Scheduled for semi‐elective ‘large’ reconstruction surgery with the potential of blood loss exceeding 50% of circulating blood volume **Exclusion criteria** History of thrombosis (DVT, PE, cerebral thrombosis) Severe head injuries or an abnormal CT scan of the head due to head injuries Base deficit of > 15 mEq/L or severe acidosis (pH < 7.0.) before surgery Body weight > 135 kg Known or suspected allergy to any drug that might be administered during the course of the study Cardiac arrest after trauma and before surgery at St George’s Hospital Known congenital bleeding disorders Known pregnancy or positive pregnancy test at enrolment Previous participation in this study Previous receipt of rFVIIa within 48 h of screening Currently participating or having participated in another investigational drug study within the last 30 days. **Tourniquet use:** not reported **Type of anaesthetic:** general **Type of surgery:** semi‐elective ‘large’ reconstruction surgery or major pelvic–acetabular surgery	
Interventions	**Placebo arm** Placebo was given IV as a bolus at the first skin incision. A second injection of the same dose was given 2 h after the first dose if the transfusion of allogeneic RBC was indicated by an intraoperative measurement of Hb concentration of < 8.0 g/dL after the retransfusion of salvaged RBC **rFVIIa** rFVIIa (NovoSeven; Novo Nordisk, Bagsvaerd,Denmark) 90 µg/kg was given IV as a bolus at the first skin incision. A second injection of the same dose was given 2 h after the first dose if the transfusion of allogeneic RBC was indicated by an intraoperative measurement of Hb concentration of < 8.0 g/dL after the retransfusion of salvaged RBC	
Outcomes	**Primary outcome** Total volume of perioperative blood loss **Secondary outcomes** Volumes of intraoperative and postoperative blood loss Volume of blood transfused during the perioperative period Vital signs Adverse events	
Notes	**Sponsorship source:** pharmaceutical (Novo Nordisk, UK) **Country:** UK **Setting:** single‐centre **Comments** Conflicts declared: RM Grounds has worked in the past as a consultant for Novo Nordisk and has lectured at symposiums organised by Novo Nordisk. Novo Nordisk has given an unrestricted educational grant to St George’s Hospital Special Trustees **Author's name:** R Raobaikady **Institution:** St George’s Hospital **Email:** michael.grounds@stgeorges.nhs.uk (Corresponding author: RM Grounds) **Address:** Department of Anaesthesia and Intensive Care Medicine, St George’s Hospital, Blackshaw Road, London SW17 0QT, UK **Native language of paper:** English **Reference type:** full text (1), conference abstract (1), trial registration (1) **Trial registration number:** NCT01601457 **Was it translated for this review:** no	
***Risk of bias***		
**Bias**	**Authors' judgement**	**Support for judgement**
Random sequence generation (selection bias)	Low risk	Quote: ''computer‐generated 1 to 1 randomization scheme.'' Judgement comment: adequate method of sequence generation with computer–generated code
Allocation concealment (selection bias)	Unclear risk	Quote: none Judgement comment: method of allocation concealment not described
Blinding of participants and personnel (performance bias) subjective outcomes	Unclear risk	Quote: none Judgement comment: no description given of participant or personnel blinding in full text. Although trial registration says participant and investigator were blinded
Blinding of participants and personnel (performance bias) objective outcomes	Low risk	Quote: none Judgement comment: masking: double (participant, investigator), though method of blinding unreported, unlikely to affect objective outcomes
Blinding of outcome assessment (detection bias) subjective outcomes	Unclear risk	Quote: none Judgement comment: not reported (not listed as masked/blinded)
Blinding of outcome assessment (detection bias) objective outcomes	Low risk	Quote: none Judgement comment: not reported (not listed as masked/blinded), though method of blinding unreported, unlikely to affect objective outcomes
Incomplete outcome data (attrition bias) All outcomes	Low risk	Quote: none Judgement comment: participant flow reported, no dropouts or loss to follow‐up
Selective reporting (reporting bias)	Low risk	Quote: none Judgement comment: all outcomes planned in the protocol or prospective trial registration are reported
Other bias	Low risk	Quote: none Judgement comment: no other concerns such as early stopping or imbalanced study arms

**Table:** Sadeghi 2007

***Study characteristics***		
Methods	**Study design:** RCT **Length of duration of study:** 16 months (February 2004‐June 2005) + 6‐week follow‐up **Power calculation reached:** not reported **Transfusion strategy:** transfusions were given on a case‐by‐case basis with regard to age, cardiovascular status, Hb concentration and blood loss. Most participants who had blood transfusions received these at a Hb concentration between 80 g/L and 100 g/L. **Was the trial stopped early:** no **Follow up:** 7 days	
Participants	**Baseline characteristics** **Placebo arm** Age (years) (mean SD): 44.4 (26.16) Gender (male, female): 24 M, 11 F (worked out from ratio of 2.18) Length of surgery (min): not reported Proportion of participants on anticoagulants and/or antiplatelets prior to surgery (n/N, %): anticoagulants: not reported; antiplatelets: not reported Incidence of preoperative anaemia: not reported Co‐morbidities: not reported ASA I (n/N,%): not reported ASA II (n/N,%): not reported ASA III (n/N,%): not reported ASA IV (n/N,%): not reported Number of participants randomised: 35 Number of participants receiving treatment: 35 Number of participants analysed: 35 Dropout rate: 0/35, 0% **TXA arm** Age (years) (mean SD): 51.81 (25.7) Gender (male, female): 17M, 15F (worked out from ratio of 1.13) Length of surgery (min): not reported Proportion of participants on anticoagulants and/or antiplatelets prior to surgery (n/N, %): anticoagulants: not reported; antiplatelets: not reported Incidence of preoperative anaemia: not reported Co‐morbidities: not reported ASA I (n/N,%): not reported ASA II (n/N,%): not reported ASA III (n/N,%): not reported ASA IV (n/N,%): not reported Number of participants randomised: 32 Number of participants receiving treatment: 32 Number of participants analysed: 32 Dropout rate: 0/32, 0% **Inclusion criteria** Consecutive hip fracture patients with extracapsular fractures treated by plating and nailing, and intracapsular fractures, treated by hemiarthroplasty **Exclusion criteria** Undisplaced subcapital fracture treated by pinning that have long been shown to be fractures with low level loss of blood Preoperative Hb < 10 g/L, platelet count < 100 × 10^9^/L of blood Known coagulopathies Renal insufficiency (creatinine > 2 mg/dL) Advanced hepatic dysfunction History of thromboemboli **Tourniquet use:** not reported **Type of anaesthetic:** spinal **Type of surgery:** consecutive hip fractured patients with extracapsular fractures treated by plating and nailing, and intracapsular fractures, treated by hemiarthroplasty.	
Interventions	**Placebo arm** Saline solution (15 mg/kg) was infused **TXA arm** In the TXA group, in an identical volume, a single bolus dose of 15 mg/kg was administered IV at induction of anaesthesia	
Outcomes	Post‐operative bleeding Need for allogeneic transfusion adverse events	
Notes	**Sponsorship source:** not reported **Country:** Iran **Setting:** single‐centre **Comments:** no conflicts of interest **Authors name:** M. Sadeghi **Institution:** Shariati Hospital **Email:** madani_68@yahoo.com (A. Mehr‐Aein: corresponding author) **Address:** Department of Anesthesiology, Shariati Hospital, School of Medicine, Tehran University of Medical Sciences, Tehran, Iran **Native language of paper:** English **Reference type:** full text (1) **Trial registration number:** not applicable (pre‐2010) **Was it translated for this review:** no	
***Risk of bias***		
**Bias**	**Authors' judgement**	**Support for judgement**
Random sequence generation (selection bias)	Low risk	Quote: ''Patients were randomized using a random number technique.'' Judgement comment: adequate method of sequence generation with computer–generated random numbers
Allocation concealment (selection bias)	Low risk	Quote: ''the correct treatment option was assured by means of coded infusion syringes, prepared by a personal of the hospital pharmacy, not involved otherwise in the study.'' Judgement comment: adequate method of central allocation concealment by pharmacy
Blinding of participants and personnel (performance bias) subjective outcomes	Low risk	Quote: none Judgement comment: caring personnel, both the staff of the operating room and the ICU, were blinded regarding the type and nature of treatment; the correct treatment option was assured by means of coded infusion syringes, prepared by hospital pharmacy personnel, not involved otherwise in the study. Subjective outcome for personnel, so low risk of bias regardless of quality of blinding
Blinding of participants and personnel (performance bias) objective outcomes	Low risk	Quote: none Judgement comment: caring personnel, both the staff of the operating room and the ICU, were blinded regarding the type and nature of treatment; the correct treatment option was assured by means of coded infusion syringes, prepared by hospital pharmacy personnel, not involved otherwise in the study. Objective outcome for personnel, so low risk of bias regardless of quality of blinding.
Blinding of outcome assessment (detection bias) subjective outcomes	Unclear risk	Quote: none Judgement comment: double‐blind study ‐ no information regarding outcome assessors
Blinding of outcome assessment (detection bias) objective outcomes	Low risk	Quote: none Judgement comment: double‐blind study ‐ no information regarding outcome assessors; unlikely to affect objective outcomes
Incomplete outcome data (attrition bias) All outcomes	Low risk	Quote: none Judgement comment: report 67 participants recruited, and 67 analysed. No dropouts reported
Selective reporting (reporting bias)	Unclear risk	Quote: none Judgement comment: no available prospective protocol or trial registration
Other bias	Low risk	Quote: none Judgement comment: no baseline imbalance or other sources of bias noted

**Table:** Zhang 2020a

***Study characteristics***		
Methods	**Study design:** RCT **Length of duration of study:** 16 months: 13 months (September 2018‐October 2019) + 3 months (90‐day follow‐up) **Power calculation reached:** yes ‐ included 61 per group (46 per group required) **Transfusion strategy:** blood losses were replaced with crystalloid solution in a 3:1 ratio, colloidal solution in a 1:1 ratio, or both until Hb concentration fell below the transfusion trigger point. The erythrocyte transfusion trigger point was set at a Hb level of < 70 g/L or 70 g/L–100 g/L with symptomatic anaemia (defined as light‐headedness, fatigue, palpitations, or shortness of breath not due to other causes) for each participant in accordance with the National Ministry of Health guidelines. **Was the trial stopped early:** no **Follow up:** 7 days, except complications (90 days)	
Participants	**Baseline characteristics** **Placebo arm** Age (years) (mean SD): 76.07 (16.60) Gender (male, female): 34 M (55.7%), 27 F (44.3%) Length of surgery (surgical time) (min) (mean SD): 81.38 (23.43) Proportion of participants on anticoagulants and/or antiplatelets prior to surgery (n/N, %): anticoagulants: not reported; antiplatelets: not reported Incidence of preoperative anaemia: not reported Co‐morbidities: hypertension 15/61, (24.6%); diabetes 3/61, (4.9%); heart disease 9/61, (14.8%); COPD 10/61, (16.4%) ASA I (n/N,%): 0/61, 0% ASA II (n/N,%): 12/61, 19.7% ASA III (n/N,%): 49/61, 80.3% ASA IV (n/N,%): 0/61, 0% Number of participants randomised: 61 Number of participants receiving treatment: 61 Number of participants analysed: 61 Dropout rate: 0/61, 0% **TXA arm** Age (years) (mean SD): 79.11 (11.91) Gender (male, female): 28 M (45.9%), 33 F (54.1%) Length of surgery (surgical time) (min) (mean SD): 80.13 (18.88) Proportion of participants on anticoagulants and/or antiplatelets prior to surgery (n/N, %): anticoagulants: not reported; antiplatelets: not reported Incidence of preoperative anaemia: not reported Co‐morbidities: hypertension 14/61, (23%); diabetes 7/61, (11.5%); heart disease 12/61, (19.7%); COPD 14/61, (23%) ASA I (n/N,%): 0/61, 0% ASA II (n/N,%): 8/61 (13.1%) ASA III (n/N,%): 53/61 (86.9%) ASA IV (n/N,%): 0/61, 0% Number of participants randomised: 61 Number of participants receiving treatment: 61 Number of participants analysed: 61 Dropout rate: 0/61, 0% **Inclusion criteria** Consecutive adults undergoing hip fracture surgery for isolated intertrochanteric fracture (AO 31A) treated with PFNA (XiaMen Double) Aged > 18 years Signed informed consent from the patient or legal representative **Exclusion criteria** Allergy or any contraindication for TXA Delayed admission beyond 24 h Pathological fracture or open fracture Multiple fractures or trauma Discontinuation of oral anticoagulants or aspirin in < 1 week History of acute thromboembolic event (DVT, PE, stroke) Patients at high risk of thrombosis Coagulopathy (INR > 1.4) Blood transfusion before surgery Creatinine clearance < 30 mL/min Congenital or acquired clotting disorders Pregnancy or breastfeeding > 1 current fracture **Tourniquet use:** not reported **Type of anaesthetic:** general or spinal anaesthesia was selected by anaesthetists without regional blockade **Type of surgery:** intertrochanteric fracture surgery: hip fracture surgery for isolated intertrochanteric fracture (AO 31A) treated with PFNA (XiaMen Double)	
Interventions	**Placebo arm** 2 doses of IV NS (100 mL) administered over 10 min, 1 dose just 10 min before incision by anaesthetists, and the 2nd 3 h later by nurses **TXA arm** 2 doses of 1 g IV TXA (100 mL: 1 g; Chongqing Lummy Pharmaceutical Co Ltd, Chongqing, China) administered over 10 min, 1 dose just 10 min before incision by anaesthetists, and the 2nd 3 h later by nurses	
Outcomes	**Primary outcome** Hidden blood loss **Secondary outcomes** Allogeneic erythrocyte transfusion rate during hospitalisation Composite of thromboembolic events including DVT up to 90 days	
Notes	**Sponsorship source:** non‐pharmaceutical (this study was funded by the Research Project of Mianyang Municipal Health and Family Planning Commission (201812) and General Incubation Project of The Third Hospital of Mianyang (201944)) **Country:** China **Setting:** single‐centre **Comments** All authors declared no conflict of interest. Complications reported up to 90 days, not available at 30 days: DVT, PE, MI, stroke, mortality **Authors name:** S Zhang (Shaoyun Zhang, Cong Xiao, and Wei Yu contributed equally to this work and should be considered as equal first authors) **Institution:** The Third Hospital of Mianyang, Sichuan Mental Health Center **Email:** zhangsyhm2068@163.com or Corresponding author: Yishan Jiang: jiangysh@126.com **Address:** Department of Orthopedics, The Third Hospital of Mianyang, Sichuan Mental Health Center, No. 190 The East Jiannan Road, Mianyang 621000, China **Native language of paper:** English **Reference type:** full text (1), trial registration (1) **Trial registration number:** ChiCTR1800018110 **Was it translated for this review:** no	
***Risk of bias***		
**Bias**	**Authors' judgement**	**Support for judgement**
Random sequence generation (selection bias)	Low risk	Quote: ''...using a computer‐ generated randomization list.'' Judgement comment: adequate method of sequence generation with computer–generated code
Allocation concealment (selection bias)	Unclear risk	Quote: ''A random allocation sequence concealed in opaque sealed envelopes was opened just before surgery.'' Judgement comment: envelopes not described as sequentially numbered
Blinding of participants and personnel (performance bias) subjective outcomes	Low risk	Quote: ''The patients, surgeons, data controller, and analyst were blinded to allocation until the final data analysis.'' Judgement comment: the participants, surgeons, data controller, and analyst were blinded to allocation until the final data analysis.
Blinding of participants and personnel (performance bias) objective outcomes	Low risk	Quote: ''The patients, surgeons, data controller, and analyst were blinded to allocation until the final data analysis.'' Judgement comment: the participants, surgeons, data controller, and analyst were blinded to allocation until the final data analysis.
Blinding of outcome assessment (detection bias) subjective outcomes	Low risk	Quote: not reported Judgement comment: ''The patients, surgeons, data controller, and analyst were blinded to allocation until the final data analysis.''
Blinding of outcome assessment (detection bias) objective outcomes	Low risk	Quote: not reported Judgement comment: ''The patients, surgeons, data controller, and analyst were blinded to allocation until the final data analysis.''
Incomplete outcome data (attrition bias) All outcomes	Low risk	Quote: not reported Judgement comment: all randomised (n = 122) included in analysis (n = 122); none lost to follow‐up, none excluded from analysis
Selective reporting (reporting bias)	Low risk	Quote: not reported Judgement comment: compared to trial registration ‐ all prespecified primary outcomes and adverse events reported
Other bias	Low risk	Quote: not reported Judgement comment: no other concerns such as baseline imbalance or early stopping. Funding sources listed, authors declare no conflicts of interest

**AO/OTA:** Arbeitsgemeinschaft für Osteosynthesefragen/Orthopaedic Trauma Association**; APTT:** activated partial thromboplastin time; **ASA:** American Society of Anesthesiologists; **COPD:** chronic obstructive pulmonary disease; **CPR:** cardiopulmonary resuscitation; **CR:** computed radiography; **CT:** computed tomography; **CVA:** cerebrovascular accident; **DVT:** deep vein thrombosis; **F:** female; **FFP:** fresh frozen plasma; **Hb:** haemoglobin; **ICU:** intensive care unit; **INR:** international normalisation ratio; **ITT:** intention to treat; **IV:** intravenous; **M:** male; **MI:** myocardial infarction; **MRI:** magnetic resonance imaging; **NS:** normal saline; **PE:** pulmonary embolism; **PFNA:** proximal femoral nail anti‐rotation; **PT:** prothrombin time; **PTT:** partial thromboplastin time; **PT‐INR:** prothrombin time international normalisation ratio; **n/N:** number of people experiencing the event/number of people in analysis; **RBC:** red blood cell; **RCT:** randomised controlled trial; **rFVIIa:** recombinant activated factor VII; **SD:** standard deviation; **TBI:** traumatic brain injury; **TXA:** tranexamic acid; **VTE:** venous thromboembolism

### Characteristics of excluded studies [ordered by study ID]

**Table:**

Study	Reason for exclusion
ACTRN 12613000323729	Ineligible patient population
ACTRN 12613001043729	Ineligible patient population
Ahmed 2010	Ineligible comparator
Alipour 2013	Ineligible patient population
Anonymous 2019 (various)	Ineligible study design
Antinolfi 2010	Ineligible patient population
Arslan 2018	Ineligible patient population
Barrachina 2016	Ineligible patient population
Baruah 2016	Author confirmed trial was not registered
Batibay 2018	Author confirmed trial was not registered
Benoni 2001	Ineligible patient population
Bidolegui 2014	Ineligible patient population
Borisov 2011	Ineligible patient population
Bradley 2019	Ineligible patient population
Camarasa 2006	Ineligible patient population
Cankaya 2017	Ineligible patient population
Cao 2015	Ineligible study design
Cao 2018	Ineligible patient population
Cao 2019	Ineligible patient population
Castro‐Menendez 2016	Ineligible patient population
Cerciello 2014	Ineligible patient population
Chen 2018	Inelegible patient population: participants had hip replacements due to osteoarthritis rather than trauma. Required full‐text translation to confirm exclusion.
ChiCTR 1800016634	Study withdrawn prior to starting
ChiCTR 1800019266	Retrospectively registered
ChiCTR 1900027435	Retrospectively registered
ChiCTR 2000032102	Retrospectively registered
ChiCTR 2000032836	Retrospective registration
ChiCTR 2000033135	Retrospective registration
ChiCTR 2000034882	Retrospective registration
ChiCTR‐IDR‐17010966	Retrospectively registered
ChiCTR‐TRC‐14004379	Retrospectively registered
Chin 2020	Ineligible patient population
Clave 2019	Ineligible patient population
Colwell 2007	Ineligible patient population
Cvetanovich 2018	Ineligible patient population
D'Ambrosio 1998	Ineligible patient population
Ekback 2000	Ineligible patient population
Fischer 2013	Ineligible patient population
Fleischmann 2011	Ineligible patient population
Flordal 1991	Ineligible patient population
Fraval 2017	Ineligible patient population
Fraval 2018	Ineligible patient population
Galué 2015	Ineligible comparator
Garcia‐Enguita 1998	Ineligible patient population
Gausden 2016	study withdrawn prior to starting
Gillespie 2015	Ineligible patient population
Gomez Barbero 2019	Ineligible patient population
Gulabi 2019	Ineligible patient population
Hourlier 2012	Author confirmed trial not registered
Huang 2021	Ineligible comparator
IRCT 201111198131N	Retrospectively registered
IRCT 2013100414302N	Retrospectively registered
IRCT 2016061328437N	Retrospectively registered
IRCT 2017050126328N	Retrospectively registered
IRCT 20180404039188N2	Retrospectively registered
IRCT 20180422039382N	Retrospectively registered
IRCT 20200114046133N1	Retrospective registration
IRCT 20211208053326N1	Retrospective registration
ISRCTN 02543733	Retrospectively registered
ISRCTN 55488814	Retrospectively registered
ISRCTN 58762744	Retrospectively registered
ISRCTN 59245192	Retrospectively registered
Ivie 2016	Ineligible patient population
Jans 2016	Ineligible patient population
Jaszczyk 2015	Ineligible patient population
Jordan 2016	Retrospectively registered
Jordan 2019	Retrospectively registered
Koea 2015	Ineligible patient population and ineligible comparator
Lack 2017	Retrospectively registered
Lei 2018	Ineligible patient population
Liu 2015	Ineligible comparator
Llau 1998	Ineligible patient population
Luo 2012	Ineligible comparator
Mukherjee 2016	Author confirmed trial not registered ‐ personal email to Dr Mukherjee
Na 2016	Ineligible patient population
Najafi 2014	Retrospectively registered
Narkbunnam 2021	Retrospective registration
NCT00375440	Study withdrawn prior to starting
NCT00658723	Ineligible patient population
NCT00824564	Ineligible comparator
NCT01199627	Ineligible patient population (required correspondence with trialists to confirm)
NCT01326403	Study withdrawn prior to starting
NCT01535781	Retrospectively registered
NCT01714336	Retrospectively registered
NCT01866943	Retrospectively registered
NCT02043132	Retrospectively registered
NCT02051686	Retrospectively registered
NCT02080494	retrospective trial registration
NCT02150720	Retrospectively registered
NCT02164565	Study withdrawn prior to starting
NCT02233101	Ineligible patient population
NCT02252497	Retrospectively registered
NCT02569658	Ineligible patient population
NCT02580227	Retrospectively registered
NCT02584725	Ineligible patient population
NCT02644473	Study withdrawn prior to starting
NCT02684851	Retrospectively registered
NCT02747615	Retrospectively registered
NCT02908516	Study withdrawn prior to starting
NCT02947529	Retrospectively registered
NCT03019198	Retrospectively registered
NCT03251469	Retrospectively registered
NCT03653429	Retrospectively registered
NCT03679481	Study withdrawn prior to starting
NCT03825939	Retrospectively registered
NCT04488367	Retrospectively registered
NCT04696224	Retrospectively reegistered
NCT04803591	Study withdrawn prior to starting ‐ withdrawn (not approved by Ethics Committee)
NCT04986813	Retrospectively registered
NCT05047133	Retrospectively registered
Nikolaou 2021	Retrospectively registered
North 2016	Ineligible patient population
Ozay 1995	Ineligible comparator
Petsatodis 2006	Ineligible patient population
Qiu 2019	Ineligible patient population
Rajesparan 2009	Ineligible comparator
Ruiz‐Moyano 1997	Ineligible comparator
Samama 2002	Ineligible patient population
Saravanan 2020	Retrospective registration
Schiavone 2018	Author confirmed trial not registered
Shodipo 2022	Author confirmed trial not registered
TCTR 20201224005	Retrospectively registered
TCTR 202102090010	Retrospective registration
TCTR 20220104001	Retrospective registration
Tengberg 2016	Retrospectively registered
Thipparampall 2017	Author confirmed trial not registered
Tulaja Prasad 2021	Ineligible patient population
Van Elst 2013	Retrospectively registered
Vara 2017	Ineligible patient population
Vles 2020	Ineligible patient population
Wang 2016	Ineligible patient population
Wang 2019	Ineligible patient population
Watts 2017	Retrospectively registered
Wei 2014	Ineligible patient population
Wendt 1982	Ineligible patient population
Xie 2016	Ineligible patient population
Yamasaki 2004	Ineligible patient population
Yee 2022	Retrospective registration
Yu 2020	Ineligible study design
Zhao 2016	Ineligible patient population
Zhou 2019	Author confirmed trial not registered
Zufferey 2010	Retrospectively registered

### Characteristics of studies awaiting classification [ordered by study ID]

**Table:** Akram 2021

Methods	RCT, parallel
Participants	**Inclusion criteria** Adults 18‐80 years Boyd and Griffin Type 1, 2 and 3 intertrochanteric fractures **Exclusion criteria** Bleeding diathesis Hb < 8 g/dL Open fractures
Interventions	**Intervention** 15 mg/kg of TXA at the time of induction of anaesthesia, repeated after 3 h **Comparator** IV placebo (NS), given by a resident who was not part of the surgical team
Outcomes	Fall in Hb Blood loss
Notes	No trial registration information. Authors emailed (24 May 2022)

**Table:** Chen 2019

Methods	RCT Parallel, 2‐arm
Participants	**Inclusion criteria** Aged ≥ 65 years Trochanteric fractures surgically treated by dynamic hip screw and proximal anti‐rotating intramedullary nail ASA scores of II or III **Exclusion criteria** Allergy to TXA or low‐molecular weight heparin Severe dysfunction of heart, lung, liver, kidney, or coagulation Provoked DVT or PE within 30 days or MI, CVA, or stent placement within 6 months Anticoagulant therapy such as antiplatelet drugs or warfarin before surgery Multiple fractures Blood transfusion before surgery
Interventions	**Intervention** 3 doses of 15 mg/kg IV TXA dissolved in 100 mL of saline. Each of the doses was administered over 10 min: the first dose was used within 10 min just before incision, the second continuously pumped throughout the entire surgery, and the third was used at 3 h after surgery (3‐dose regimen) **Comparison** 100 mL of saline solution administered following the same 3‐dose regimen (placebo group)
Outcomes	**Primary outcomes** Perioperative blood loss Proportion of patients receiving blood transfusion from the beginning of surgery to discharge
Notes	No trial registration information. Authors emailed ‐ no response

**Table:** ChiCTR 1800015265

Methods	RCT Parallel
Participants	**Inclusion criteria** Adults undergoing total hip arthroplasty **Exclusion criteria** Infection, anaemia, revision surgery, anaphylactic allergies to TXA
Interventions	**Intervention** Oral TXA **Comparator** IV TXA
Outcomes	**Primary outcomes** Blood loss Transfusion requirements TXA cost
Notes	Unable to assess whether participants, or a subgroup of participants, are having the operation after a hip fracture

**Table:** ChiCTR‐IPR‐17011260

Methods	RCT
Participants	**Inclusion criteria** Adults aged ≥ 65 years Intertrochanteric fracture Treated surgically with PFNA
Interventions	**Intervention** TXA IV (no comparator mentioned)
Outcomes	Total blood loss Intraoperative blood loss Hb concentration on first post‐operative day Postoperative Hb decreased maximum Rate of transfusion Rate of vascular events Changes in blood coagulation index Changes in fibrinolysis index Rate of wound infection Duration of surgery Length of hospital stay
Notes	As of 13 August 2021 there was no response to multiple emails sent to authors asking for an update on trial status. Unclear comparator (not mentioned)

**Table:** CTRI/2018/02/012030

Methods	Parallel 3‐arm RCT
Participants	**Inclusion criteria** Adults undergoing primary unilateral hip replacement **Exclusion criteria** Allergy to TXA Administered anticoagulants or antiplatelet drugs preoperatively Ischaemic heart disease Chronic renal failure History of hip surgery Thromboembolic episodes Rheumatoid arthritis
Interventions	**Intervention 1** The topical TXA group receives 2 g of TXA in a 10 mL solution. After the prosthesis is inserted, the entire operative field is thoroughly rinsed and dried meticulously. The TXA is applied by syringe‐spray to the following surfaces: the posterior capsule, the surrounding soft tissues, including the muscles and tendons, fatty and subcutaneous tissue, and the exposed surfaces of the femur and acetabulum. **Intervention 2** IV TXA at a dose of 10 mg/kg administered 30 min prior to skin incision **Comparator** Usual care
Outcomes	**Primary outcomes** Total blood loss Change in haematocrit levels at 2nd postoperative day Operation time
Notes	Unable to assess whether participants, or a subgroup of participants, are having the operation after a hip fracture.

**Table:** Drakos 2016

Methods	RCT
Participants	**Inclusion criteria** ≥ 65 years Intertrochanteric fracture **Exclusion criteria** Polytrauma Pathologic fracture Known history of malignancy Delayed surgery beyond 48 h Known allergy to TXA History of venous or arterial thromboembolic disease (DVT, PE, CVA) Hepatic failure Severe renal insufficiency Haematologic disorder (thrombogenic, haemorrhagic, hematopoietic disease) Coumadin anticoagulant medication Coagulopathy
Interventions	**Intervention** Local administration of TXA **Comparator** Control (no TXA)
Outcomes	Number of transfused packed RBC units Haematocrit Haemoglobin Platelet count
Notes	No trial registration information. Authors emailed ‐ no response

**Table:** Emara 2014

Methods	RCT
Participants	**Inclusion criteria** Age ranged from 50‐60 years Undergoing hemiarthroplasty surgeries for fractured hip joint within 48 h of trauma **Exclusion criteria** Allergy to TXA Acquired disturbances of colour vision Pre‑operative anaemia (haemoglobin < 11 gm% in female and haemoglobin < 12 gm% in male participants) Preoperative use of anticoagulant therapy i.e. oral anticoagulants, heparin within 5 days of surgery, fibrinolytic disorders requiring intraoperative antifibrinolytic treatment Coagulopathy i.e. preoperative platelets count < 150,000 mm3, INR > 1.4 and prolonged PT > 1.4 s A previous history of thromboembolic disease i.e. DVT, CVS and PE Significant comorbidities Severe ischaemic heart disease, New York Heart Association Class III and IV Previous MI Severe pulmonary disease Plasma creatinine > 115 mmol/L in men and > 100 μmol/L in women Hepatic failure Occurrence intraoperative surgical/medical/anaesthetic complications i.e. MI or neurovascular injury Patients who need massive blood transfusion Postoperative bleeding of surgical causes
Interventions	**Intervention 1** Intravenous TXA **Intervention 2** Topical TXA **Comparator** Control group (placebo saline)
Outcomes	Post‐operative bleeding Haemoglobin concentration, haematocrit, platelets and coagulation profile (prothrombin time, activated partial thromboplastin time and INR) Thromboelastography Incidence of DVT, PE and CVA
Notes	No trial registration information. Authors emailed ‐ no response

**Table:** Kazemi 2010

Methods	RCT
Participants	**Inclusion criteria** Candidates for cementless total hip arthroplasty **Exclusion criteria** Patients with previous hip surgery Drug sensitivity Anemia (haemoglobin 11.5 for women and 12.5 for men) Congenital or acquired haemostatic disease Disturbed coagulation and platelet count Hepatic or renal failure Pregnancy History of DVT, embolism and atherosclerotic vascular disease
Interventions	**Intervention** IV TXA **Comparator** Placebo
Outcomes	Haemoglobin levels Mean blood loss Need for transfusion Haematocrit Length of hospital stay Thromboembolic events
Notes	Unable to assess whether participants, or a subgroup of participants, are having the operation after a hip fracture

**Table:** Li 2021

Methods	RCT, parallel, 3‐arm trial
Participants	**Inclusion criteria** Diagnosed intertrochanteric fractures Planned closed reduction and internal fixation with Gamma‐3 intramedullary nails (Chuangsheng Medical Devices Co Ltd) Age ≥ 60 years old Patients and their families gave informed consent **Exclusion criteria** Abnormal coagulation function or using anticoagulant drugs Hb < 90 g/L Combined peripheral nerve and vascular disease Malignant tumour Affected limb had a history of infection
Interventions	**Intervention 1** 100 mL of NS containing TXA (15 mg/kg) was infused intravenously 30 min before surgery; 50 mL of NS was injected into the medullary cavity after the proximal femur was slotted and before the intramedullary nail was implanted during the operation **Intervention 2** 100 mL of NS was infused IV 30 min before surgery; 50 mL of NS containing 1 g TXA was injected into the medullary cavity after the proximal femur was slotted and before the intramedullary nail was implanted. **Comparator** NS containing TXA was given before and during the operation
Outcomes	Blood loss Complications (PE, incision infection, DVT) Operation time Blood transfusion
Notes	No trial registration information. Authors emailed (24 May 2022)

**Table:** Lin 2021

Methods	RCT
Participants	**Inclusion criteria** Patients with intertrochanteric fracture
Interventions	**Intervention** The observation group was given TXA 0.5 g dissolved in 20 mL normal saline injected into femoral bone marrow cavity for local treatment on the basis of the control group. **Comparator** The control group was given TXA 20 min before operation,and 15 mg/kg diluted in 250 mL sodium chloride injection, IV drip
Outcomes	Blood loss Operation time Postoperative hospital stay Haematocrit Hb, D‐dimer and fibrinogen levels Incidence of thrombotic complications
Notes	No trial information. Authors emailed (24 May 2022) Translation needed

**Table:** Liu 2022

Methods	RCT (parallel), double‐blind
Participants	**Inclusion criteria** Elderly people (> 65 years) Hip fracture (including femoral neck fracture and intertrochanteric fracture)
Interventions	**Intervention** 1.5 g of TXA IV every 12 h from post‐admission day 1 (PAD1) to the day before surgery **Comparator** 100 mL NS Both groups were treated with 1.5 g of TXA every 12 h from postoperative days 1‐3
Outcomes	**Primary outcomes** Hidden blood loss Haemoglobin decrease Allogeneic blood transfusion rate **Secondary outcomes** Levels of inflammatory factors (such as C reactive protein) Coagulation and fibrinolysis parameters (such as D‐dimer) Injury time Length of stay Hospitalisation expenses
Notes	No trial registration information. Authors emailed

**Table:** Luo 2018

Methods	RCT
Participants	**Inclusion criteria** Elderly female patients with femoral neck fracture **Exclusion criteria** DVT prior to operation Other medical conditions that required aspirin or other anti‐platelet agents Postoperative coagulation disorder Using low‐molecular heparin Femoral neck fracture plus fracture at other places Dementia and other conditions that can interfere with compliance Fracture recurred during operation Transferring to another department due to post‐operation complication
Interventions	**Intervention 1** IV TXA **Intervention 2** Local medication **Intervention 3** Combined IV and local medication **Comparator** Placebo (saline)
Outcomes	Post‐operative drainage volume Haemoglobin level Total blood loss Haematocrit prior to operation
Notes	No trial registration information. Authors emailed ‐ no response

**Table:** Moghaddam 2009

Methods	RCT
Participants	**Inclusion criteria** Patients aged 20‐50 years with femoral fractures **Exclusion criteria** Anaemia Underlying diseases such as renal disease History of myocardial ischemia Hypertension History of cerebral ischemia History of thromboembolism
Interventions	**Intervention** IV TXA **Comparator** Placebo (saline)
Outcomes	Bleeding volume Drug side effects
Notes	No trial registration information. Authors emailed ‐ no response

**Table:** Mohib 2015

Methods	RCT
Participants	**Inclusion criteria** Patients age 50‐90 years diagnosed with Intertrochanteric fracture on X‐ray imaging **Exclusion criteria** Multiple fractures on X‐ray Rheumatoid arthritis Ischaemic heart disease Pregnant or lactating women Known coagulation disturbances Use of warfarin or other anticoagulants Allergy to TXA
Interventions	**Intervention** 2 doses of 10 mg/kg body weight of TXA just before surgery and 3 h later IV **Comparator** 2 es of 10 mg/kg body weight of NS at similar intervals
Outcomes	Haemoglobin Need for blood transfusion
Notes	No trial registration information. Authors emailed ‐ no response

**Table:** NCT01683955

Methods	RCT
Participants	**Inclusion criteria** All adult patients > age 18 years Primary unilateral total hip arthroplasty at Henry Ford Hospital (Detroit, Michigan, USA) and Henry Ford West Bloomfield Hospital (West Bloomfield, Michigan, USA) **Exclusion criteria** Patient history of venous thromboembolic disease or coagulopathy Use of anticoagulant medications within 7 days of surgery History of arterial embolic disease History of Class III or IV heart failure Renal failure Intraoperative cardiovascular, pulmonary, orthopaedic, or anaesthetic complication (MI, intraoperative fracture, vasopressor support, emergent intubation)
Interventions	**Intervention** Topical TXA **Comparator** Placebo (saline)
Outcomes	**Primary outcome** Postoperative blood loss **Secondary outcome** Postoperative transfusion rate
Notes	Unable to assess whether participants, or a subgroup of participants, are having the operation after a hip fracture

**Table:** NCT02094066

Methods	RCT
Participants	**Inclusion criteria** ASA 2‐3 Aged 18‐75 years Total hip arthroplasty surgery Regional anaesthesia **Exclusion criteria** Allergies to drug Liver and kidney failure Ischaemic heart disease Coagulopathy
Interventions	**Intervention** IV TXA **Comparator** Physiological serum
Outcomes	**Primary outcome** Haemorrhage **Secondary outcome** Erythrocyte transfusion
Notes	Unable to assess whether participants, or a subgroup of participants, are having the operation after a hip fracture.

**Table:** NCT02438566

Methods	RCT
Participants	**Inclusion criteria** Undergoing hip or bilateral knee replacement surgery Healthy enough to undergo joint replacement surgery YAble to understand and sign an informed consent ≥ 18 years of age **Exclusion criteria** < 18 years of age Undergoing revision hip or revision bilateral knee replacement surgery Allergic to the medication Haemodialysis Active coronary artery disease and vascular stents Ever had a blood clot (DVT, PE) Ever had a cerebral or subarachnoid haemorrhage (brain bleeding), or stroke (CVA or transient ischaemic attack) On oestrogen‐containing medication (hormone replacement therapy or oral contraceptive) within 7 days of surgery
Interventions	**Intervention** Oral TXA **Comparator** IV TXA
Outcomes	**Primary outcome ** Lower number of units of blood required for transfusion **Secondary outcome ** Lower incidences of patients requiring blood transfusion Lower blood loss in patients Length of stay
Notes	Unable to assess whether participants, or a subgroup of participants, are having the operation after a hip fracture

**Table:** NCT02738073

Methods	RCT
Participants	**Inclusion criteria** Patients admitted to Bryn Mawr Hospital with fracture of the femoral neck, intertrochanteric region, or subtrochanteric region of the femur **Exclusion criteria** Age < 18 Allergy to TXA Known current or history of VTE History of known coagulopathy or bleeding disorder Current subarachnoid haemorrhage Previous history of seizures Current use of oestrogen/progesterone therapy Renal failure defined as creatinine clearance < 30 mL/min Multiple fractures Pregnant or breastfeeding women Planned nonoperative management of the fracture
Interventions	**Intervention** IV TXA **Comparator** Placebo (saline)
Outcomes	**Primary outcome** Blood loss during the perioperative period
Notes	Unable to clarify whether prospectively registered

**Table:** NCT03157401

Methods	RCT
Participants	**Inclusion criteria** Femoral head necrosis or femoral neck fracture patients undergoing the first unilateral total hip arthroplasty Bilateral hips with indications for total hip arthroplasty in patients with femoral head necrosis, but after arthroplasty on one side, the arthroplasty on the other side will be conducted when choosing a good time and physical condition allows Average age: 62.52 years Sex ratio male to female: 11:19 Signed informed consent **Exclusion criteria** Coagulation disorders and anaemia History of infection on the affected extremity History of vascular embolisation and long‐term oral anticoagulant drugs Contraindications for TXA or anticoagulant drugs
Interventions	**Intervention 1** IV infusion of TXA **Intervention 2** Intra‐articular injection of TXA **Comparator** Saline
Outcomes	**Primary outcome** Hidden blood loss **Secondary outcome** Dominant blood loss
Notes	Includes both femoral head necrosis or femoral neck fracture patients undergoing the first unilateral total hip arthroplasty. However, unclear whether separate subgroups will be reported

**Table:** NCT03822793

Methods	RCT
Participants	**Inclusion criteria** Patient requiring primary hip arthroplasty (< 3 months) Consent of the patient or a family member or the support person **Exclusion criteria** Contraindication to TXA Contraindication to apixaban Pregnancy Patient receiving a curative anticoagulating treatment in the preoperative period Bilateral or previous hip arthroplasty Haemorrhagic surgery < 2 weeks old
Interventions	**Intervention 1** 500 mg IV TXA **Intervention 2** 1000 mg IV TXA **Intervention 3** 1500 mg IV TXA **Intervention 4** 3000 mg IV TXA **Comparator** Placebo
Outcomes	**Primary outcome** Haemoglobin decrease in the perioperative period **Secondary outcome ** Evolution of the concentration of TXA Allogeneic red blood cell transfusion Severe anaemia Incidence of symptomatic thrombotic events and death Occurrence of a seizure
Notes	Unable to assess whether participants, or a subgroup of participants, are having the operation after a hip fracture

**Table:** NCT03897621

Methods	RCT
Participants	**Inclusion criteria** The study population will include total of 200 adults (age range of 18‐85 years) ASA 1‐3. Patients undergoing unilateral, primary, total hip arthroplasty **Exclusion criteria** Exclusion criteria include patient's refusal, patients with history of significant coagulopathy or on anticoagulation therapy. Female patients who are pregnant or nursing will be excluded. In addition, patients with anaemia (Hb < 8 g/dL) or who received blood transfusion within one week before surgery will be excluded. Patient receiving subcutaneous heparin on the same day prior to surgery will be also excluded.
Interventions	**Intervention** IV TXA **Comparator** Placebo (saline)
Outcomes	**Primary outcome ** Fibrinolysis **Secondary outcome** Blood loss Blood transfusion Pre‐ and postoperative haemoglobin level Wound infection Haematoma Thrombotic events
Notes	Unable to assess whether participants, or a subgroup of participants, are having the operation after a hip fracture

**Table:** NCT04089865

Methods	RCT
Participants	**Inclusion criteria** Patients undergoing total hip arthroplasty through a posterior approach Patients undergoing total knee arthroplasty Patients between 18‐80 years of age **Exclusion criteria** Patients with > 80 years of age Patients with a BMI > 40 Patients undergoing general anaesthesia Patients with a history of major ipsilateral joint surgery Patients on preoperative anticoagulation or anti‐platelet drugs (other than aspirin) Patients with a history of bleeding disorders Patients with platelets < 100/nL Patients with new‐onset/active atrial fibrillation Patients with a history of MI in the past year Patients with a history of a stroke in the past year
Interventions	**Intervention** Oral TXA **Comparator** IV TXA
Outcomes	**Primary outcome** Calculated blood loss **Secondary outcome** Transfusion during hospital stay Time to discharge from physical therapy Length of stay Hospital length of stay (in hours)
Notes	Unable to assess whether participants, or a subgroup of participants, are having the operation after a hip fracture

**Table:** NCT04187014

Methods	RCT
Participants	**Inclusion criteria** Age > 18 years Total replacement of the primary hip due to: 1)primary coxarthrosis, 2) avascular hip necrosis, 3) transcervical fracture Unilateral procedure Press‐fit prosthesis Without the use of cement for the placement of the prosthesis Desire to participate voluntarily in the study and signature of informed consent Pre‐operative assessment with result between ASA I, ASA II or ASA III performed and annexed in the clinical file either by the Department of Internal Medicine, Cardiology or Anesthesiology Possibility for oral administration of the drug **Exclusion criteria** History of thrombotic or embolic event in the last 6 months Clinical history of coagulopathy Previous surgeries in the hip to intervene Patients who have received aspirin, platelet or coumarinic antiplatelet agents in the week prior to surgery or NSAIDs two days prior to surgery History of MI, arteriopathy or unstable angina in the 12 months prior to surgery Those patients whose preoperative assessment corresponds to an ASA IV or the procedure is contraindicated in its preoperative assessment Revision hip replacement Tumoral hip replacement Bilateral hip replacement Cognitive deficit Patients who meet the inclusion criteria but do not wish to participate in the study Patients with a diagnosis of terminal chronic kidney disease or with a serum creatinine > 1.47 mg/dL in the preoperative laboratories Patients with inability to ingest the drug orally Patients who are pregnant or breastfeeding or who are taking oral contraceptives Seizure history Hypersensitivity to the active substance or to any of the excipients
Interventions	**Intervention** Oral TXA **Comparator** Oral aminocaproic acid
Outcomes	**Primary outcome ** Total blood loss (TBL) External blood loss (EBL) Hidden blood loss (HBL) **Secondary outcome ** Change in haematocrit level Drainage quantification Therapeutic effect on VAS Change in haemoglobin level Rate of complications Rate of transfusion Rate of intraoperative blood loss
Notes	Unable to assess whether participants, or a subgroup of participants, are having the operation after a hip fracture

**Table:** Notarfrancesco 2015

Methods	RCT
Participants	**Inclusion criteria** Not reported **Exclusion criteria** Not reported
Interventions	**Intervention** Topical TXA and IV TXA **Comparator** Control
Outcomes	**Primary outcome** Blood transfusion rate Postoperative bacterial infection rate **Secondary outcome** Visible blood loss Haemoglobin values
Notes	Unable to assess whether participants, or a subgroup of participants, are having the operation after a hip fracture

**Table:** Sahni 2021

Methods	RCT
Participants	**Inclusion criteria** Hip trauma Undergoing hip surgery within 5 days of injury Aged 50–75 years **Exclusion criteria** Multiple fractures Pregnant or breastfeeding Any contraindication to TXA such as previous seizures, previous arterial, or venous thrombosis Anticoagulation therapy that could not be stopped
Interventions	**Intervention ** IV transfusion of TXA 15 mg/kg (2 doses) **Comparator ** IV placebo (2 doses)
Outcomes	Preoperative Hb, values of Hb at the day of surgery, and values at postoperative day 7 Total number of blood transfusions Intraoperative and postoperative blood loss Postoperative bacterial infections including superficial and deep wound infections, septic arthritis, and any other major infection up to 6 weeks following surgery Major incidences of postoperative bleeding Length of stay in hospital Any thromboembolic events Mortality up to 6 months
Notes	No trial registration information. Authors emailed (24 May 2022)

**Table:** Singh 2020

Methods	RCT, 2‐arm, double‐blind, parallel
Participants	**Inclusion criteria** Age 18‐70 years Primary total hip replacement Primary knee replacement Major spine surgeries ‐ decompression with instrumentation **Exclusion criteria** Revision arthroplasty Revision spine surgery Comorbid medical conditions Active infection
Interventions	**Intervention** IV TXA 500 mg just before skin incision, and 500 mg slow infusion started just before surgery **Comparator** Saline placebo
Outcomes	Total blood loss Fall in Hb levels
Notes	Study period: 1 July 2018‐30 June 2019 Contacted authors 21 June 2022. Require trial registration information No trial registration information. Authors emailed

**Table:** Spitler 2019

Methods	RCT
Participants	**Inclusion criteria** Adult patients with isolated fractures of the pelvic ring, acetabulum, or femur requiring an open approach for reduction and fixation with an expected blood loss (EBL) of.300 mL. Unanimous agreement of the attending trauma surgeons involved in the study was required that anticipated EBL would exceed 300 mL based on the planned open surgical approach for fracture reduction and fixation. **Exclusion criteria** Pregnancy Open fracture Renal insufficiency Known hypercoagulable state (e.g. history of VTE and factor V Leiden) History of anticoagulation drug use (e.g. clopidogrel, warfarin, and low‐molecular weight heparin; aspirin was not an exclusion) Patients who had an associated traumatic injury that was a contraindication to immediate VTE prophylaxis (intracranial haemorrhage, spinal column fracture, and high‐grade intra‐abdominal solid organ injury) Any surgery before orthopaedic intervention (e.g. exploratory laparotomy and thoracotomy) All who had other major injuries that would have required a major surgery after orthopaedic intervention
Interventions	**Intervention** IV TXA **Comparator** Control
Outcomes	**Primary outcome** Total blood loss Change in preoperative to postoperative Hb/haematocrit values Units of allogeneic blood transfused
Notes	No trial registration information. Authors emailed ‐ no response

**Table:** Taheriazam 2015

Methods	RCT
Participants	**Inclusion criteria** Not reported **Exclusion criteria** Not reported
Interventions	**Intervention** IV TXA **Comparator** Local administration of TXA
Outcomes	Need for blood transfusion Haemoglobin drop
Notes	No trial registration information. Authors emailed ‐ no response

**Table:** Taheriazam 2016

Methods	RCT
Participants	**Inclusion criteria** Not reported **Exclusion criteria** Not reported
Interventions	**Intervention** IV TXA **Comparator** Local administration of TXA
Outcomes	Need for blood transfusion Haemoglobin drop
Notes	No trial registration information. Authors emailed ‐ no response

**Table:** Tian 2018

Methods	RCT
Participants	**Inclusion criteria** Intertrochanteric fracture patients > 65 years of age and generally in good condition with no severe systemic disease Platelet count, PT, PTT, INR within normal ranges **Exclusion criteria** Pathological fracture Allergy to TXA Serious cardiac or respiratory disease Congenital or acquired coagulopathy History of thromboembolic disease such as cerebral infarction, pulmonary embolism, MI, DVT Recent thrombophilia Preoperative hepatic or renal dysfunction (male creatinine level > 115 mmol/L, female creatinine level > 100 mmol/L) Diabetic
Interventions	**Intervention** IV TXA **Comparator** Control
Outcomes	Volume of intraoperative blood loss Postoperative drainage Need for postoperative blood transfusion Transfusion volume
Notes	No trial registration information. Authors emailed ‐ no response

**Table:** Vijay 2013

Methods	RCT
Participants	**Inclusion criteria** ASA grade I/II patients 18‐80 years Weighing 40‐100 kg Surgery for femoral fracture like open reduction internal fixation (ORIF), hemiarthroplasty, total hip replacement (THR) **Exclusion criteria** Patients with chronic disease like rheumatoid arthritis Ischaemic heart disease Malignancy History of any previous thromboembolic episodes Haemoglobin < 8 g/dL
Interventions	**Intervention** IV TXA **Comparator** Control
Outcomes	Postoperative bleeding Percentage fall of haemoglobin Transfusions Complications
Notes	No trial registration information. Authors emailed ‐ no response

**Table:** Wang 2021

Methods	RCT, 3‐arm trial
Participants	**Inclusion criteria** PFNA intramedullary nails for the treatment of intertrochanteric fractures No abnormal preoperative coagulation function No obvious abnormal liver and kidney function before surgery Preoperative colour Doppler ultrasonography examination of both lower extremities showed no DVT or intermuscular vein thrombosis **Exclusion criteria** Recent anticoagulant users Thrombotic events in the past 1 year Open fractures or other fractures and trauma Severe anaemia before surgery Acute or chronic inflammatory infection before surgery
Interventions	**Intervention 1** 33 participants given IV TXA 1.0 g half an hour before operation **Intervention 2** 35 participants given the same medicine as the single‐dose group before the operation, and repeated IV drip of TXA 1.0 g at 3 and 6 h after the operation **Comparator** 32 participants given an equal volume of physiological saline half an hour before the operation
Outcomes	Operation time Intraoperative blood loss Postoperative drainage volume Perioperative Hb Haematocrit C‐reactive protein and interleukin‐6 Colour Doppler ultrasound examination of lower extremity deep vein was conducted; and the total blood loss, hidden blood loss and thrombosis rate were calculated before and 7 days after operation
Notes	No trial registration information Translation requested

**Table:** Wu 2016

Methods	RCT
Participants	**Inclusion criteria** Patients were included in the study if they were to undergoing revision total hip arthroplasty surgery **Exclusion criteria** Patients with a diagnosis other than revision total hip arthroplasty Patients on the presence of current infection and anticoagulation therapy Patients with history of thrombosis disease, and any kind of cancer
Interventions	**Intervention** Combined IV and topical TXA group (combined group) **Comparator** IV TXA alone group
Outcomes	**Primary outcome** Transfusion rate DVT or/and PE **Secondary outcome** Maximum Hb drop Total blood loss Drainage volume Length of hospital stays Other complications
Notes	No trial registration information. Authors emailed ‐ no response

**Table:** Yang 2020

Methods	RCT, parallel, participant‐blind
Participants	**Inclusion criteria** Age > 20 years; Displaced 3 and 4 part proximal humerus fractures Operation within 14 days of injury
Interventions	**Intervention** 15 min before the skin incision, 15 mg/kg body weight of TXA was injected IV **Comparator** 15 min before the skin incision, 15 mg/kg body weight of 0.9% sodium chloride solution was injected IV
Outcomes	Total blood loss Blood test results Blood transfusion rate Wound complications
Notes	No trial registration information. Authors emailed for detail (26 May 2022)

**Table:** Zhang 2019

Methods	RCT
Participants	**Inclusion criteria** > 60 years old with no severe systemic disease Normal platelet count, PT, PTT, and INR Low‐energy trauma Availability of complete medical records in the perioperative period **Exclusion criteria** Allergy to aminocaproic acid History of recent or ongoing thromboembolic event (PE or DVT) History of recent anticoagulation therapy History of subarachnoid bleeding, malignancy, pathological fracture, or prior surgery on the injured hip Disseminated intravascular coagulation or hepatic/renal diseases with impaired coagulation function ASA IV
Interventions	**Intervention** Aminocaproic acid **Comparator** Placebo (saline)
Outcomes	Haemoglobin level Haematocrit (Hct) Perioperative blood loss Postoperative drainage RBC transfusion rate and volume Complications, including surgical site infection, DVT, PE, haematoma, pneumonia, and renal failure
Notes	No trial registration information. Authors emailed ‐ no response

**Table:** Zhang 2020b

Methods	RCT, 3‐arms
Participants	**Inclusion criteria** Intertrochanteric fractures diagnosed by preoperative X‐ray films Age ≥ 65 years old No serious complications, cardiovascular and cerebrovascular diseases, or bleeding diseases Preoperative coagulation function and platelet count were normal, and Hb ≥ 70 g/L Fracture could be closed and reduced Preoperative ultrasound examination of lower extremity blood vessels showed no DVT Voluntary participation in clinical trials and signed informed consent **Exclusion criteria** Pathological fractures Complicated infection and immune system diseases Coagulation dysfunction or using anticoagulant drugs Complicated with multiple fractures
Interventions	**Intervention 1** The IV application group only received preoperative IV drip of TXA **Intervention 2** IV combined topical application group, preoperative IV infusion of TXA combined with intraoperative topical application of "TXA cocktail". **Comparator** The blank control group did not use TXA
Outcomes	Perioperative blood loss VAS scores at 12, 24, 48 h after surgery Inflammation indicators Post‐op complications
Notes	Translation needed No trial registration information. Authors emailed

**Table:** Zheng 2020

Methods	RCT
Participants	**Inclusion criteria** Diagnosed with intertrochanteric fracture Met the criteria for proximal femur anti‐rotation fixation operation. The criteria were set by senior surgeons Normal pre‐op baseline Hb, coagulation, liver and kidney function Consent for the study **Exclusion criteria** History of severe cardiovascular disease, thromboembolism or coagulopathy Pre‐op Hb < 90 g/L Abnormal kidney and liver function Pre‐op usage of anticoagulant agents Pre‐op DVT Allergic to TXA Pathological fracture Malignancy
Interventions	**Intervention 1** IV TXA (20 mg/kg in 100 mL 0.9% sodium chloride) 30 min prior operation. During operation, TXA 1 g in 20 mL 0.9% sodium chloride was injected post‐proximal femur intracavity **Intervention 2** IV TXA 30 min prior operation. IV TXA (20 mg/kg in 100 mL 0.9% sodium chloride) **Comparator** Intracavity dose of TXA during operation. TXA (20 mg/kg in 100 mL 0.9% sodium chloride)
Outcomes	**Primary outcomes** Total blood loss Hidden blood loss Blood transfusion rate Incidence of DVT
Notes	No trial registration information. Authors emailed (27 October 2021)

**ASA:** American Society of Anesthesiologists; **CVA:** cerebrovascular accident; **DVT:** deep vein thrombosis; **Hb:** haemoglobin; **INR:** international normalisation ratio; **IV:** intravenous; **MI:** myocardial infarction; **NS:** normal saline; **NSAID:** nonsteroidal anti‐inflammatory drug; **PE:** pulmonary embolism; **PFNA:** proximal femoral nail anti‐rotation; **PT:** prothrombin time; **PTT:** partial thromboplastin time; **RBC:** red blood cell; **RCT:** randomised control trial; **TXA:** tranexamic acid; **VAS:** visual analogue scale

### Characteristics of ongoing studies [ordered by study ID]

**Table:** ACTRN 12617000391370

Study name	Trial acronym ROTANOF
Methods	RCT, parallel Allocation concealment is done with the help of central randomisation done by computer. Methods used to generate the sequence in which participants will be randomised (sequence generation) Simple randomisation using a randomisation table created by computer software (i.e. computerised sequence generation) **Blinded (masking used):** the people administering the treatment/s, and the people assessing the outcomes
Participants	**Key inclusion criteria** Patients with intra‐capsular neck‐of‐femur fractures undergoing hemiarthroplasty (cemented or uncemented) or total hip arthroplasty (cemented, hybrid or uncemented) within 48 h from the time of injury. Minimum age: 18 years **Key exclusion criteria** Neck‐of‐femur fractures requiring fixation by other methods e.g. by cannulated screws, dynamic hip screw or intra‐medullary nail device Patients presenting 48 h from the time of injury. This includes patients transferred to Nepean hospital from other hospitals Contra‐indication to the administration of TXA Previous history of thrombosis Active thromboembolic disease (DVT, PE and cerebral thrombosis) Other contraindication to the use of TXA Patients with acquired disturbances of colour vision Patients with subarachnoid haemorrhage Previous history of seizure Creatinine clearance < 30 mL/min Hypersensitivity to TXA Patients who are unable to provide informed consent
Interventions	**Intervention group:** IV TXA in 3 doses (15 mg/kg). First dose will be administered at the time of induction and remaining 2 at 8 h and 16 h post‐op **Control group:** no TXA or any blood loss medications
Outcomes	**Primary outcome** (**1**) Incidence of acute postoperative blood transfusion Time point (1) within 7 days of administration of TXA **Secondary outcome** (**1**) Incidence of acute post‐operative DVT. which will be assessed with a bilateral lower limb doppler ultrasound Time point (1) Day 7 post‐administration of TXA **Secondary outcome** (**2**) To assess whether the administration of TXA in a 3‐dose IV protocol leads to a reduction in post‐operative drop in Hb in the study population Time point (2) Hb check on post‐op days 1, 3 and 5
Starting date	**Date of first participant enrolment: 21 March 2017**
Contact information	
Notes	Universal Trial Number (UTN) U1111‐1189‐6122 Author replied 30.5.22 ‐ saying that trial has completed, manuscript written and submitted for publication. The author will send us the manuscript once it has been accepted. Country: Australia

**Table:** ACTRN 12620001059954

Study name	Efficacy of perioperative tranexamic acid in patients undergoing trochanteric hip fracture surgery: a randomized placebo controlled trial
Methods	RCT (parallel) Procedure for enrolling a participant and allocating the treatment (allocation concealment procedures): sealed opaque envelopes Methods used to generate the sequence in which participants will be randomised (sequence generation): permuted block randomisation **Blinded (masking used)**: the people receiving the treatment/s, and the people administering the treatment/s
Participants	Sample size target: 184 **Key inclusion criteria** Patients of either gender ≥ 18 years Trochanteric fracture types AO 31‐A1, A2 Received within 1 week after sustaining the fracture (ASA) scores of 1 and 2 **Key exclusion criteria** Pre operative Hb < 10 g/dL Allergy to TXA Severe dysfunction of heart, lung, liver, kidney, or coagulation Provoked DVT or PE within 30 days or MI, CVA, or stent placement within 6 months Anticoagulant therapy such as antiplatelet drugs or warfarin before surgery Multiple fractures Pathological fractures Open fractures Periprosthetic fractures Pregnancy
Interventions	**Brief Name: Tranexamic Acid (TXA) usage in hip fracture surgery** 1 g IV TXA mixed in 100 mL of saline, bolused at the time of surgical incision in operation theatre to participants with dynamic hip screw fixation for intertrochanteric fractures.It will be administered by anaesthesist.Those assigned to the placebo group will receive an equivalent volume bolus of saline at the time of surgical incision. Peri operatively the transfusion trigger will be Hb concentration equal to 9 g/dL for all participants.When these triggers are met whole blood will be transfused. Only for participants at risk (acute coronary syndrome, severe left ventricular dysfunction, or chronic respiratory failure), if hypotension could not be corrected despite adequate volume replacement during surgery and in case of syncope, transient ischaemic attack, stroke, acute respiratory failure, or acute coronary syndrome after surgery the transfusion trigger will be Hb concentration of 10 g/dL. During surgery, blood losses will be replaced with Ringer's lactate in a 3:1 ratio, with 6% hydroxyethyl starch 130/0.4 (Voluven, Fresenius Kabi, Bad Homburg, Germany) in a 1:1 ratio, or both until haemoglobin concentration fell bellow the transfusion trigger point. Thereafter, participants will receive 1 unit of allogeneic packed red cell hourly at a time until haemoglobin concentration raised above the transfusion trigger. Postoperative fluid therapy will be standardised for the first 12 hours. Each participant received 15 mL/kg of rehydration fluid (Na 40 mmol/L, K 20 mmol/L, glucose 250 mmol/). **Comparator**/**control treatment** Participants in this group will undergo the same treatment as those in the intervention group, but instead of receiving TXA at surgical incision, they will receive NS. **Control group** Placebo
Outcomes	**Primary outcome** (**1**) Rate of blood transfusion from the time of surgery until discharge at 72 h after surgery and will be checked from patient record Time point (1) baseline 24 h before surgery 6 h postoperatively 24 h postoperatively 48 h post operatively and 72 h post operatively **Secondary outcome** (**1**) Symptomatic DVT will be assessed with doppler ultrasound Time point (1) 2 weeks, 6 weeks, 3 months and 6 months after surgery **Secondary outcome** (**2**) PE will be assessed with contrast CT of chest Time point (2) 2 weeks, 6 weeks, 3 months and 6 months after surgery **Secondary outcome** (**3**) Wound infection will be assessed by inspecting the incision site for redness, tenderness and discharge. Also laboratory tests, namely complete blood count (BC), erythrocyte sedimentation rate (ESR) and C reactive proteins (CRP) will be done to note any infection Timepoint (3) 2 weeks, 6 weeks, 3 months and 6 months after surgery **Secondary outcome** (**4**) Death of any participant during the follow‐up period if the participant did not attend the follow‐up date and confirmed with telephone or email Time point (4) 2 weeks, 6 weeks, 3 months and 6 months after surgery
Starting date	Date of first participant enrolment: 21 January 2021
Contact information	Sponsor: self‐funded (individual) **Principal investigator** Name: Dr Faaiz Ali Shah Address Assistant Professor Orthopaedics & Traumatology Lady Reading Hospital Peshawar Pakistan Street Khyber Bazar Peshawar Province Khyber Pakhtunkhwa City Peshawar Postal code 25000. Pakistan Phone +923349125394 Email faaizalishah@yahoo.com
Notes	Universal Trial Number (UTN) U1111‐1246‐0037 Countries: Australia and Pakistan

**Table:** ChiCTR 1800014309

Study name	
Methods	RCT
Participants	**Inclusion criteria** Aged 60 years Intertrochanteric fracture treated with PFNA **Exclusion criteria** DVT before operation Preoperative Hb < 8 g/dL Pathological fracture Multiple fractures, with the other fracture(s) also needing surgical treatment Allergy to TXA Contraindication for anticoagulation therapy Duration from injury to operation > 3 weeks
Interventions	**TXA (topical)** TXA 1 g is injected into the proximal femoral medullary cavity; n = 50 **Placebo (saline)** 20 mL of saline is injected into the proximal femoral medullary cavity, n = 50
Outcomes	Hidden blood loss; total perioperative blood loss; postoperative transfusion rate
Starting date	Study execute time：from 10 January 2018 to 01 January 2020
Contact information	Applicant: Xiangping Luo Applicant telephone: +86 18163885070 luoxiangping8@sina.com
Notes	Accessed 7 June 2022 (LJG). Date of Last Refreshed on 01 May 2018

**Table:** ChiCTR 1800015809

Study name	
Methods	RCT
Participants	Patients undergoing total hip arthroplasty for femoral neck fractures Platelet and coagulation functions were normal before operation There was no abnormality in venous colour ultrasound of both lower extremities before operation Voluntary participation in clinical trials and signed informed consent, Patients with good compliance. **Exclusion criteria** History of VTE, PE, cerebral infarction, coronary heart disease Coagulation disorder Using anticoagulant drugs Stopping oral NSAIDs for < 1 week Allergies Severe hepatic and renal insufficiency High risk of thrombosis, including atrial fibrillation, pacemaker and stent implantation
Interventions	TXA, IV; n = 60 TXA, oral (2 g ); n = 60 TXA, topical; n = 60 TXA, oral (various doses); n = 60
Outcomes	Blood measurement, function, inflammation marker, anticoagulation marker, fibrinolysis marker
Starting date	
Contact information	
Notes	No response as of 13 August 2021 to multiple emails sent to authors asking for an update on trial status From 1 May 2018 to 31 August 2018

**Table:** ChiCTR 1800018334

Study name	
Methods	RCT
Participants	Patients with pelvic fracture Patients of acetabular fracture
Interventions	TXA, IV vs saline, IV
Outcomes	Blood loss, blood transfusion, Hb decline, haematocrit decline, erythrocyte concentration decline, thrombotic event, wound complications, length of stay, hospitalisation expenses, mortality, readmission rate
Starting date	
Contact information	
Notes	No response as of 13 August 2021 to multiple emails sent to authors asking for an update on trial status

**Table:** ChiCTR 1900021948

Study name	
Methods	RCT
Participants	Consecutive patients with a diagnosis of hip fractures treated at our hospital by any surgical procedure Aged > 18 years Preoperative anaemia
Interventions	TXA + iron Placebo + iron TXA + placebo Placebo + placebo Iron delivered via IV; unclear route of administration for TXA
Outcomes	Transfusion rate, transfusion amount, hidden blood loss, total blood loss, Hb level, Hb drop, proportion of anaemic participants, fibrinolysis index, reticulocyte count, blood management costs, length of hospital stay, thrombotic events, wound complication, blood transfusion‐related events, unplanned readmission rate, mortality rate
Starting date	
Contact information	
Notes	No response as of 13 August 2021 to multiple emails sent to authors asking for an update on trial status

**Table:** ChiCTR 2000032758

Study name	Defining the optimal perioperative regimen of intravenous TXA in patients with hip fracture: a prospective, randomized, double‐blind, controlled study
Methods	RCT, parallel, double‐blind
Participants	**Inclusion criteria** Patients with unilateral hip fractures (femoral neck fracture, intertrochanteric and subtrochanteric fractures) who underwent surgical treatment Age ≥ 18 years No abnormality was found on preoperative venous ultrasonography of both lower extremities Preoperative platelet and coagulation functions were normal Patients who voluntarily participated in clinical trials and signed informed consent, with good compliance **Exclusion criteria** Clearly allergic to TXA or contraindicated The time from injury to hospital admission is > 24 h Open fracture or pathological fracture or periprosthetic fracture Multiple trauma or fracture Taking anticoagulants or aspirin for < 1 week Patients at high risk of thrombosis, including atrial fibrillation, pacemaker and stent implantation VTE, PE, cerebral history of infarction and coronary heart disease (within half a year) Abnormal platelet and coagulation function (PLT< 100*10^9, INR > 1.4)
Interventions	**A**: **placebo (saline)** n = 30 Before surgery (before skin incision), 3 and 6 h after surgery with equal volume of NS **B: TXA (IV)** n = 30 TXA (TXA) was 15 mg/kg before surgery (before incision) and the same amount of NS 3 and 6 h after surgery **C: TXA (IV)** n = 30 Before surgery (before incision), 3 h after surgery, TXA was 15 mg/kg, and 6 h after surgery, the same amount of NS D: **TXA (IV)** n = 30 Before surgery (before skin incision), 3 and 6 h after surgery, TXA 15 mg/kg
Outcomes	**Primary** Total blood loss Hidden blood loss **Secondary** Intra‐op blood loss Blood transfusion rate VTE
Starting date	Expected from 31 July 2020 to 31 May 2021
Contact information	Country: China Contact for registration application: Chen Ran Applicant E‐mail: chenran15@hotmail.com Applicant telephone: +86 13008135085 Mailing address of the contact person for registration: No. 10, Daping Changjiang Branch Road, Yuzhong District, Chongqing
Notes	Not yet approved by an ethics committee (Date of Last Refreshed on: 09 May 2020)

**Table:** ChiCTR‐ICC‐15006070

Study name	
Methods	RCT
Participants	Patients with pelvic fractures who need to undergo internal fixation operation Between 18‐70 years old Patients who have given informed consent
Interventions	TXA, IV vs saline, IV
Outcomes	The volume of blood loss during operation The volume of drainage postoperative The volume of blood transfusion, Hb, fibrinogen, D‐ dimer, INR, PT, APTT
Starting date	
Contact information	
Notes	No response as of 13 August 2021 to multiple emails sent to authors asking for an update on trial status

**Table:** ChiCTR‐IPR‐17013477

Study name	
Methods	RCT
Participants	Aged > 18 years old ASA I‐III Undergoing internal fixation of spine, acetabular fractures, femoral shaft fracture, pelvic fracture, humeral shaft fracture and proximal humeral fractures as well as revision of total hip arthroplasty and total hip replacement of high congenital dislocation of the hip
Interventions	Continuous administration of TXA Intermittent administration of TXA No TXA (standard care, not relevant to this review)
Outcomes	Intraoperative blood loss, autologous blood transfusion, DVT, allogeneic blood transfusion, coagulation function, TEG, platelet function
Starting date	
Contact information	
Notes	No response as of 13 August 2021 to multiple emails sent to authors asking for an update on trial status

**Table:** CTRI/2019/04/018735

Study name	
Methods	RCT
Participants	Patients aged 18‐65 years ASA I‐II Patients undergoing surgery for pelviacetabular fracture under regional anaesthesia
Interventions	Bolus of TXA 1 g over 10 min vs bolus of TXA 10 mg/kg bolus, over 10 min followed by continuous infusion of 1 mg/kg/h for 4 h
Outcomes	Total blood loss, total blood transfusion, adverse effects due to TXA, incidence of DVT
Starting date	
Contact information	
Notes	Author responded 5 August 2021 saying they are planning to publish soon

**Table:** CTRI/2019/09/021302

Study name	
Methods	RCT
Participants	All consecutive patients requiring open reduction surgeries from orthopedic ward All patients giving informed consent
Interventions	TXA, IV vs saline, IV
Outcomes	Assess the effect of TXA in decrease in blood loss, compare Hb reduction, requirement of blood transfusions
Starting date	
Contact information	
Notes	RS has been trying to access trial page but hyperlink has not been working. Last attempt to access trial page was on 13 August 2021 Rita S: author replied saying that trial has not yet been published, but she has emailed us a Powerpoint presentation with some trial results 15 November 2021

**Table:** CTRI/2019/10/021667

Study name	Role of TXA in reducing blood loss in hip fracture surgeries
Methods	Randomised, parallel‐group, placebo‐controlled trial
Participants	Evans types 1 and 2 Internal fixation by dynamic hip screw
Interventions	**IV TXA** 2 doses of slow infusion of TXA 10 mg/kg body weight, 10 min prior to incision and at 2 h from first dose vs local TXA: 1 g of TXA intramuscularly at the end of skin incision during exposure, another 1 g into the femoral neck and head after triple reaming and another 1 g intramuscularly and subfascially prior to closure **Control group** Standard haemostatic measures only
Outcomes	Hb fall on day 5 after accounting for intraoperative and post‐operative transfusion, any complications, number of packed cells transfused postoperatively, total, visible and hidden blood
Starting date	01 November 2019
Contact information	Dr Koushik Narayan Subramanyam: drkoushik@hotmail.com
Notes	Authors state that recruitment has been delayed due to personnel issues and COVID. Still ongoing

**Table:** CTRI/2021/09/036855

Study name	Evaluation of efficacy of TXA on blood loss in periarticular hip surgeries
Methods	Randomised, parallel‐group, placebo‐controlled trial **Method of generating random sequence** Coin toss, lottery, toss of dice, shuffling cards etc **Method of** c**oncealment** Not applicable **Blinding/masking** Not applicable
Participants	**Inclusion criteria** Age 18‐75 years Patients requiring major periarticular hip surgeries **Exclusion criteria** Psychiatric patients Any abnormal bleeding disorder like haemophilias, deranged INR Any previous history of adverse reaction or allergy to TXA Congenital or acquired coagulopathy Recent history of thromboembolic episode Fracture neck of femur requiring closed reduction internal fixation with cannulated cancellous screws Implant removal procedure
Interventions	**Placebo ** Tablets containing calcium given orally 2 h prior to incision. 20 mL NS given through drain post‐operatively **TXA ** Tablets containing 1950 mg of TXA given 2 h prior to incision for surgery and 2 g of injection TXA diluted in 20 mL normal saline given through post‐operative drain
Outcomes	**Primary** o**utcome** To assess the efficacy of TXA in reducing intra‐ and post‐operative blood loss volume (during first 48 h) in patients undergoing periarticular hip surgeries **Secondary** o**utcome** To assess the reduction of requirement of blood transfusion both intra‐operative and during first 48 h post‐surgery To assess any thromboembolic events **Time points:** first 48 h post‐surgery, 3 months post‐surgery
Starting date	**Date of first enrolment (India)** 30 September 2021
Contact information	**Name ** Dr Rakesh Kumar Gupta **Designation ** Senior professor **Affiliation ** Pt BD Sharma PGIMS Rohtak **Address ** Department of orthopaedics pt BD Sharma PGIMS Rohtak Rohtak HARYANA 124001 India **Phone ** 9896297534 **Email ** drrk60@rediffmail.com
Notes	VG asked about % of elective vs % of trauma. Author replied 1 June 2022 saying that 83 out of 100 participants were trauma Postgraduate thesis **Primary sponsor** **Name ** Pt BD Sharma PGIMS Rohtak **Address ** Deptt of orthopaedics Pt BD Sharma PGIMS Rohtak **Type of sponsor ** Government medical college

**Table:** EUCTR 2011‐006278‐15

Study name	
Methods	RCT
Participants	Patients > 18 years Patients with unilateral subcapital femoral fracture Patients requiring hip replacement (total or partial) Signature of informed consent from the patient or their legal representative
Interventions	TXA vs fibrin glue
Outcomes	To assess whether TXA or fibrin glue administered topically reduced blood loss by at least 25% with respect to control in participants undergoing subcapital fracture of the femur, hidden blood loss, proportion of participants requiring blood transfusion in the postoperative, preoperative and postoperative Hb, number of blood transfusions, units of blood transfusions administered, incidence of wound infection, pain patient's surgical wound, days in hospital, related side effects
Starting date	
Contact information	
Notes	The trial hyperlink is now working (after a period of not working), the link was last checked on 16 June 2021. The status says the trial is ongoing, but this hasn't been updated since 2012. RS emailed the contact author using the email address given but this email bounced back. 16 June 2021

**Table:** EUCTR 2018‐000528‐32

Study name	
Methods	RCT
Participants	Age > 64 Femur fracture that needs surgical treatment
Interventions	TXA, IV vs placebo
Outcomes	Reduction in the number of patients who need a RBC transfusion after femur fracture
Starting date	
Contact information	
Notes	The trial hyperlink is now working (after a period of not working), the link was last checked on 16 June 2021. The status says the trial is ongoing, but this hasn't been updated since 2018. No email address or contact information for trial author. So we might be unable to get an update on the status.

**Table:** IRCT 2017 1030037093N18

Study name	
Methods	RCT (parallel), Not blinded Target sample size: 60
Participants	Ages 16‐65 years ASA Class 1 and 2 **Exclusion criteria** History of coagulopathy and bleeding disorders Renal impairment History of using antiplatelet and anticoagulant Acute infection A history of malignancy and ASA > 2 and thromboembolic events Hb levels < 10 Sensitivity to TXA
Interventions	The TXA‐receiving group was injected intravenously 30 min before surgery with a 15 mg /kg dose of TXA. vs In control group, only NS was injected with equal volume of 200 mL for 20 min
Outcomes	Bleeding rate, blood transfusion rate, Hb level
Starting date	
Contact information	
Notes	Target sample size: 60 Recruitment status: recruiting (Last refreshed on: 7 October 2019) **Registrant information** Name Sadra Ansaripour Name of organisation /entity Shahrekord University of Medical Sciences Country Iran (Islamic Republic of) Phone +98 31 3650 3487 Email address st_ansari.s@skums.ac.ir **Sponsor** Name of organisation /entity Bandare‐abbas University of Medical Sciences Full name of responsible person Abdul Azim Nejati Zadeh Street address Deputy of research and technology, East Side, Bandar Abbas Hospital, Bandar Abbas City Bandar Abbas Province Hormozgan Postal code 9791991551 Phone +98 71 3333 5794 Email azimnejate @yahoo.com

**Table:** IRCT 2020 0109046064N1

Study name	The effect of prophylactic fibrinogen infusion on intraoperative bleeding during pelvic surgery
Methods	A randomised controlled clinical trial with parallel, double‐blind, randomised groups Groups that have been masked Participant Outcome assessor
Participants	Target sample size: 42 **Inclusion criteria** Patients candidate for non‐emergency pelvic surgery undergoing general anaesthesia Satisfaction with study participation Age between 18‐60 years BMI < 30 The preoperative fibrinogen level should be 2 g/L‐4 g/L **Exclusion criteria** Cardiovascular, liver, kidney, coagulation and hypertension disorders Diabetes and tumours in surgical areas History of use the beta‐blocker, calcium blocker, digoxin, tricyclic antidepressants, anti‐coagulant and clonidine History of alcohol or drug abuse
Interventions	44 participants were randomly divided into 21 groups of **fibrinogen and placebo**. Hb, platelet and fibrinogen levels are measured in all patients before surgery. In the intervention group after induction, 1 g of fibrinogen injected and the control group injected with a similar volume in mL of NS.
Outcomes	**Primary** o**utcome(s)** Plasma fibrinogen Timepoint: before and after surgery Method of measurement: based on the participant's blood laboratory results
Starting date	Date of first enrolment: 20 February 2020
Contact information	**Registrant information** Name: Majid Charosaei Iran (Islamic Republic of) Phone: +98 21 4407 6824 Email address: drch128@gmail.com
Notes	Recruitment status: recruiting (Last refreshed on: 24 February 2020)

**Table:** Liu 2021

Study name	Hemostatic efficacy and safety of preemptive antifibrinolysis with multi‐dose intravenous TXA in elderly hip fracture patients: a prospective randomized controlled trial
Methods	RCT (parallel)
Participants	**Inclusion criteria** Aged > 65 years Diagnosed with a primary, unilateral, recent hip fracture (femoral neck fracture or intertrochanteric fracture) by X‐ray or CT scan Receiving hemi‐ or total hip arthroplasty **Exclusion criteria** Cognitive dysfunction, inability to obtain informed consent, or rejection of participants Multiple fractures or open fractures Active bleeding (such as active gastrointestinal bleeding, cerebral haemorrhage, etc.) Systemic thromboembolism (DVT, PE, etc.) Coagulation dysfunction Severe neuromuscular disease Allergic to TXA
Interventions	**Placebo** Group A, n = 40 NS 100 mL IV every 12 h, before surgery; 1.5 g TXA IV every 12 h, the first 3 days following surgery **TXA (IV)** Group B, n = 40 1.5 g TXA IV every 12 h, before surgery; 1.5 g TXA IV every 12 h, the first 3 days following surgery
Outcomes	**Primary outcomes** Total blood loss hidden blood loss Dominant blood loss Decline of Hb **Secondary** Alloegeneic blood transfusion rate Inflammatory factors Wound complications Length of stay 90‐day mortality Incidence of VTE (DVT and PE)
Starting date	Date of approved by ethic committee：29 April 2020 Study execute time: from 01 June 2021 to 01 September 2022 Recruiting time: from 01 June 2021 to 31 May 2022
Contact information	Applicant: Liu Jiacheng Study leader: Huang Wei Applicant telephone: +86 15823906402 Study leader's telephone: +86 13883383330 ： Applicant email: jiacheng‐96@qq.com Study leader's email: huangwei68@263.net Address: 1 Youyi Road, Yuanjiagang, Yuzhong District, Chongqing, China Institution: The First Affiliated Hospital of Chongqing Medical University
Notes	Registration number: ChiCTR2100045960

**Table:** NCT02428868

Study name	
Methods	RCT
Participants	Patients undergoing hip fracture surgery within 72 h after trauma
Interventions	TXA + iron vs TXA vs placebo
Outcomes	Transfusion, average red‐cell packs per participant, blood loss, Hb level, thromboembolic events, post‐operative bacterial infection, number of days in hospital, functional mobility, mortality
Starting date	
Contact information	
Notes	No response as of 13 August 2021 to multiple emails sent to authors asking for an update on trial status

**Table:** NCT02938962

Study name	
Methods	RCT
Participants	Age ≥ 18 years at the time of surgery Consent for transfusion of blood or blood‐related products No contraindication to use of TXA Revision hip arthroplasty performed at study location (Mount Sinai Hospital) Indication for surgery including osteolysis, component failure, prosthetic joint infection, aseptic/septic loosening, periprosthetic fracture, recurrent instability/dislocation, polyethylene wear and abductor insufficiency Revision hip arthroplasty procedure performed including acetabular component revision, femoral component revision, impaction bone grafting, proximal femoral allograft, proximal femoral replacement, removal of hardware (excluding head/liner exchanges) Direct lateral (transgluteal, Hardinge) approach utilised, including augmentation with extended trochanteric osteotomy (ETO), trochanteric slide and modified trochanteric slide
Interventions	TXA, IV vs TXA, topical
Outcomes	Change in Hb, allogeneic blood units transfused, length of hospital stay, estimated intra‐operative blood loss, post‐operative complications
Starting date	
Contact information	
Notes	Several emails have been sent to trial author to confirm if study population included revision hip operations performed for periprosthetic hip fractures. However, no response has been received.

**Table:** NCT02972294 (HiFIT)

Study name	HiFIT Study: Hip fracture: iron and tranexamic acid (HiFIT)
Methods	RCT
Participants	Age ≥ 18 years Osteoporotic fractures of the upper end of the femur requiring surgical repair Preoperative Hb between 9.5 and 13 g/dL Patient or relative signed informed consent or inclusion thanks to urgent inclusion procedure
Interventions	Iron isomaltoside 1000 vs TXA vs placebo iron isomaltoside 1000 vs placebo TXA
Outcomes	Proportion of participants who received a blood transfusion during their hospital stay following surgery Proportion of participants who received a blood transfusion after surgery Number of packed RBC units transfused per participant, as well as number of fresh frozen plasma and platelets units Hb concentration Proportion of participants with anaemia Reticulocytes count Perioperative blood loss Post‐operative Iron deficiency rate Number of hospitalisation days Proportion of participants at home Proportion of participants able to walk a distance of 10 feet (approx 3.5 m) without assistance Variation of quality of life Variation of perceived quality of life Variation of IADL test Death rate from all causes Rate of AEs including the following clinical complications: Vascular events Heart failure Renal failure Infectious complications Anaphylactic reaction Transfusion‐related complications Strength assessed by the Hand Grip Strength test Muscular fatigability assessed by the Hand Grip Strength test Level of locomotion and balance assessed by the Timed " Up and Go " test
Starting date	Study start date: March 2017
Contact information	Study Director: Sigismond SL Lasocki, Universite Hospital, Angers
Notes	RC accessed trial page 3 August 2021, status is 'active ‐ but not recruiting. This status was updated July 2021. **EUCTR trial registration states this study ended prematurely.**

**Table:** NCT03063892

Study name	
Methods	RCT, parallel, quadruple‐blind
Participants	Aged > 60 years Hip fracture requiring surgical intervention Signs consent and agrees to participate
Interventions	**Placebo comparator: control arm** Will receive IV saline solution placebo bolus dose in the Emergency Center over 10 min. The participant will also receive IV saline solution over 8 h prior to surgery. Another dose will be administered at the time of incision and the final dose 3 h later **Intervention: drug: saline solution** **Experimental** IV TXA 15 mg/kg (maximum 1 g) bolus dose over 10 min in the Emergency Center, plus an IV dose of TXA 15 mg/kg over 8 h prior to surgery. Another 15 mg/kg dose of TXA administered over 10 min at the time of incision and the final dose (15 mg/kg) of IV TXA over 10 min 3 h later
Outcomes	Proportion of participants requiring packed RBC transfusion, intraoperative blood loss, postoperative anaemia
Starting date	
Contact information	
Notes	**Estimated primary completion date 1 June 2023** Contact: Sara Seegert, MSN, RN 419‐291‐3441 sara.seegert@promedica.org Contact: Michelle Barhite, RPh 419‐291‐7709 michelle.barhite@promedica.org Country: USA

**Table:** NCT03182751

Study name	Does early administration of tranexamic acid reduce blood loss and perioperative transfusion requirement
Methods	RCT (parallel) Study type: interventional (clinical trial) Estimated enrolment: 156 participants
Participants	AO/OTA fracture classification 31A Surgically treated with sliding hip screw or cephalomedullary nail (short or long) Low energy, isolated injury Age > 18 years **Exclusion criteria** Intracapsular hip fractures: AO/OTA fracture classification 31B‐C Polytrauma participants Creatinine clearance < 30 mL/min History of unprovoked VTE and/or recurrent VTE Known history of Factor V Leiden, protein C/S deficiency, prothrombin gene mutation, anti‐thrombin deficiency, anti‐phospholipid antibody syndrome, lupus anticoagulant Pregnancy or breastfeeding (pregnancy tests will be performed on all patients of child‐bearing potential) History of CVA, MI, or VTE within the previous 30 days Coronary stent placement within the previous 6 months Disseminated intravascular coagulation Intracranial haemorrhage
Interventions	TXA, IV vs placebo **Active comparator: TXA** TXA will be administered IV via bolus dose of 1 g over 10 min and an additional 1 g over the subsequent 8 h Drug: TXA Other name: Cyklokapron **Placebo comparator: control arm** Patients in the control group will receive a placebo medication in the Emergency Department. Neither group will receive perioperative bolus dosing of TXA
Outcomes	Proportion of participants transfused at least 1 unit of packed RBCs, mean number of units transfused per participant, calculated blood loss, incidence of symptomatic VTE, wound complications, MI diagnosed, CVA diagnosed, all‐cause mortality
Starting date	April 2018
Contact information	Chelsea Boe: boe.chelsea@mayo.edu; Elsa Chase: chase.elsa@mayo.edu
Notes	**Last update posted**: **2 November 2021** Recruitment status: recruiting Estimated primary completion date: 1 December 2022 Estimated study completion date: 1 December 2022

**Table:** NCT03211286

Study name	Effect of intravenous tranexamic acid on reduction of blood losses in hip fracture patients
Methods	RCT, parallel, quadruple‐blind Actual enrolment (submitted: 6 April 2022): 129
Participants	Consecutive patients with a diagnosis of hip fractures treated at studyhospital by any surgical procedure Age over 60 years **Exclusion criteria** ASA IV Concomitant fracture Refusal to receive blood products Preoperative anaemia needing blood transfusion before surgery Severe comorbidity (cancer, severe pulmonary disease) Allergy for TXA History of acute thromboembolic event (DVT, PE, stroke) Coagulopathy (INR > 1.4) MI in the previous 12 months Coronary stents Renal function impairment (serum creatinine > 2 mg/dL or creatinine clearance < 30 mL/min),) or kidney transplant Platelet antiaggregant treatment in the week before surgery Severe hepatic dysfunction (AST/ALT > 60) History of hypercoagulability Acquired disturbances of colour vision Occurrence intraoperative surgical/medical/anaesthetic complications
Interventions	**Drug: TXA** 1 g IV TXA in 100 mL of saline solution at the time of surgical incision **Drug: saline solution** IV saline solution 100 mL at the time of surgical incision
Outcomes	Blood transfusion rate Perioperative blood loss Infection rate Thrombotic events Mortality
Starting date	Study start date: January 2018
Contact information	Principal investigator: Alejandro Lizaur‐Utrilla lizaur1@telefonica.net
Notes	**Last update posted: 7 April 2022** Recruitment status: completed Actual study completion date: 9 March 2022

**Table:** NCT03923959

Study name	TAHFT
Methods	RCT Allocation: randomised Intervention model: parallel assignment Intervention model description: a prospective, double‐blinded, randomised study in the geriatric hip fracture population comparing those who receive IV TXA prior to incision to those who receive a placebo. Masking: quadruple (participant, care provider, investigator, outcome assessor) Masking description: all or pharmacists are un‐blinded to participant randomisation
Participants	Provision of written informed consent Age ≥ to 65 years Hip fracture location within the femoral neck, intertrochanteric, and subtrochanteric regions Indication for one of the following surgical interventions: hemiarthroplasty, total hip replacement, sliding plate and screw fixation, or intramedullary fixation **Exclusion criteria** Indication for closed reduction or percutaneous screw Allergy to TXA CVA/stroke, active coronary disease/MI, or DVT/PE within 1 month of the fracture Presence of hypercoagulable disorder
Interventions	**Active comparator: Intervention** 100 mL NS with 1 g of TXA in solution Intervention: drug: TXA injectable solution **Placebo comparator: placebo** 100 mL NS
Outcomes	Blood transfusions Complication rate Hospital readmission Mortality rate
Starting date	Actual study start date: 1 June 2019
Contact information	Principal investigator: Gregory Tocks, DO Penn Medicine /Lancaster General Hospital
Notes	Page last accessed 31 August 2021, status was "enrollment by invitation" Estimated primary completion date: 1 August 2022 **Estimated study** c**ompletion date: 1 January 2023** Country: USA

**Table:** TCTR 2021 0311001

Study name	The effect of intravenous tranexamic acid to reduce blood loss in non union shaft humerus fracture patient undergoing open reduction and plating randomized control trial
Methods	RCT, parallel, masked (allocation concealment) Planned sample size: 30
Participants	**Inclusion criteria** Diagnosis non‐union midshaft humerus patient undergoing open reduction and plating Aged ≥ 15 years **Exclusion criteria** History thromboembolism Patient taking anticoagulant or antiplatelet Patient with renal disease glomerular filtration rate < 60 Pathologic fracture Infection at fracture area
Interventions	**TXA (IV)** IV TXA 750 mg 15 min before surgery **Placebo** 15 min before surgery
Outcomes	Primary: total blood loss Secondary: blood transfusion
Starting date	Studys start date (first enrolment): 23 June 2021 (anticipated)
Contact information	Pornpanit Dissaneewate MD Phone: 074451601 Email: Dpornpanit@yahoo.com chanon thassanaleelaporn MD Phone: 074451601 Email: c.pond21@hotmail.com
Notes	Sponsor ID/IRB ID/EC ID: 61‐423‐11‐1 Ethics Review: Approval Number:REC.61‐423‐11‐1 Date of Approval: 18 June 2019 Sponsor: Faculty of Medicine, Prince of Songkla University Sponsor contact: Chanon Thassanaleelaporn Organisation: Prince of Songkla University Phone: 074451149 Business email: c.pond21@hotmail.com

**Table:** TCTR 2021 0316006

Study name	Tranexamic acid in displaced femoral neck fracture treated with bipolar hemiarthroplasty: a randomized, controlled trial of topical versus intravenous administration
Methods	RCT (parallel), open‐label Planned sample size: 130
Participants	**Inclusion criteria** Diagnosis fracture neck of femur Age > 60 years old Household ambulatory Low energy mechanism Complete inform consent **Exclusion criteria** Allergy to TXA Prior history of thromboembolic disease Prior history of stroke Prior history of ischaemic heart disease Prior history of seizure Congenital or acquired coagulopathy Renal or liver dysfunction
Interventions	Topical TXA group, participants get IV NS 100 mL drip in 5 min before surgery (placebo). Topical TXA, mixed tranexamic 3 g in NS 100 mL divide 50 mL put in femoral canal after femoral neck cut about 3 min and last 50 mL injected under fascia after closed wound
Outcomes	Primary: blood loss Secondary: blood transfusion, complications
Starting date	Study start date (first enrolment): 01 June 2021 (anticipated)
Contact information	Sarun Tantavisut Organization: Chulalongkorn University Phone: 0817354219 Business email: stantavisut@gmail.com
Notes	Sponsor ID/IRB ID/EC ID: 396/63 **Ethics Review:** Approval Number: 1523/2020 Date of Approval: 17 December 2020 **Sponsor:** Ratchasapisek Sompoch Name/Official Title: Sarun Tantavisut Organization: Chulalongkorn University Phone: 0817354219 Business email: stantavisut@gmail.com

**AE:** adverse event; **ALT:** alanine transaminase; **AO/OTA:** Arbeitsgemeinschaft für Osteosynthesefragen/Orthopaedic Trauma Association**; APTT:** activated partial thromboplastin time; **ASA:** American Society of Anesthesiologists; **AST:** aspartate transaminase; **CT:** computed tomography; **CVA:** cerebrovascular accident; **DVT:** deep vein thrombosis; **Hb:** haemoglobin; **IADL:** Instrumental Activities of Daily Living; **INR:** international normalisation ratio; **IV:** intravenous; **MI:** myocardial infarction; **NS:** normal saline; **NSAID:** nonsteroidal anti‐inflammatory drug; **PE:** pulmonary embolism; **PFNA:** proximal femoral nail anti‐rotation; **PT:** prothrombin time; **RBC:** red blood cell; **TEG:** thromboelastography; **TXA:** tranexamic acid **VTE:** venous thromboembolism

## Differences between protocol and review

### Differences due to insufficient data

#### Network meta‐analysis

In a deviation from our protocol, we have not performed a network meta‐analysis in this version, and have instead presented direct pairwise analyses only. This was due to the lack of usable data, contributing to few nodes of interest. Any future updates that have sufficient data will perform the network meta‐analysis as described in Appendix 1. 

As a result of only undertaking pairwise analyses, we have presented the data as risk ratio (RR), risk difference (RD) when zero cases were reported in both arms, or Peto odds ratio (Peto OR) where cases were rare (less than 5% per arm). We have used a random‐effects model for all analyses (except Peto OR), as reported in the *Cochrane Handbook for Systematic Reviews of Interventions* (Deeks 2022).

#### Missing data

Where we identified data as being missing or unclear in the published literature, we contacted trial authors directly. We then planned that if we were still unable to obtain the information, and the missing data were thought to lead to serious bias, we would perform a sensitivity analysis to assess the impact of the missing outcome data. We did not perform a sensitivity analysis as we did not have sufficient data present to assess what may be missing.

#### Continuous outcomes

None of the included studies reported our continuous outcomes in an analysable format (reported as median interquartile/range). For future updates, we will analyse continuous outcome data measured using the same scale using mean difference (MD) with a 95% confidence interval (CI). However, if studies measure this outcome using different scales, we will use standardised mean difference (SMD) with 95% CI.

#### Assessment of reporting biases

No meta‐analysis in this review included at least 10 trials, we therefore could not perform a formal assessment of publication bias (Page 2022).

#### Subgroup analyses

There were insufficient data to perform all the planned subgroup analyses. In future updates, if the data allow, we will perform subgroup analyses and network meta‐regression for the following variables, to explain any heterogeneity, inconsistency, or both, across all outcomes:

- type of surgery;
- participants with preoperative anaemia;
- participants on anticoagulant or antiplatelet therapy at the time of injury.

See Data extraction and management for more information.

#### Sensitivity analyses

Using the information generated, we looked for statistical heterogeneity in each trial and planned to perform sensitivity analyses accordingly. We planned to do this for the primary outcomes in the first instance, and then apply this to other outcomes with significant heterogeneity. However, we did not perform any sensitivity analyses due to the low heterogeneity between studies, and lack of data.

### Clarification of points within the protocol

We have clarified some points from the protocol that are relevant to future updates.

- Definition of ‘red cell transfusions up to 30 days post‐surgery (units)’ outcome: in the review, under this definition, we recorded the mean number of red blood cell transfusions.
- Definition of ‘N’ for mean number of transfusions: in the protocol it was not clearly explained that for the ‘mean number of transfusions’ outcome, we used N to describe the number of participants who were transfused, not number of participants in the arm.
- No thromboembolic events: many of the included publications reported that there were ‘no thromboembolic events’. We have taken that to mean that both pulmonary embolism and deep vein thrombosis events were zero.
- Intention to treat (ITT): some trial reports did not explicitly state whether they used ITT for the analysis. In these situations we looked at the numbers in the study flowchart (where available) as well as other reporting of participant flow, and the data to assess whether ITT was used.
- Definition of mean number of transfusions: when cleaning the data for this outcome in preparation for any network meta‐analyses, in future updates we will exclude any studies where the mean or standard deviations, or both, are zero. We took this decision following guidance from an experienced statistician (Prof N Welton). We were also advised to exclude mean number of transfusions data from any studies where the median and interquartile range data are skewed.
- Title change to include joint replacement for hip fractures: as part of the standard definitive treatment for hip fractures, we included joint replacement for hip fractures in this review. We therefore felt that a change to the title to include joint replacement would better reflect the content of this review. Our search was based on the injuries sustained rather than the surgery performed, and therefore we felt this would not affect the outcome of the search we performed.
- Summary of findings tables:
  - the protocol stated we would include the following outcomes in the SOF table:
    - risk of requiring allogeneic blood transfusion during or after surgery (within 30 days)
    - all‐cause mortality (deaths occurring within 30 days after the operation)
    - mean number of red blood cell transfusion units per person (within 30 days)
    - number of units of allogeneic blood transfused
    - reoperation due to bleeding (within seven days) and 
    - adverse events (within 30 days)
- This was changed to:
  - Risk of requiring allogeneic blood (no change)
  - All‐cause mortality (no change)
  - Re‐operation (no change)
  - Risk of myocardial infarction
  - Risk of cerebrovascular accident/stroke
  - Risk of deep vein thrombosis
  - Risk of suspected serious drug reaction
- As we are limited to seven outcomes in the summary of findings tables, and we did not analyse 'adverse events' as a single outcome, it was necessary to select which adverse events were deemed clinically most important. Likewise, we deemed the need for transfusion more important than the volume transfused, and so mean number of red blood cell transfusion units was not listed in the summary of findings tables. We have also clarified the previously listed mean red cell transfusion and number of units transfused, above.
